# Sorbicillinoids From the Fungus *Ustilaginoidea virens* and Their Phytotoxic, Cytotoxic, and Antimicrobial Activities

**DOI:** 10.3389/fchem.2019.00435

**Published:** 2019-06-12

**Authors:** Jiajia Meng, Gan Gu, Pengqin Dang, Xuping Zhang, Weixuan Wang, Jungui Dai, Yang Liu, Daowan Lai, Ligang Zhou

**Affiliations:** ^1^Department of Plant Pathology, College of Plant Protection, China Agricultural University, Beijing, China; ^2^State Key Laboratory of Bioactive Substance and Function of Natural Medicines, Institute of Materia Medica, Peking Union Medical College, Chinese Academy of Medical Science, Beijing, China; ^3^Institute of Food Science and Technology, Chinese Academy of Agricultural Sciences, Beijing, China

**Keywords:** rice false smut disease, *Ustilaginoidea virens*, sorbicillinoids, phytotoxic activity, cytotoxic activity

## Abstract

*Ustilaginoidea virens*, the causal fungus of rice false smut, was found in previous studies to produce two types of metabolites, ustiloxins and ustilaginoidins. However, genome sequencing of *U. virens* revealed a plethora of secondary-metabolites-biosynthetic core genes that were capable to biosynthesize unreported metabolites. A large-scale fermentation of *U. virens* was thus performed, and the fungal extract was chemically re-investigated. After removing the known metabolites, we found a fraction containing unknown phytotoxic substances. Fractionation of this part has led to the isolation of six new sorbicillinoids, namely ustisorbicillinols A~F (**1**~**6**), and two new sorbicillinoid-related pyrones, named ustilopyrones A (**7**) and B (**8**), together with nine known cogeners (**9**~**17**). The structures of the new compounds were elucidated by analysis of their NMR, HRMS, and CD spectra, while ECD, ^13^C NMR and optical rotation calculations were additionally used for configurational assignments. Plausible biosynthetic pathways for the new compounds were proposed. Phytotoxicity assays revealed that the major sorbicillinoids (**12**~**14**, and **16**) showed strong inhibition against the radicle and germ elongation of rice and lettuce seeds, with compound **12** displaying the strongest inhibition. The isolated compounds were also evaluated for their cytotoxic, antibacterial, and antifungal activities. Compounds **10**, and **12**~**14** showed moderate cytotoxicities against the tested cell lines with IC_50_s of 8.83~74.7 μM. Compounds **2**, and **10**~**13** were active against the tested bacteria (MICs 4~128 μg/mL), while compounds **11**~**13** displayed moderate antifungal activities.

## Introduction

Plant pathogenic fungi can produce toxic substances, known as phytotoxins, that adversely affect the host plants, and might play a role in the development of plant disease (Strange, 2007; Varejao et al., 2013). Understanding their roles in pathogenesis or virulence will help to find strategies to control diseases, such as breeding disease resistant crops (Strange, 2007). Meanwhile, the great structural diversity and potency of phytotoxins (Cimmino et al., 2015) offered remarkable potential for developing natural herbicides with new modes of action (Duke and Dayan, 2011; Dayan and Duke, 2014).

*Ustilaginoidea virens* (teleomorph: *Villosiclava virens*), the causal fungus of rice false smut, which is a destructive disease in the rice cultivation area worldwide (Zhang et al., 2014; Fan et al., 2016), uniquely infects the spikelets of rice panicles, and results in the formation of smut balls around the panicles. So far, two known types of phytotoxins, including the ustiloxins (13-membered-ring cyclopeptides), and ustilaginoidins (9,9′-linked bis-naphtho-γ-pyrones), have been found in the smut balls as well as in the cultures of the pathogen (Lu et al., 2015; Sun et al., 2017; Wang et al., 2017). However, genome sequencing of the pathogen *U. virens* revealed a plethora of secondary-metabolites-biosynthetic core genes (Zhang et al., 2014), including those encoding NRPS, PKS, and terpene synthases, indicating the great potential of this fungus to produce unknown secondary metabolites.

As part of our continuing interest in the secondary metabolites, especially the phytotoxins, of *U. virens* (Lu et al., 2015; Sun et al., 2017; Lai et al., 2019), we fermented this fungus on rice medium in a large scale (22.0 kg of rice). After removing the two known types of compounds mentioned above by size-exclusion chromatography over Sephadex LH-20, a fraction containing the third type of compounds that showed their maximal UV absorptions at about 370 nm was found, which indicated the presence of sorbicillinoids (Andrade et al., 1992; Lai et al., 2019). This fraction also exhibited interesting phytotoxic activities against the rice seeds (data not shown). A detailed chemical investigation of this part was performed, which led to the isolation of 17 sorbicillinoids, including eight new compounds. Herein, we report the isolation, structural elucidation of the new sorbicillinoids, as well as the biological activities of the isolated compounds. Their biosynthetic pathway was also discussed.

## Materials and Methods

### General Experimental Procedures

Optical rotations were recorded on a Rudolph Autopol III automatic polarimeter (Rudolph Research Analytical, Hackettstown, New Jersey). Ultraviolet (UV) spectra were recorded on a TU-1810 UV/vis spectrophotometer (Beijing Persee General Instrument Co., Ltd., Beijing, China). Circular dichroism (CD) spectra were recorded on a JASCO J-815 CD spectrometer (JASCO Corp., Tokyo, Japan). Infrared (IR) spectra were measured on a Thermo Nicolet Nexus 470 FT-IR spectrometer (Thermo Electron Scientific Instrument Crop., Madison, Wisconsin). High-resolution electrospray ionization mass spectrometry (HRESIMS) spectra were recorded on a LC 1260/Q-TOF-MS 6520 instrument (Agilent Technologies, Santa Clara, CA). ^1^H, ^13^C, and 2D NMR (^1^H-^1^H COSY, HSQC/HMQC, HMBC, and NOESY) spectra were measured on Bruker Avance 600 or 400 NMR spectrometers (Bruker BioSpin, Zürich, Switzerland). ^1^H and ^13^C NMR chemical shifts were expressed in δ (ppm) referring to the inner standard tetramethylsilane (TMS), and coupling constants in Hertz. HPLC-DAD analysis of the EtOAc extracts was performed on a Shimadzu LC-20A instrument equipping with a SPD-M20A photodiode array detector (Shimadzu Corp., Tokyo, Japan) using an analytical C_18_ column (250 × 4.6 mm i.d., 5 μm; Phenomenex Inc., Torrance, California). Semipreparative HPLC separation was carried out on a Lumtech instrument (Lumiere Tech. Ltd., Beijing, China) equipped with a K-501 pump (flow rate: 3 mL/min) and a K-2501 UV detector (detection was set at 370 nm) using a Luna-C18 column (250 × 10 mm i.d., 5 μm, Phenomenex Inc., Torrance, California) eluting with a mixture of MeOH and water (containing 0.02% TFA).

### Fungal Material

The fungal strain *U. virens* UV8b (Zhang et al., 2014) was kindly provided by Prof. Wenxian Sun (College of Plant Protection, China Agricultural University, China).

### Fermentation, Extraction, and Isolation

The strain *U. virens* UV8b was grown on potato dextrose agar (PDA, potato 200 g/L, dextrose 20 g/L, and agar 20 g/L) at 25°C for 10 days. Then, several agar plugs (0.5 × 0.5 cm) containing mycelia were added into a 250 mL Erlenmeyer flask in which 100 mL of potato dextrose broth (PDB, potato 200 g/L, and dextrose 20 g/L) was filled. The liquid culture was incubated in a rotatory shaker for 10 days at 150 rpm and 28°C to produce the seed culture, which was used to inoculate the rice medium (100 g of rice, 110 mL of water, in each 1,000 mL flask). The fermentation was carried out using a total of 22.0 kg of rice at 28°C under static condition in the dark for 60 days. After harvest, the culture was extracted with EtOAc for four times. The EtOAc extract was combined and condensed under vacuum using a rotatory evaporator to yield a brownish residue (220 g). The extract was divided into 40 portions, and each portion was subjected to gel permeation chromatography over Sephadex LH-20 (i.d. 4.0 × 80 cm) eluting with CH_2_Cl_2_/MeOH (1:1) to obtain three fractions, the second of which was the sorbicillinoid-containing fraction (SF).

The SF (60 g) was subjected to vacuum liquid chromatograph over silica gel (200~300 mesh, i.d. 8 × 16 cm), eluting with a gradient of CH_2_Cl_2_/MeOH (100:0~0:100), to yield six fractions (Frs. A~F).

Fr. B was further fractionated by medium pressure liquid chromatography over silica gel (i.d. 4.0 × 50 cm) to obtain ten subfractions (Frs. B1~B10). Compound **17** (6.3 mg) was purified from Fr. B9 by semi-preparative HPLC eluting with 85% MeOH/H_2_O.

Fr. C was subjected to column chromatography (i.d. 3.0 × 60 cm) over Sephadex LH-20 (CH_2_Cl_2_/MeOH, 1:1) to yield seven subfractions (Frs. C1~C7), among which Fr. C5 was purified by semi-preparative HPLC (85% MeOH/H_2_O) to give oxosorbicillinol (**11**, 6.0 mg). Fr. C6 was further chromatographed (i.d. 4.0 × 40 cm) over RP-18 eluting with a mixture of MeOH/H_2_O (from 60:40 to 100:0, increased stepwisely) to afford six subfractions (Frs. C6-1~C6-6), among which Fr. C6-1 was purified by semi-preparative HPLC (70% MeOH/H_2_O) to yield **5** (2.6 mg) and **6** (1.0 mg).

Fr. D was fractionated by column chromatography (i.d. 4.0 × 40 cm) on RP-18 eluting with MeOH/H_2_O (gradually increased from 55:45 to 100:0) to obtain 12 subfractions (Frs. D1~D12). Fr. D7 was further chromatographed (i.d. 3.0 × 60 cm) over Sephadex LH-20 (CH_2_Cl_2_/MeOH, 1:1) to afford six fractions (Frs. D7.1~D7.6). Compounds **3** (2.2 mg), and **4** (2.7 mg) were obtained from Fr. D7.3, while compounds **10** (5.1 mg) and **15** (2.0 mg) were isolated from Fr. D7.5, by semi-preparative HPLC using 75% MeOH/H_2_O as the mobile phase. Likewise, compound **16** (20.1 mg) was purified from Fr. D7.6 (eluting with 80% MeOH/H_2_O). Fr. D8 was subjected to chromatography (i.d. 3.0 × 60 cm) over Sephadex LH-20 (CH_2_Cl_2_/MeOH, 1:1), followed by semi-preparative HPLC (80% MeOH/H_2_O) to give **1** (4.4 mg) and **2** (8.0 mg). Similarly, compounds **12** (22.3 mg), **13** (13.3 mg), and **14** (40.2 mg) were obtained by semi-preparative HPLC purification (85% MeOH/H_2_O) from Fr. D9 (for **13** and **14**), and Fr. D10 (for **12**), respectively.

Fr. E was chromatographed (i.d. 4.0 × 40 cm) over ODS eluting with a gradient increase of MeOH in water (from 30:70 to 100:0) to obtain six subfractions (Frs. E1~E6). The 2-pyrones, **7** (8.2 mg), **8** (1.7 mg) and **9** (3.9 mg) were isolated from Fr. E2 by semi-preparative HPLC using 45% MeOH/H_2_O as the mobile phase.

Ustisorbicillinol A (**1**). Yellow amorphous powder; ${\left[\alpha \right]}_{\text{D}}^{25}$ −356 (*c* 0.1, MeOH); UV(MeOH) λ_max_ (log ε) 220 (3.90), 271 (4.05), 379 (3.88) nm; ECD (c = 4.01 × 10^−4^ M, MeOH) λ (Δε) 372 (−7.92), 298 (+5.82), 290 (+5.65), 272 (+8.16), 218 (−6.93) nm; IR ν_max_ 3420, 2926, 2852, 1714, 1681, 1646, 1623, 1538, 1517, 1453, 1383, 1343, 1228, 1137, 1019, 903, 845, 675, 578 cm^−1^; ^1^H NMR (CD_3_OD, 400 MHz), ^13^C NMR (CD_3_OD, 100 MHz) see Table 1; HRESIMS *m*/*z* 497.2193 [M-H]^−^ (calcd for C_28_H_33_O_8_, 497.2181).

**Table 1:** ^1^H and ^13^C NMR data of **1**~**4**.

**Position**	**1**^**a**^		**2**^**a**^		**3**^**b**^		**4**^**b**^	
	*****δ***_**C**_, type**	*****δ***_**H**_, mult. (*J* in Hz)**	*****δ***_**C**_, type**	*****δ***_**H**_, mult. (*J* in Hz)**	**δ_**C**_, type**	*****δ***_**H**_, mult. (*J* in Hz)**	*****δ***_**C**_, type**	*****δ***_**H**_, mult. (*J* in Hz)**
1	33.1, CH_2_	2.55, d (15.2)2.26, d (15.2)	34.2, CH_2_	2.48, d (16.0)2.30, d (16.0)	55.4, CH	3.12, s	54.9, CH	3.12, s
2	74.5, C		74.2, C		79.0, C		79.2, C	
3	107.6, C		107.7, C		105.3, C		104.3, C	
4	57.7, C		57.8, C		55.7, CH	3.00, d (12.0)	56.0, CH	3.04, d (12.2)
5	172.1, C		171.6, C		170.8, C		172.3, C	
6	110.2, C		110.2, C		109.1, C		109.8, C	
7	193.8, C		194.3, C		188.3, C		188.7, C	
8	41.1, CH_2_	2.44, dd (17.0, 13.0)2.33, dd (17.0, 4.0)	42.3, CH_2_	2.23, dd (16.9, 3.6)2.12, dd (16.9, 14.7)	41.1, CH_2_	2.58, dd (16.7, 4.8)2.18, dd (16.7, 8.7)	41.7, CH_2_	2.56, dd (16.8, 13.4)2.29, dd (16.8, 3.5)
9	79.3, CH	4.26, ddd (13.0, 5.9, 4.0)	80.6, CH	4.56, m	80.2, CH	4.93, m	81.5, CH	4.38, ddd (13.4, 7.0, 3.5)
10	128.6, CH	5.47, dd (15.5, 5.9)	129.2, CH	5.45, dd (15.5, 6.3)	128.3, CH	5.43, ddq (15.2, 7.3, 1.7)	128.7, CH	5.57, ddq (15.0, 7.0, 1.7)
11	131.7, CH	5.80, m	130.7, CH	5.76, dq (15.5, 6.5)	131.4, CH	5.76, dqd (15.2, 6.6, 1.0)	132.0, CH	5.73, dqd (15.0, 6.6, 1.0)
12	18.0, CH_3_	1.72, d (6.3)	18.0, CH_3_	1.73, d (6.5)	17.9, CH_3_	1.65, ddd (6.6, 1.7, 0.7)	17.8, CH_3_	1.66, ddd (6.6, 1.7, 0.7)
13	22.3, CH_3_	1.11, s	22.6, CH_3_	1.17, s	20.9, CH_3_	1.27, s	20.9, CH_3_	1.25, s
14	19.7, CH_3_	1.32, s	19.1, CH_3_	1.29, s				
1′	53.9, CH	3.69, s	54.0, CH	3.69, s	48.6, CH	3.67, d (12.0)	48.5, CH	3.67, d (12.2)
2′	80.2, C		80.2, C		81.3, C		81.1, C	
3′	171.2, C		170.9, C		104.9, C		105.3, C	
4′	108.8, C		108.7, C		60.4, C		60.7, C	
5′	192.7, C		193.3, C		202.2, C		202.2, C	
6′	102.8, C		102.7, C		105.1, C		105.3, C	
7′	168.3, C		168.3, C		174.2, C		174.0, C	
8′	122.0, CH	6.45, d (14.8)	121.9, CH	6.45, d (14.8)	120.7, CH	6.56, d (14.8)	120.6, CH	6.53, d (14.8)
9′	139.4, CH	7.16, dd (14.8, 10.9)	139.3, CH	7.17, dd (14.8, 10.8)	142.6, CH	7.28, dd (14.8, 10.9)	142.6, CH	7.29, dd (14.8, 10.9)
10′	132.5, CH	6.33, dd (15.1, 10.9)	132.5, CH	6.32, dd (15.1, 10.8)	132.0, CH	6.38, m	132.0, CH	6.36, m
11′	137.4, CH	6.10, dq (15.1, 6.8)	137.3, CH	6.10, dq (15.1, 6.8)	139.5, CH	6.25, dq (15.1, 6.8)	139.6, CH	6.26, dq (15.0, 6.8)
12′	18.8, CH_3_	1.86, d (6.8)	18.8, CH_3_	1.86, d (6.8)	18.8, CH_3_	1.87, dd (6.8, 1.6)	18.8, CH_3_	1.87, dd (6.8, 1.6)
13′	26.6, CH_3_	1.46, s	26.6, CH_3_	1.46, s	21.5, CH_3_	1.35, s	21.5, CH_3_	1.36, s
14′	7.8, CH_3_	1.62, s	7.9, CH_3_	1.59, s	20.1, CH_3_	1.31, s	19.9, CH_3_	1.31, s

a Recorded in CD_3_OD,

b *Recorded in CD_3_COCD_3_*.

Ustisorbicillinol B (**2**). Yellow amorphous powder; ${\left[\alpha \right]}_{\text{D}}^{25}$ −188 (*c* 0.1, MeOH); UV(MeOH) λ_max_ (log ε) 219 (3.94), 270 (4.05), 381 (3.93) nm; ECD (c = 4.01 × 10^−4^ M, MeOH) λ (Δε) 369 (−8.08), 317 (+2.91), 270 (+10.86), 219 (−7.69) nm; IR ν_max_ 3390, 2932, 1713, 1681, 1646, 1623, 1566, 1517, 1472, 1384, 1229, 1209, 1137, 1019, 903, 847, 759, 678, 578 cm^−1^; ^1^H NMR (CD_3_OD, 400 MHz), ^13^C NMR (CD_3_OD, 100 MHz) see Table 1; HRESIMS *m*/*z* 497.2195 [M-H]^−^ (calcd for C_28_H_33_O_8_, 497.2181).

Ustisorbicillinol C (**3**). Yellow amorphous powder; ${\left[\alpha \right]}_{\text{D}}^{25}$ +28 (*c* 0.1, MeOH); UV(MeOH) λ_max_ (log ε) 214 (3.69), 278 (3.89), 360 (3.72) nm; ECD (c = 4.15 × 10^−4^ M, MeOH) λ (Δε) 368 (−0.26), 310 (+5.40), 272 (−4.78), 227 (+0.06) nm; IR ν_max_ 3726, 3421, 2922, 2852, 1734, 1717, 1695, 1653, 1617, 1576, 1455, 1442, 1384, 1306, 1199, 1131, 1019, 913, 840, 667, 579 cm^−1^; ^1^H NMR (CD_3_COCD_3_, 600 MHz), ^13^C NMR (CD_3_COCD_3_, 150 MHz) see Table 1; HRESIMS *m*/*z* 483.2006 [M+H]^+^ (calcd for C_27_H_31_O_8_, 483.2013).

Ustisorbicillinol D (**4**). Yellow amorphous powder; ${\left[\alpha \right]}_{\text{D}}^{25}$ −32 (*c* 0.1, MeOH); UV(MeOH) λ_max_ (log ε) 214 (3.63), 279 (3.88), 345 (3.79) nm; ECD (c = 4.15 × 10^−4^ M, MeOH) λ (Δε) 310 (+4.31), 270 (−9.06), 240 (−0.52), 228 (−0.85), 218 (−0.30) nm; IR ν_max_ 3727, 3385, 2936, 1654, 1616, 1439, 1383, 1305, 1256, 1192, 1128, 1050, 1006, 913, 862, 843, 580 cm^−1^; ^1^H NMR (CD_3_COCD_3_, 600 MHz), ^13^C NMR (CD_3_COCD_3_, 150 MHz) see Table 1; HRESIMS *m*/*z* 483.2006 [M+H]^+^ (calcd for C_27_H_31_O_8_, 483.2013).

Ustisorbicillinol E (**5**). Yellow amorphous powder; ${\left[\alpha \right]}_{\text{D}}^{25}$ +116 (*c* 0.1, MeOH); UV(MeOH) λ_max_ (log ε) 214 (3.46), 330 (3.66) nm; ECD (c = 6.02 × 10^−4^ M, MeOH) λ (Δε) 347 (+10.62), 308 (−12.16), 243 (−0.55), 214 (−3.01) nm; IR ν_max_ 3726, 3625, 3599, 3421, 2921, 2851, 1774, 1735, 1652, 1616, 1576, 1454, 1384, 1201, 1025, 912, 876, 580 cm^−1^; ^1^H NMR (CDCl_3_, 600 MHz), ^13^C NMR (CDCl_3_, 150 MHz) see Table 2; HRESIMS *m*/*z* 331.1201 [M-H]^−^ (calcd for C_18_H_19_O_6_, 331.1187).

**Table 2:** ^1^H (600MHz) and ^13^C (150MHz) NMR data of **5** (CDCl_3_).

**Position**	*****δ***_**C**_, type**	*****δ***_**H**_, mult. (*J* in Hz)**
1	45.5, CH	3.60, d (3.5)
2	74.2, C	
3	209.2, C	
4	62.5, C	
5	194.9, C	
6	104.7, C	
7	172.3, C	
8	117.2, CH	6.12, d (14.9)
9	144.6, CH	7.38, dd (14.8, 10.2)
10	130.8, CH	6.29, dd (15.3, 10.2)
11	141.4, CH	6.24, m
12	19.0, CH_3_	1.91, d (6.0)
13	24.4, CH_3_	1.28, s
14	10.4, CH_3_	1.23, s
1′	76.3, CH	5.44, dd (8.9, 3.5)
2′	41.4, CH	2.89, ddd (11.5, 8.9, 5.5)
3′	30.2, CH_2_	2.70, dd (19.2, 11.6) 2.24, dd (19.2, 5.4)
4′	174.8, C	
7-OH		14.32, s

Ustisorbicillinol F (**6**). Yellow amorphous powder; ${\left[\alpha \right]}_{\text{D}}^{25}$ −24 (*c* 0.05, MeOH); UV(MeOH) λ_max_ (log ε) 223 (3.53), 293 (3.40) nm; IR ν_max_ 3720, 3648, 3614, 3445, 2918, 2850, 1750, 1717, 1682, 1649, 1566, 1541, 1517, 1488, 1454, 1432, 1384, 1159, 1018, 900, 842, 677, 578 cm^−1^; ^1^H NMR (CDCl_3_, 600 MHz), ^13^C NMR (CDCl_3_, 150 MHz) see Table 3; HRESIMS *m*/*z* 311.0892 [M+Na]^+^ (calcd for C_16_H_16_NaO_5_, 311.0890).

**Table 3:** ^1^H (600MHz) and ^13^C (150MHz) NMR data of **6** (CDCl_3_).

**Position**	*****δ***_**C**_, type**	*****δ***_**H**_, mult. (*J* in Hz)**
1	109.7, C	
2	169.2, C	
3	73.7, C	
4	196.9, C	
5	106.1, C	
6	165.5, C	
7	180.5, C	
8	111.7, CH	6.31, s
9	163.9, C	
10	118.6, CH	6.12, d (15.4)
11	140.1, CH	7.24, ov.^a^
12	130.1, CH	6.26, ov.^b^
13	140.6, CH	6.27, ov.^b^
14	18.9, CH_3_	1.92, br. d (4.9)
15	30.0, CH_3_	1.64, s
16	6.8, CH_3_	1.88, s
6-OH		13.43, br. s

a *Overlapped with the solvent residual signal*.

b *Overlapped signals*.

Ustilopyrone A (**7**). Yellow amorphous powder; UV(MeOH) λ_max_ (log ε) 227 (3.97), 296 (3.74) nm; IR ν_max_ 3647, 3614, 3363, 2933, 1712, 1646, 1623, 1472, 1363, 1239, 1136, 1017, 909, 848, 756, 678, 578, 530 cm^−1^; ^1^H NMR (CD_3_OD, 400 MHz), ^13^C NMR (CD_3_OD, 100 MHz) see Table 4; HRESIMS *m*/*z* 221.0823 [M-H]^−^ (calcd for C_12_H_13_O_4_, 221.0819).

**Table 4:** ^1^H (400 MHz) and ^13^C (100 MHz) NMR data of **7**~**9** (CD_3_OD).

**Position**	**7**		**8**		**9**	
	*****δ***_**C**_, type**	*****δ***_**H**_, mult. (*J* in Hz)**	*****δ***_**C**_, type**	*****δ***_**H**_, mult. (*J* in Hz)**	*****δ***_**C**_, type**	*****δ***_**H**_, mult. (*J* in Hz)**
2	167.5, C^a^		167.03, C^b^		168.0, C	
3	100.6, C		102.3, C		100.8, C	
4	167.4, C^a^		166.95, C^b^		167.6, C	
5	110.5, C		113.7, C		102.3, CH	6.07, s
6	153.7, C		152.2, C		158.0, C	
7	120.7, CH	6.53, d (15.1)	128.2, CH	6.97, d (15.0)	123.2, CH	6.18, d (15.3)
8	135.4, CH	7.06, dd (15.1, 11.0)	132.2, CH	7.13, dd (15.0, 11.2)	135.6, CH	7.05, dd (15.3, 11.0)
9	130.5, CH	6.49, dd (15.0, 11.0)	144.6, CH	7.44, dd (15.2, 11.2)	129.8, CH	6.43, dd (15.2, 11.0)
10	139.2, CH	6.12, dt (15.0, 5.3)	126.0, CH	6.12, d (15.2)	140.0, CH	6.15, dt (15.2, 5.2)
11	63.1, CH_2_	4.19, d (5.3)	170.0, C		63.0, CH_2_	4.18, d (5.2)
3-Me	9.2, CH_3_	1.94, s	9.4, CH_3_	1.96, s	8.6, CH_3_	1.89, s
5-Me	9.6, CH_3_	2.04, s	9.9, CH_3_	2.09, s		

a, b *Assignments within a column maybe interchanged*.

Ustilopyrone B (**8**). Yellow amorphous powder; UV(MeOH) λ_max_ (log ε) 226 (3.71), 261 (3.91), 342 (3.64) nm; IR ν_max_ 3732, 3421, 2919, 2850, 1719, 1652, 1617, 1576, 1542, 1455, 1435, 1384, 1270, 1154, 1020, 835, 732, 579 cm^−1^; ^1^H NMR (CD_3_OD, 400 MHz), ^13^C NMR (CD_3_OD, 100 MHz) see Table 4; HRESIMS *m*/*z* 235.0617 [M-H]^−^ (calcd for C_12_H_11_O_5_, 235.0612).

5-Demethylustilopyrone A (**9**). Yellow amorphous powder; UV(MeOH) λ_max_ (log ε) 249 (4.02), 335 (3.76) nm; IR ν_max_ 3252, 2935, 1712, 1646, 1623, 1472, 1384, 1363, 1234, 1135, 1017, 756, 580 cm^−1^; ^1^H NMR (CD_3_OD, 400 MHz), ^13^C NMR (CD_3_OD, 100 MHz) see Table 4; HRESIMS *m*/*z* 207.0665 [M-H]^−^ (calcd for C_11_H_11_O_4_, 207.0663).

### ECD Calculation

The Molecular Merck force field (MMFF) conformational search, geometry optimization and frequency calculations at the B3LYP 6-31G(d) level *in vacuo*, and TDDFT ECD calculations of the dominant conformers (>1%) at the PBE0/TZVP level with the polarizable continuum model (PCM) for MeOH, were performed as described previously (Lai et al., 2016). ECD spectrum of each conformer was plotted by the program SpecDis (Bruhn et al., 2013) using the dipole-length computed rotational strengths with Gauss curves and exponential half-width (σ) of 0.3 and 0.2 eV, for **1** and **5**, respectively. The equilibrium population of each conformer at 298.15 K was calculated from its relative Gibbs free energies using Boltzmann statistics. The Boltzmann-averaged ECD spectra for (2*S*, 3*R*, 4*R*, 9*R*, 1′*R*, 2′*S*)-**1**, and (1*R*, 2*S*, 4*R*, 1′*R*, 2′*R*)-**5** were generated according to the Boltzmann distributions of the lowest energy conformers for each structure. The calculated ECD spectra were then compared with the experimental one to determine the absolute configuration. The calculated ECD spectra for **1** and **5** were scaled by 0.25 (y-axes) for a better comparison with the experimental data.

### ^13^C NMR Calculation

For the calculations of ^13^C NMR chemical shifts, B3LYP/6-31G(d,p) method was used to optimize the selected conformers. For all optimized structures, vibrational spectra were calculated to ensure that no imaginary frequencies for energy minimum were obtained. NMR calculations were performed at the level of mPW1PW91/6-31G(d,p) with the gauge-independent atomic orbital (GIAO) method (Forsyth and Sebag, 1997). The solvent effect was considered by using the PCM model (CHCl_3_ for **6**). The calculated ^13^C NMR chemical shifts for (*S*)-**6a** and (*S*)-**6b** were analyzed by subtracting the isotopic shifts for TMS calculated with the same method. The ^13^C NMR chemical shifts in each compound were considered as the average value of the same atom in different conformers according to their Boltzmann distributions, using the relative Gibbs free energies as weighting factors. The differences Δδ values were determined by subtracting the experimental chemical shifts (δexp) from the calculated chemical shifts (δcal). The results were evaluated in terms of the average absolute deviation, and maximum absolute deviation.

### Optical Rotation Calculation

The b3lyp/6-31g(d,p)-optimized conformers of (*S*)-**6a** were used to calculate optical rotations (OR). The OR calculations were carried out by means of time-dependent DFT methods at the B3LYP/6-31+G (d,p) level (Mazzeo et al., 2010), with the PCM model for methanol, by Gaussian 09. Boltzmann statistics analysis was employed to calculate the overall OR.

### Phytotoxicity Assay

The major compounds (**12**~**14**, and **16**) were tested for their phytotoxic activities against lettuce (*Lactuca sativa* L. var. *ramose* Hort.) and rice (*Oryza sativa* L. variety Zhonghua11) as described previously (Lu et al., 2015). Glyphosate was used as the positive control. After treatment, the length of the radicle or plumule of each seed was measured. The inhibition ratio (%) was calculated by comparison with the blank control.

### Cytotoxicity Assay

Cytotoxicities of the isolated compounds were tested against human carcinoma cells using the microculture tetrazolium (MTT) assay as described previously (Sun et al., 2017). The tested cell lines included NCI-H460 and A549 (lung carcinoma), BGC-823 (gastric cancer), Daoy (desmoplastic cerebellar medulloblastoma), HepG2 (liver carcinoma), HCT116 (colon cancer), A375 (skin malignant melanoma), MCF-7 (breast cancer), and Capan2 (pancreatic carcinoma). Taxol was used as the positive control. The major compounds (**11**~**14**, **16**) were screened against A375, A549, MCF-7, Capan2, and HCT116 cells, while the minor compounds (i.e., **1**~**10**, **15**, **17**) were tested against NCI-H460, BGC823, Daoy, HepG2, and HCT116 cell lines.

### Antibacterial Assay

The isolated compounds were evaluated for antibacterial activities against six human/plant pathogenic bacteria, including *Bacillus subtilis* ATCC 11562, *Staphylococcus haemolyticus* ATCC 29970, *Ralstonia solanacearum* ATCC11696, *Xanthomonas vesicatoria* ATCC 11633, *Agrobacterium tumefaciens* ATCC 11158, and *Pseudomonas lachrymans* ATCC 11921, by the modified broth dilution colorimetric assay (Shan et al., 2014). The minimum inhibitory concentration (MIC) and half maximum inhibitory concentration (IC_50_) were determined. Streptomycin sulfate was used as the positive control.

### Antifungal Assay

The antifungal activities of compounds **2**, **7**, **10**~**14**, **16**, and **17** were evaluated against the spore germination of the rice blast pathogen *Magnaporthe oryzae* as described previously (Shan et al., 2014). Carbendazim was used as the positive control.

## Results

### Identification of New Compounds

**Figure 1:**
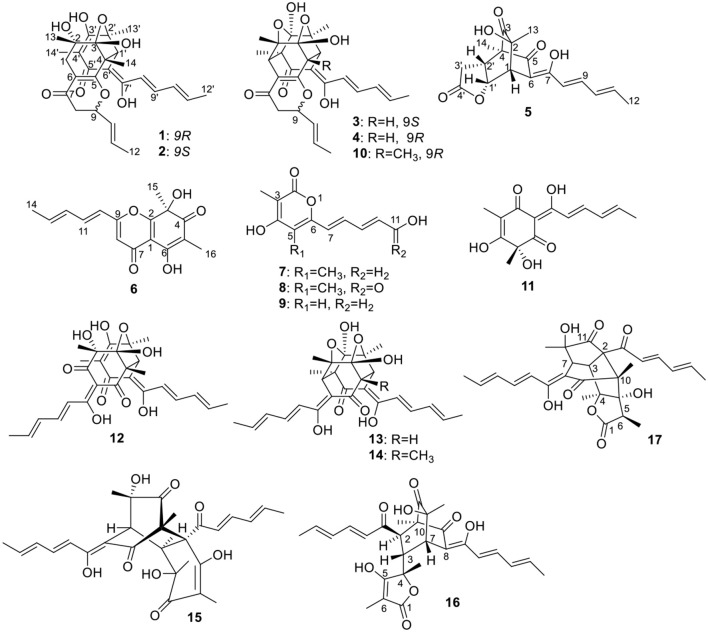
Structures of the isolated sorbicillinoids.

The crude EtOAC extract was repeatedly fractionated over sephadex LH-20, silica gel, and RP-18, and purified by semi-preparative HPLC to give 12 bisorbicillinoids, and five sorbicillinoid-like metabolites (Figure 1).

Compound **1** was isolated as a yellow amorphous powder. It showed a prominent deprotonated peak at *m*/*z* 497.2193 [M-H]^−^ in the negative HRESIMS spectrum, indicating a molecular formula of C_28_H_33_O_8_. Its UV spectrum exhibited maximum absorptions at 220, 271, and 379 nm, which were similar to those of bisvertinols (Trifonov et al., 1986). The IR spectrum indicated the presence of hydroxy (3,420 cm^−1^), ketone (1,714, 1,681 cm^−1^), and double bond (1,646, 1,623 cm^−1^) groups. The ^13^C NMR spectrum displayed a total of 28 carbon resonances, which were assignable to two carbonyls (δ_C_ 193.8, 192.7), 12 sp^2^ hybridized carbons that were incorpated into six double bonds, four quarternary carbons (δ_C_ 107.6, 80.2, 74.5, 57.7), two methines (δ_C_ 79.3, 53.9), two methylenes (δ_C_ 41.1, 33.1), as well as six methyl (δ_C_ 26.6, 22.3, 19.7, 18.8, 18.0, 7.8) groups, by the aid of HMQC spectrum. Accordingly, the ^1^H NMR spectrum showed resonances for six olefinic protons (δ_H_ 5.47~7.16), two methines (δ_H_ 4.26, 3.69), two methylenes (δ_H_ 2.55, 2.44, 2.33, 2.26), and six methyls (δ_H_ 1.86, 1.72, 1.62, 1.46, 1.32, 1.11). These data revealed the bisorbicillinoid nature of **1**. The planar structure of **1** was established by a combined analysis of the 1D, and 2D NMR data.

**Figure 2:**
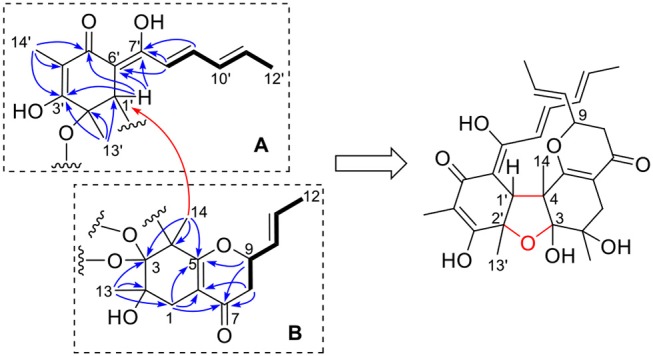
Selected ^1^H-^1^H COSY (bold) and HMBC (H→ C, arrow) correlations of two monomeric units (**A**,**B**) of **1**.

A long spin system was easily recognized by anlysis of the coupling constants and ^1^H-^1^H COSY spectrum, in which cross-peaks were observed between CH_3_-12′ (δ_H_ 1.86, d)/H-11′ (δ_H_ 6.10, dq), H-11′/H-10′ (δ_H_ 6.33, dd), H-10′/H-9′ (δ_H_ 7.16, dd), and H-9′/H-8′ (δ_H_ 6.45, d). The HMBC correlations from H-9′ to C-7′ (δ_C_ 168.3), as well as from H-8′ to C-6′ (δ_C_ 102.8) and C-7′ established an enol-sorbyl chain (box A, Figure 2). Meanwhile, the HMBC correlations from CH_3_-14′ (δ_H_ 1.62, s) to C-3′ (δ_C_ 171.2), C-4′ (δ_C_ 108.8), and C-5′ (δ_C_ 192.7), CH_3_-13′ (δ_H_ 1.46, s) to C-1′ (δ_C_ 53.9), C-2′ (δ_C_ 80.2), and C-3′, and from H-1′ (δ_H_ 3.69, s) to C-5′, C-6′, and C-7′, indicated the presence of a cyclohexenone ring, in which the enol-sorbyl chain was attached to C-6′. Hence, the first monomeric unit (A) was constructed as shown in Figure 2.

The second monomeric unit (box B, Figure 2) was likewise deduced by analysis of the ^1^H-^1^H COSY and HMBC spectra. A spin system starting from H_2_-8 (δ_H_ 2.44, 2.33, each dd), terminating at CH_3_-12 (δ_H_ 1.72, d) was established by inspecting the ^1^H-^1^H COSY spectrum. The HMBC correlations from H-9 (δ_H_ 4.26, ddd), and H_2_-8 to the carbonyl (δ_C_ 193.8, C-7) established a 3-hydroxyhex-4-enoyl side chain. The correlations from CH_3_-13 (δ_H_ 1.11, s) to C-1 (δ_C_ 33.1), C-2 (δ_C_ 74.5), and C-3 (δ_C_ 107.6), from CH_3_-14 (δ_H_ 1.32, s) to C-3, C-4 (δ_C_ 57.7), and C-5 (δ_C_ 172.1), from H_2_-1 (δ_H_ 2.55, 2.26, each d) to C-5, and C-6 (δ_C_ 110.2), allowed the establishment of a cyclohexene ring. Further correlations from H_2_-8 to C-6, H_2_-1 to C-7, indicated that the side chain was attached to C-6 of the cyclohexene ring, while correlation from H-9 to C-5 suggested C-5 and C-9 was connected via an ether bond.

The linkage of the two units was deduced by analysis of the HMBC spectrum. A key HMBC correlation from CH_3_-14 to C-1′ indicated a direct sigma bond between C-1′ and C-4. Considering the molecular formula and the required degrees of unsaturation, one additional ether ring had to be formed. Then, a C-2′-O-C-3 ether was deduced by analysis of the chemical shifts of C-2′ (δ_C_ 80.2) and C-3 (δ_C_ 107.6), which was consistent with those of the reported bisvertinols (Trifonov et al., 1986). Thus, the planar structure of **1** was established as shown in Figure 2.

**Figure 3:**
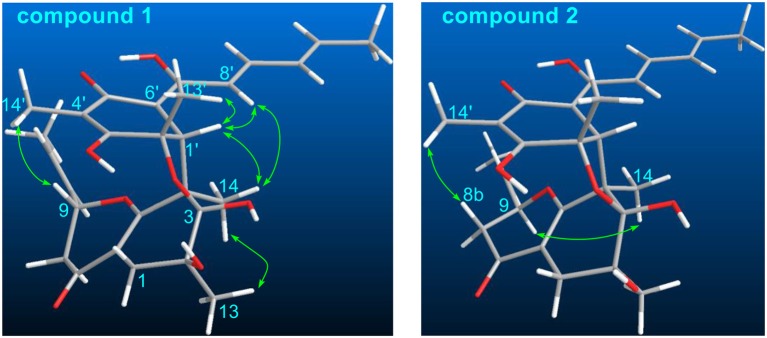
Selected NOESY correlations of **1** and **2**.

The stereochemistry of **1** was determined by analysis of the ^1^H-^1^H coupling constants and the NOESY correlations. The large coupling constants between H-10/H-11 (15.5 Hz), H-8′/H-9′ (14.8 Hz), H-10′/H-11′ (15.1 Hz), defined the *E*-geometry for all these double bonds. The NOESY correlations seen between CH_3_-13/CH_3_-14, CH_3_-14/H-1′, and H-1′/CH_3_-13′ suggested these protons were close in space (Figure 3), while the cross-space correlation between H-9 and CH_3_-14′ was crucial to assign a 9R^*^ configuration.

**Figure 4:**
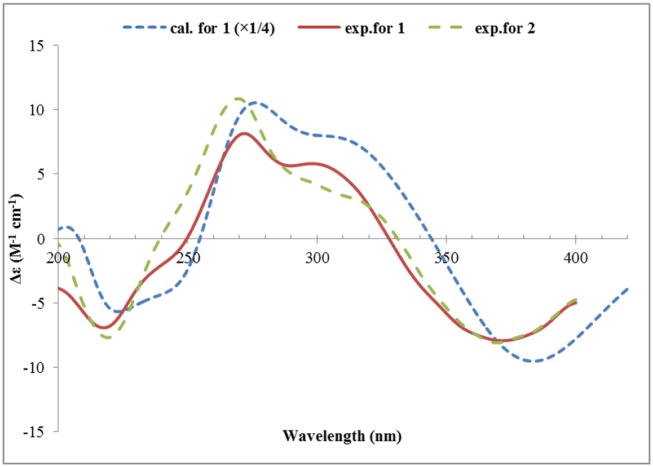
Experimental ECD spectra of **1** and **2**, and the calculated ECD spectrum of (2*S*, 3*R*, 4*R*, 9*R*, 1′*R*, 2′*S*)-**1**.

Structurally, **1** was related to bisvertinol (Andrade et al., 1992), and they only differed at the side chain, in which a 2-propenyl-dihydropyran-4-one in **1** replaced a sorbyl in bisvertinol. The CD spectrum of **1** was similar to that of bisvertinol (Andrade et al., 1992), indicating the absolute configuration should be retained for all the chiral centers except for C-9, though **1** was of smaller magnitudes and hypsochromically shifted. In order to confirm this deduction, the ECD spectrum of **1** was calculated using TDDFT ECD computations, which has been a powerful tool to establish the absolute configuration of natural products (Bringmann et al., 2009). A randomly selected configuration of **1** was used for calculation, which included molecular Merck force field (MMFF) conformational search, geometry optimization using the DFT method at the B3LYP/6-31G(d) level, and ECD calculation of the low-energized conformers (see Table S1 and Figure S1 in the Supplementary Material) at the pbe0/TZVP level with the polarizable continuum model (PCM) in MeOH, successively. The calculated ECD spectrum of (2*S*, 3*R*, 4*R*, 9*R*, 1′*R*, 2′*S*)-**1** matched well with the experimental one (Figure 4), supporting the 2*S*, 3*R*, 4*R*, 9*R*, 1′*R*, 2′*S* configuration of **1**. Compound **1** was designated as ustisorbicillinol A (**1**).

Ustisorbicillinol B (**2**) was isolated as a yellow amorphous powder, and its molecular formula was determined as C_28_H_34_O_8_ by analysis of the HRESIMS spectrum, which was the same as that of **1**. A comparison of the NMR data (Table 1) revealed their great similarities. The notable differences were found at C-8 and C-9, in which C-9 and C-8 of **2** were shifted to downfield (+1.3 and +1.2 ppm, respectively), and H-9 was downfield shifted (+0.30 ppm), while both H_2_-8 were upfield shifted (−0.21, −0.21 ppm), comparing to those of **1**. This implied that **2** differed from **1** only in having a different configuration at C-9. This was confirmed by analysis of the NOESY spectrum, in which cross-peaks were seen between H-9 (δ_H_ 4.56, m) and CH_3_-14 (δ_H_ 1.29, s), while CH_3_-14′ (δ_H_ 1.59, s) showed correlation to H-8b (δ_H_ 2.12, dd), but not to H-9 (Figure 3). Moreover, compound **2** showed similar CD spectrum as that of **1** (Figure 4), indicating their absolute configurations were the same, expect that C-9 had an opposite configuration. Thus, compound **2** was determined as the 9-epimer of **1**.

**Figure 5:**
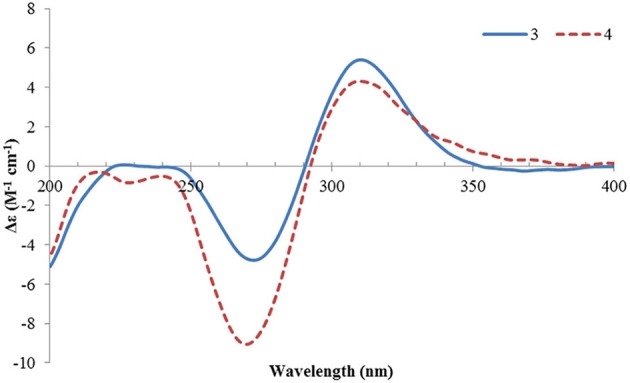
ECD spectra of **3** and **4**.

Ustisorbicillinol C (**3**) had a molecular formula of C_27_H_30_O_8_, as determined by HRESIMS. Its NMR data (Table 1) were similar to those reported for dihydrotrichodimer ether B (Zhai et al., 2016), except that one methyl singlet was missing, while one additional methine replaced one quaternary carbon of the later compound, suggesting that compound **3** was a demethyl derivative of the later when taking into consideration their molecular formulae. This new methine (δ_H_ 3.00, d; δ_C_ 55.7) was positioned at C-4, as it coupled to H-1′ (δ_H_ 3.67, d) (^3^*J* = 12.0 Hz) and showed COSY correlation to H-1′. The HMBC correlation from this methine to C-5 (δ_C_ 170.8), and C-6 (δ_C_ 109.1), as well as the NOESY correlation between this methine and H-1′, unequivocally supported this assgiment. Similar NOE correlations between H-1/CH_3_-14′, H-1/CH_3_-13, H-1′/H-4, H-1′/CH_3_-13′, and H-1′/H-8′, and similar ^3^*J*_H_ values of the vinicial protons of the side chains, indicated a similar relative stereochemistry was retained in **3**. In addition, the CD spectrum of **3** displayed cotton effects at 310 (+5.40), 272 (−4.78) nm (Figure 5), which resembled that of dihydrotrichodimer ether B (Zhai et al., 2016), implying a same absolute configuration for the cage structure. In literature, the absolute configuration of dihydrotrichodimer ether B was determined by comparing its CD spectrum with that of tetrahydrotrichodimer ether, whose configuration had been determined by X-ray diffraction analysis (Zhai et al., 2016). Thus, the structure of ustisorbicillinol C (**3**) was determined as shown in Figure 1.

Ustisorbicillinol D (**4**) was isolated as an isomer of ustisorbicillinol C (**3**), as they shared the same molecular formula. The NMR data of **4** closely resembled those of **3** (Table 1), and the notable differences were found for the hydroxyl-bearing methine (CH-9: δ_H_ 4.38/δ_C_ 81.5 in **4** vs. δ_H_ 4.93/δ_C_ 80.2 in **3**), suggesting they contained the same skeleton but with different configuration for C-9. This was also reflected by the coupling constants found between H-9 and H_2_-8 (^3^*J*_H−9/H−8a_ and ^3^*J*_H−9/H−8b_: 13.4, 3.5 in **4**, vs. 4.8, 8.7 in **3**). Taken together with the CD spectra as shown in Figure 5, ustisorbicillinol D (**4**) was thus determined to be the 9-epimer of ustisorbicillinol C (**3**).

**Figure 6:**
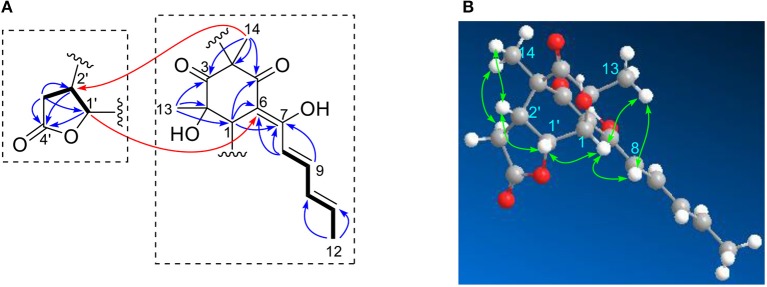
Selected ^1^H-^1^H COSY, and HMBC **(A)**, and NOESY **(B)** correlations of **5**.

Ustisorbicillinol E (**5**), a minor sorbicillinoid, displayed a deprotonated peak at *m*/*z* 331.1201 [M-H]^−^ in the HRESIMS spectrum, suggesting a molecular formula of C_18_H_20_O_6_. Inspection of the ^13^C NMR spectrum disclosed 18 carbon signals (Table 2), which were attributable to two keto groups (δ_C_ 209.2, 194.9), one ester carbonyl (δ_C_ 174.8), six sp^2^-hybridized carbons (δ_C_ 172.3, 144.6, 141.4, 130.8, 117.2, 104.7), two quaternary sp^3^ carbons (δ_C_ 74.2, 62.5), three methines (δ_C_ 76.3, 45.5, 41.4), one methylene (δ_C_ 30.2), and three methyl groups (δ_C_ 19.0, 24.4, 10.4), as aided by an HSQC experiment. Apparently, 14 of those carbons comprised a sorbicillinol-like unit, which included one sorbyl chain (C7~12), two keto (C3 and C5), three non-protonated carbons (C2, C4, and C6), one methine (C1), and two quaternary carbon-attaching methyls (CH_3_-13, CH_3_-14), as revealed by analysis of the ^3^*J*_H_ values, ^1^H-^1^H COSY and HMBC spectra (Figure 6A). Apart from these, four additional carbons remained to be assigned. A spin system involving C-1′~C-3′ was disclosed by inspecting the COSY correlations, while the HMBC correlations from H-1′ (δ_H_ 5.44, dd), H-2′ (δ_H_ 2.89, ddd), and H_2_-3′ (δ_H_ 2.70, 2.24, each dd) to the carbonyl (δ_C_ 174.8, C-4′) indicated the presence of a γ-lactone unit (Figure 6A). The connection between both units was established by the key HMBC correlations from CH_3_-14 (δ_H_ 1.23, s) to C-2′ (δ_C_ 41.4), and from H-1′ to C-6 (δ_C_ 104.7), and the ^1^H-^1^H COSY correlation between H-1′ and H-1 (δ_H_ 3.60, d). Thus, these two units have to be linked via C-2′ /C-4, and C-1′ /C-1 to form a bridge structure (Figure 1).

**Figure 7:**
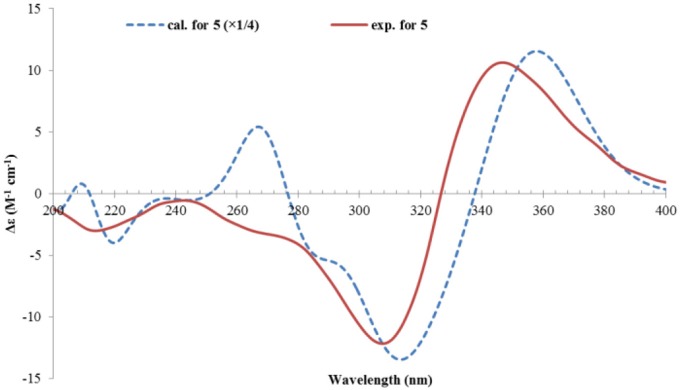
Experimental ECD spectrum of **5**, and the calculated ECD spectrum of (1*R*, 2*S*, 4*R*, 1′*R*, 2′*R*)-**5**.

The relative configuration of **5** was established by analysis of the ^1^H-^1^H coupling constants and NOESY correlations. The large ^3^*J*_H_ values between H-8/H-9 (14.9 Hz), and H-10/H-11 (15.3 Hz) revealed the *E*-configuration of these double bonds. The NOESY correlations between H-1 and H-8 (δ_H_ 6.12, d) indicated the *Z*-configuration of the C6/C7 double bond. The NOESY correlations of H-1 (δ_H_ 3.60, d)/CH_3_-13 (δ_H_ 1.28, s), H-1/H-1′ (δ_H_ 5.44, dd), and H-1′/H-2′ (δ_H_ 2.89, ddd) suggested these protons were close in space, while the observed correlations of H-3′b (δ_H_ 2.24, dd)/CH_3_-14 (δ_H_ 1.23, s), and H-2′/CH_3_-14 were consistent with the equtorial orientation of CH_3_-14, as shown in Figure 6B. The absolute configuration of **5** was determined by TDDFT ECD calculation. MMFF conformational search of randomly selected (1*R*, 2*S*, 4*R*, 1′*R*, 2′*R*)-**5** resulted in 9 conformers within a 21 kJ/mol energy window, which were optimized at the B3LYP/6-31G(d) level *in vacuo* to yield one prodominant conformer (99.1%) (Table S2 and Figure S2). Subsequent ECD calculation with this conformer was performed at the Pbe0/TZVP level with the PCM solvent model for MeOH. The calculated ECD spectrum was in good agreement with the measured spectrum (Figure 7), thus the absolute configuration of **5** was determined to be 1*R*, 2*S*, 4*R*, 1′*R*, 2′*R*. Interestingly, compound 5 was similar to rezishanone A that was isolated from *Penicillium notatum*, and they only differed at C-2′, where a hydroxymethyl group, instead of a proton, was present in rezishanone A (Maskey et al., 2005).

**Figure 8:**
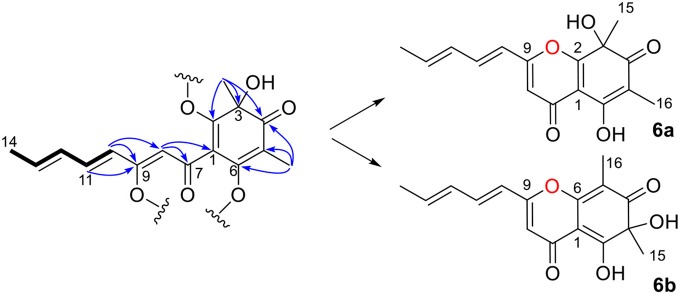
Selected HMBC correlations of **6**, and its two possible structures (**6a** and **6b**).

Ustisorbicillinol F (**6**) was isolated as a homolog of oxosorbicillinol (Abe et al., 2000), and had a molecular formula of C_16_H_16_O_5_, with nine degrees of unsaturation. The NMR data of **6** (Table 3) were similar to those of oxosorbicillinol (Abe et al., 2000), however, two more sp^2^-hybridized carbons (δ_C_ 111.7, CH/δ_H_ 6.31, s; 163.9, C) that comprised a double bond appeared. A detailed interpretation of the 2D NMR data, allowed the placement of this double bond in the sorbyl chain, as key HMBC correlations were observed from H-10 (δ_H_ 6.12, d) to C-9 (δ_C_ 163.9), and C-8 (δ_C_ 111.7), and from H-11 (δ_H_ 7.24) to C-9, as well as from H-8 (δ_H_ 6.31, s) to C-7 (δ_C_ 180.5), and C-1 (δ_C_ 109.7) (Figure 8). To fulfill the required degrees of unsaturation, one additional ring had to be formed via either C9-O-C2 (**6a**) or C9-O-C6 (**6b**) (Figure 8). However, these two possible structures could not be distinguished by NOESY experiments, as no correlations were observed between H-10, CH_3_-15, or CH_3_-16. So ^13^C NMR calculations were performed to determine the structure of **6** (Table S3 and Figure S3). The calculated data for (*S*)-**6a** were in good agreement with the measured values, with average absolute deviation of 2.60 ppm, and maximum absolute deviation of 5.9 ppm, while those of 4.61, 16.9 ppm, respectively, were found for (*S*)-**6b** (Table S4). Therefore, ustisorbicillinol F (**6**) had a planar structure as shown in **6a**. The calculated optical rotation value for (*S*)-**6a** was +60.3 at the b3lyp/6-31+g(d, p) level with PCM model for methanol, while the measured value for **6** was−24 (*c* 0.05, MeOH). Therefore, the absolute configuration for **6** was determined to be *R*.

Ustilopyrone A (**7**), was identified as a 2-pyrone derivative, as inferred from its UV spectrum. The NMR data (Table 4) were similar to those of saturnispol H (Meng et al., 2018), except that an olefinic methyl (δ_C_ 9.6; δ_H_ 2.04, s) of **7** replaced the olefinic proton (H-5) of the latter. This was supported by the HMBC experiment, as correlations were found from 5-CH_3_ (δ_H_ 2.04, s) to C-4 (δ_C_ 167.4), C-5 (δ_C_ 110.5), and C-6 (δ_C_ 153.7).

Additionally, a new analog of ustilopyrone A (**7**), named ustilopyrone B (**8**) was isolated. Its molecular formula was determined as C_12_H_12_O_5_, as revealed by HRESIMS, bearing one more oxygen atom but two less protons than that of **7**. Detailed comparison of the NMR data (Table 4) indicated that they were quite similar, and the differences were found in the side chain, especially at C-11, where a carboxylate group (δ_C_ 170.0) replaced the hydroxymethyl group of **7**. This could explain the differences in molecular formula, as well as the obvious downfield shifts for H-9 (Δδ +0.95 ppm), H-7 (Δδ +0.44 ppm), C-9 (+14.1 ppm), and C-7 (+7.5 ppm), while upfield shift for C-10 (-13.2 ppm). Further evidences were obtained from the HMBC spectrum, as cross-peaks were found from H-9 (δ_H_ 7.44, dd), and H-10 (δ_H_ 6.12, d) to this carboxylate group. Thus, ustilopyrone B (**8**) was determined as the 11-oxidized derivative of **7**.

The other isolated compounds were identified by comparing the spectroscopic data with those published in the literature, and included 5-demethylustilopyrone A (also named saturnispol H, **9**) (Meng et al., 2018) and dihydrotrichodimer ether A (**10**) (Zhai et al., 2016), oxosorbicillinol (**11**) (Abe et al., 2000), bisvertinolone (**12**) (Trifonov et al., 1986), demethyltrichodimerol (**13**) (Abe et al., 1998a), trichodimerol (**14**) (Andrade et al., 1992), bisorbicillinol (**15**) (Abe et al., 1998a), bislongiquinolide (also named trichotetronine) (**16**) (Andrade et al., 1997; Shirota et al., 1997), and bisorbicillinolide (**17**) (Abe et al., 1998b).

### Biological Activity

**Figure 9:**
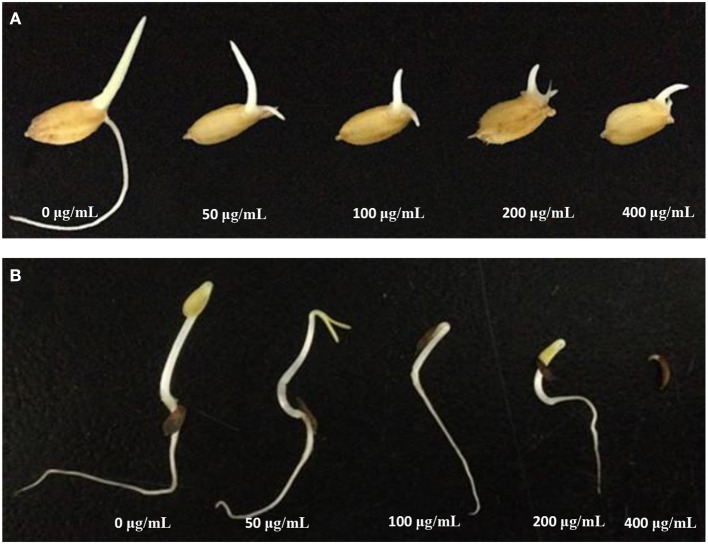
Inhibitory activity of **12** on the radicle and germ elongation of rice seeds **(A)** and lettuce seeds **(B)**.

The major bisorbicillinoids **12**~**14**, and **16** were investigated for their phytotoxic effect against the radicle and germ elongation of rice seeds and lettuce seeds (Table 5). Among them, compound **12** showed the strongest and dose-dependent inhibition in the case of rice seeds (Figure 9A), with 50% inhibitory concentration <50 μg/mL, which was comparable to the positive control glyphosate. At the tested concentration of 200 μg/mL, it showed 98.33% inhibitory rate, which was even better than that of glyphosate (95.80%). The other compounds exhibited much weaker activity, and when tested at 400 μg/mL, only compounds **13** and **16** displayed more than 50% inhibition. With regard to the inhibition of the germ elongation of rice seeds, compound **12** was still the most active one, and it displayed 51.92% inhibitory rate at 200 μg/mL, though weaker than the positive control, whereas the other compounds failed to reach 40% inhibition even at 400 μg/mL. Generally, the germ of rice seeds was less susceptible to the tested substances (including the positive control), comparing to the radicle. However, when tested against the lettuce seeds, different inhibition profiles were observed. When tested at the concentration of 400 μg/mL, the growth of the radicle and germ was completed inhibited by compounds **12**, **13**, and **16**, which was much better than the effect of glyphosate (Table 5). At this concentration, compound **14** displayed >50% inhibition against the elongation of radicle and germ, though it was the least active one. Compound **12** also exhibited the strongest inhibition amongst the tested compounds (Figure 9B). It was worth noting that the germ was equal or slightly more sensitive to the tested substances at lower concentration (<200 μg/mL) than the radicle, in general, while at higher concentrations this trend was reversed.

**Table 5:** Phytotoxicity of the isolated compounds against the radicle and germ elongation of rice and lettuce seeds.

**Compound**	**Concentration** **(μg/mL)**	**Rice seeds, inhibitory rate (%)** ^**a**^		**Lettuce seeds, inhibitory rate (%)** ^**a**^	
		**Radical elongation**	**Germ elongation**	**Radical elongation**	**Germ elongation**
**12**	50	65.37 ± 3.54c	20.33 ± 6.74c	8.31 ± 1.06ij	14.73 ± 3.07hi
	100	88.42 ± 1.79b	29.81 ± 7.01c	16.06 ± 1.77fg	15.68 ± 1.35hi
	200	98.33 ± 3.33a	51.92 ± 8.85b	58.30 ± 3.91e	33.58 ± 3.32d
	400	98.86 ± 1.41a	64.84 ± 6.65a	100.00 ± 0.00a	100.00 ± 0.00a
**13**	50	9.79 ± 5.90c	14.56 ± 7.93a	7.82 ± 1.53j	8.55 ± 2.02j
	100	23.49 ± 9.38b	12.91 ± 3.52a	11.74 ± 2.82hi	13.64 ± 2.64i
	200	35.07 ± 8.80b	14.70 ± 5.63a	18.85 ± 2.97f	16.85 ± 3.35ghi
	400	68.47 ± 5.31a	18.41 ± 7.18a	100.00 ± 0.00a	100.00 ± 0.00a
**14**	50	7.49 ± 3.66b	3.57 ± 7.14c	3.95 ± 0.57k	6.00 ± 0.27j
	100	5.37 ± 5.35b	16.62 ± 3.25b	11.72 ± 2.59hi	21.06 ± 2.73fg
	200	4.38 ± 7.19b	18.41 ± 6.80b	15.95 ± 2.47fg	26.87 ± 4.15e
	400	45.89 ± 6.87a	36.95 ± 5.06a	60.60 ± 1.31e	50.87 ± 4.34c
**16**	50	18.25 ± 6.04b	12.64 ± 10.63bc	9.96 ± 1.28hij	8.97 ± 2.04*j*
	100	23.24 ± 8.05b	7.55 ± 6.29b	13.03 ± 1.92gh	17.21 ± 3.28fghi
	200	43.83 ± 1.28a	22.25 ± 8.62ab	18.86 ± 2.08f	21.31 ± 3.68f
	400	51.49 ± 5.09a	31.46 ± 3.02a	100.00 ± 0.00a	100.00 ± 0.00a
Glyphosate^b^	50	84.51 ± 3.29c	59.34 ± 8.94c	75.92 ± 2.80d	18.51 ± 0.77*fgh*
	100	93.93 ± 2.24b	74.18 ± 3.17ab	77.41 ± 3.59cd	27.63 ± 0.95e
	200	95.80 ± 1.30b	72.12 ± 4.43b	80.23 ± 1.77bc	28.80 ± 2.43e
	400	99.62 ± 0.76a	81.46 ± 4.36a	82.97 ± 1.53b	56.81 ± 3.65b

a The values were expressed as means ± SD (n = 3). Different letters indicated significant differences among treatments in each column at p ≤ 0.05.

b *Positive control*.

When compared the inhibitory activity of each tested compound at each tested concentration against both type of seeds, a general potency of **12**>**16**≥**13**>**14** was found. More structures should be evaluated in order to gain a better understanding of the structure-activity relationship.

The isolated compounds were also evaluated for their cytotoxicities against nine human carcinoma cell lines including NCI-H460, BGC823, Daoy, HepG2, HCT116, A375, A549, MCF-7, and Capan2 (Table 6). Compounds **10**, **12**, **13**, and **14** showed moderate cytotoxicities against the tested cell lines, whereas the other isolated compounds were inactive (IC_50_ > 50 μM). Among the active compounds, compound **10** exhibited the strongest inhibition with IC_50_ in the range of 8.83~38.2 μM, though less potent than the positive control. It was interesting that all the active compounds were bisorbicillinols with at least one ether bridge. The methyl group at C-14 might positively correlate to the cytotoxicity, as in the case of **10** vs. **4**. However, the structure-activity relationship remained unclear.

**Table 6:** Cytotoxicity of the isolated compounds (IC_50_, μM).

**Compound^**a**^**	**NCI-H460**	**BGC823**	**Daoy**	**HepG2**	**HCT116**	**A375**	**A549**	**MCF-7**	**Capan2**
**10**	8.83	38.2	21.2	25.8	12.9	–	–	–	–
**12**	–	–	–	–	28.7	52.4	25.4	38.3	>50.0
**13**	–	–	–	–	>50.0	41.3	45.0	74.7	>50.0
**14**	–	–	–	–	31.7	45.0	60.5	48.8	>50.0
Taxol^b^	<0.008	<0.008	0.00504	0.0752	0.0019	0.0220	0.0232	<0.008	0.0167

a The other tested compounds were inactive (IC_50_ > 50.0 μM).

b *Positive control*.

The antibacterial activities of the isolated compounds were also tested (Table 7). Compounds **2**, and **10**~**13** were active against the tested pathogenic bacteria including *A. tumefaciens, B. subtilis, P. lachrymans, R. solanacearum, S. haemolyticus*, and *X. vesicatoria*, with MIC values in the range of 4~128 μg/mL, whereas the other compounds were inactive (MIC > 128 μg/mL). Compound **12** was the most potent one, with MIC values ≤8 μg/mL, which were comparable to those of the positive control (streptomycin sulfate), and this was followed by compound **13** (MICs ≤ 32 μg/mL). It is interesting that except the monomeric compound, oxosorbicillinol (**11**), the other active compounds were bisorbicillinols (**2**, **10**, **12**, **13**) with one or two ether bridges.

**Table 7:** Antibacterial activity of the isolated compounds.

**Bacterium**	**MIC/IC_**50**_** **(μg/mL)**	**Compound**^**a**^					
		**2**	**10**	**11**	**12**	**13**	**Streptomycin sulfate^**b**^**
*A. tumefaciens*	MIC	64	64	128	8	32	5
	IC_50_	35.43	75.68	38.76	3.36	22.72	1.41
*B. subtilis*	MIC	64	128	128	8	32	5
	IC_50_	41.92	67.26	43.23	4.01	24.23	1.37
*P. lachrymans*	MIC	32	64	64	8	32	7.5
	IC_50_	37.15	73.55	16.71	3.87	12.87	2.34
*R. solanacearum*	MIC	64	64	64	4	24	5
	IC_50_	80.66	22.29	29.53	3.67	9.58	1.21
*S. haemolyticus*	MIC	64	128	128	4	32	5
	IC_50_	14.78	35.20	49.80	2.02	15.34	1.4
*X. vesicatoria*	MIC	64	64	64	8	24	10
	IC_50_	25.66	41.41	19.59	4.73	10.13	2.59

a *The other isolated compounds were inactive with MIC > 128 μg/mL*.

b *Positive control*.

Additionally, compounds **2**, **7**, **10**~**14**, **16**, and **17** were tested for inhibition against the germination of the spores of rice blast fungus *M. oryzae*. Compounds **11**~**13** displayed moderate effect with IC_50_ values of 40.88, 36.70, and 55.99 μg/mL, respectively, compared to the positive control carbendazim (IC_50_ 6.86 μg/mL). This implied that the bisorbicillinoids **12** and **13** were more potent than the monomeric compound **11**, when taking into consideration of their molar units. The other tested compounds did not exhibited strong inhibition (IC_50_ > 200 μg/mL). No clear structure-activity relationship could be drawn.

## Discussion

In the present study, we isolated 17 sorbicillinoids including eight new compounds from the pathogenic fungus *U. virens*. These isolated compounds could be classified as monomeric sorbicillinoid (**11**), dimeric sorbicillinoids (**1**~**5**, **10**, **12**~**17**), and related compounds (**6**~**9**).

**Figure 10:**
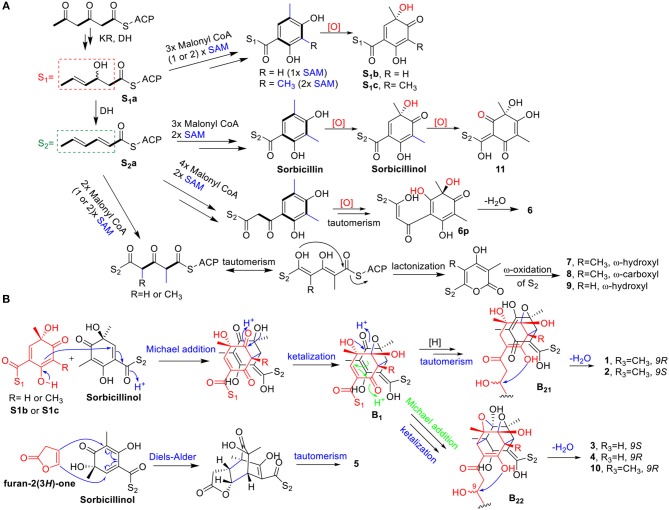
Proposed biosynthetic route for the monomeric sorbicillinoid and related metabolites (**6**~**9**, **11**) **(A)**, and the dimeric sorbicillinoids (**1**~**5**, **10**) **(B)**.

The biosynthesis of sorbicillinol has been reported in *Penicillium chrysogenum* (Fahad et al., 2014) that involved SorA, a highly-reducing iterative PKS (HRiPKS), and SorB, a non-reducing iterative PKS (NRiPKS). SorA combined three acetate units, and modified the forming triketide with the ketoacyl reductase and dehydratase domains to produce the sorbyl-containing thioester (**S**_**2**_**a**), which was passed to SorB to appendix three further acetate units and two methyl groups, resulting in the formation of sorbicillin. Further oxidation of sorbicillin generated sorbicillinol, and oxosorbicillinol (**11**), by the FAD-dependent monooxygenase SorC, and oxidoreductase SorD, respectively (Figure 10A) (Guzmán-Chávez et al., 2017). Similarly, the biosynthesis of the analogs of sorbicillinol, **S**_**1**_**b**, and **S**_**1**_**c**, with a 3-hydroxy-4-hexenoyl side chain, were proposed. It was speculated that there was a 3-hydroxy-4-hexenoyl thioester intermediate (**S**_**1**_**a**) before the formation of the sorbyl thioester (**S**_**2**_**a**), though this has not been reported yet. The addition of three malonyl CoA, and one or two methyl group from *S*-adenosylmethionine (SAM) could produce the sorbicillin analogs with S_1_-type of side chain, which were then oxidized to give the sorbicillinol analogs, 9-hydroxydihydrosorbicillinol (**S**_**1**_**c**), and 3-demethyl-9-hydroxydihydrosorbicillinol (**S**_**1**_**b**). Likewise, the biosynthesis of sorbicillinol related compound, **6**, was envisaged to involve one more acetate unit when appended to **S**_**2**_**a**, resulting in the formation of a sorbicillin analog with a 8C side chain. Subsequent oxidation, and keto-tautomerism of this analog would give the precursor **6p**, which could lose one molecule of water to yield the C_2_-O-C_9_ cyclic ether (i.e., **6**) (Figure 10A). With regard to the biosynthesis of 2-pyrones, it was hypothesized that two acetate units, and one or two methyl groups should add to the thioester **S**_**2**_**a**, following by keto-enol tautomerism and lactonization to give the 2-pyrone products with the S_2_-type side chain. The oxidation at the end of this chain (ω-oxidation) would give rise to **7**~**9** as depicted in Figure 10A. Taken together the above mentioned pathways, the PKS, especially the SorB-like NRiPKS, of this fungus showed a remarkable plasticity, as it added 2~4 units of malonyl CoA to the triketide produced by the HRiPKS, resulting in a different type of products.

The biosynthetic pathway for bisorbicillinols **1**~**5**, and **10** was proposed in Figure 10B. Sorbicillinol was reported to be a key intermediate for the biosynthesis of bisorbicillinols (Harned and Volp, 2011; Meng et al., 2016). The Michael addition of sorbicillinol and its analog **S**_**1**_**b** or **S**_**1**_**c**, followed by ketalization, could produce the intermediates as shown in **B**_**1**_. These intermediates could be hydrogenated and keto-enol tautomerized to form another intermediates (**B**_**21**_) that underwent dehydration in the side chain of S_1_ to yield **1** and **2**. On the other hand, intramolecular Michael addition and ketalization of **B**_**1**_ could generate the intermediates (**B**_**22**_), which were dehydrated to form the bisorbicillinols **3**, **4**, and **10**. Whereas, the formation of **5**, a heterodimer, should involve the Diels-Alder reaction of sorbicillinol and furan-2(3*H*)-one, followed by a keto-enol tautomerism step.

Compounds **1**-**4** and **10**, featuring a dihydro-γ-pyrone fused cyclohexene ring, were interesting members of the bisorbicillinoids. Previously, this type of structures has only been reported once, and included tetrahydrotrichodimer ether, and dihydrotrichodimer ethers A and B (Zhai et al., 2016). It is of interest that both epimers at C-9 were co-isolated in this study, as well as in the literature (Zhai et al., 2016). The possibility that an acid-mediated epimerism could occur can be ruled out, as these compounds were detectable in the crude extract. In addition, incubation of **2** in 80%MeOH/H_2_O (containing 0.02% TFA) at RT for 1 week, the same condition employed for separation, did not lead to the production of any decomposed product.

Sorbicillinoids were reported to have diverse biological activities, such as cytotoxic, antimicrobial, and antioxidant activity (Harned and Volp, 2011; Meng et al., 2016). In this study, the isolated compounds were tested for their cytotoxic, antibacterial, and antifungal activities. The bisorbicillinoids **10**, and **12~14** showed moderate cytotoxicities against the tested cell lines. These cytotoxic compounds (except **14**), along with compounds **2** and **11**, displayed antibacterial activity. In addition, the bisorbicillinoids **12** and **13**, as well as the monomer **11** displayed moderate antifungal activities. Among the antimicrobial compounds, **12** was the most effective. Meanwhile, the isolated compounds were screened for their phytotoxicities, and the results revealed that bisorbicillinoids were potent phytotoxins. Such activity has not been reported for sorbicillinoids so far. Whether these phytotoxins play a role in the pathogenesis of *U. virens* are yet to be discovered.

## Conclusion

In conclusion, 17 sorbicillinoids were isolated from the fermentation of *U. virens*, which included five new bisorbicillinols, ustisorbicillinols A~E (**1**~**5**), and one new oxosorbicillinol-related compound, ustisorbicillinol F (**6**), together with two new 2-pyrones (**7** and **8**). The structures of the new compounds were elucidated by a combined analysis of the HRESIMS, NMR, and CD spectra, and by quantum chemical calculations of ECD, ^13^C NMR, and OR. Their biosynthetic pathways were proposed. The isolated compounds were evaluated for their phytotoxic, cytotoxic, antibacterial, and antifungal activities. The major bisorbicillinoids **12**~**14**, and **16** were phytotoxic against radicle or germ growth of rice and lettuce seeds, with **12** displaying the strongest inhibition. Compound **12** could be a promising lead for the development of natural herbicides. The phytotoxicity of sorbicillinoids has not been reported previously. Compounds **10**, and **12~14** showed moderate cytotoxicities against the tested cell lines with IC_50_s of 8.83~74.7 μM. Compounds **2**, and **10**~**13** were active against the tested bacteria (MICs 4~128 μg/mL). Meanwhile, compounds **11**~**13** displayed moderate antifungal activities.

## Data Availability

The raw data supporting the conclusions of this manuscript will be made available by the authors, without undue reservation, to any qualified researcher.

## Author Contributions

LZ, DL, and JM conceived and designed the experiments. JM was responsible for the isolation of compounds. DL and JM elucidated the structures. JD tested cytotoxicity of the compounds. JM, GG, PD, XZ, and WW performed the experiments of phytotoxic and antimicrobial activities. DL, LZ, and JM interpreted the data and wrote the paper. YL and JD revised the manuscript. All authors read and approved the final manuscript.

### Conflict of Interest Statement

The authors declare that the research was conducted in the absence of any commercial or financial relationships that could be construed as a potential conflict of interest.
