# Supplementary material for: Sorbicillinoids From the Fungus Ustilaginoidea virens and Their Phytotoxic, Cytotoxic, and Antimicrobial Activities
Source: Front Chem. 2019 Jun 12;7:435. doi: 10.3389/fchem.2019.00435 (PMC6582230; doi:10.3389/fchem.2019.00435)
Supplement: Supplementary file 1 [file Data_Sheet_1.PDF]

Supporting Information

Sorbicillinoids from the Fungus *Ustilaginoidea virens* and Their Phytotoxic, Cytotoxic, and Antimicrobial Activities

Jiajia Meng[1], Gan Gu[1], Pengqin Dang[1], Xuping Zhang[1], Weixuan Wang[1], Jungui Dai[2], Yang Liu[3], Daowan Lai[1] and Ligang Zhou[1]**

[1] Department of Plant Pathology, College of Plant Protection, China Agricultural University, Beijing 100193, China, [2] State Key Laboratory of Bioactive Substance and Function of Natural Medicines, Institute of Materia Medica, Chinese Academy of Medical Science & Peking Union Medical College, Beijing 100050, China, [3] Institute of Food Science and Technology, Chinese Academy of Agricultural Sciences, Beijing 100193, China

[*] **Corresponding Authors**

Daowan Lai: dwlai@cau.edu.cn; Ligang Zhou: lgzhou@cau.edu.cn

Contents

Table S1. Important thermodynamic parameters (a.u.) and Boltzmann distributions of the predominant conformers (>1%) of **1** S4
Figure S1. Optimized geometries of predominant conformers for **1** at the B3LYP/6-31G(d) level in the gas phase S4
Table S2. Important thermodynamic parameters (a.u.) and Boltzmann distributions of the predominant conformers (>1%) of **5** S5
Figure S2. Optimized geometries of predominant conformer for **5** at the B3LYP/6-31G(d) level in the gas phase S5
Table S3. Important thermodynamic parameters (a.u.) and Boltzmann distributions of the predominant conformers (>1%) of (*S*)-**6a** and (*S*)-**6b** S6
Figure S3. Optimized geometries of predominant conformers for (*S*)-**6a** and (*S*)-**6b** at the B3LYP/6-31G(d, p) level in the gas phase S6
Table S4. ^{13}C NMR calculations of **6a** and **6b** S7
NMR, HRESIMS, IR, and CD spectra of 1 .... S8
^{1}H NMR spectrum of **1** (CD_3OD, 400MHz) S8
^{13}C NMR spectrum of **1** (CD_3OD, 100MHz) S8
HMQC spectrum of **1** S9
HMBC spectrum of **1** S9
NOESY spectrum of **1** S10
HRESIMS spectrum of **1** S10
IR spectrum of **1** S11
CD spectrum of **1** S11
NMR, HRESIMS, IR, and CD spectra of 2 .... S12
^{1}H NMR spectrum of **2** (CD_3OD, 400MHz) S12
^{13}C NMR spectrum of **2** (CD_3OD, 100MHz) S12
HMQC spectrum of **2** S13
HMBC spectrum of **2** S13
NOESY spectrum of **2** S14
HRESIMS spectrum of **2** S14
IR spectrum of **2** S15
CD spectrum of **2** S15
NMR, HRESIMS, IR, and CD spectra of 3 .... S16
^{1}H NMR spectrum of **3** (CD_3COCD_3, 600MHz) S16
^{13}C NMR spectrum of **3** (CD_3COCD_3, 150MHz) S16
HSQC spectrum of **3** S17
HMBC spectrum of **3** S17
NOESY spectrum of **3** S18
HRESIMS spectrum of **3** S18
IR spectrum of **3** S19
CD spectrum of **3** S19
NMR, HRESIMS, IR, and CD spectra of 4 .... S20
^{1}H NMR spectrum of **4** (CD_3COCD_3, 600MHz) S20

^{13}C NMR spectrum of **4** (CD_3COCD_3, 150MHz) S20
HSQC spectrum of **4** S21
HMBC spectrum of **4** S21
NOESY spectrum of **4** S22
HRESIMS spectrum of **4** S22
IR spectrum of **4** S23
CD spectrum of **4** S23
NMR, HRESIMS, IR, and CD spectra of 5 .... S24
^{1}H NMR spectrum of **5** ($CDCl_3$, 600MHz) S24
^{13}C NMR spectrum of **5** ($CDCl_3$, 150MHz) S24
HSQC spectrum of **5** S25
HMBC spectrum of **5** S25
NOESY spectrum of **5** S26
HRESIMS spectrum of **5** S26
IR spectrum of **5** S27
CD spectrum of **5** S27
NMR, HRESIMS, and IR spectra of 6 .... S28
^{1}H NMR spectrum of **6** ($CDCl_3$, 600MHz) S28
^{13}C NMR spectrum of **6** ($CDCl_3$, 150MHz) S28
HMBC spectrum of **6** S29
NOESY spectrum of **6** S29
HRESIMS spectrum of **6** S30
IR spectrum of **6** S30
NMR, HRESIMS, and IR spectra of 7 .... S31
^{1}H NMR spectrum of **7** (CD_3OD, 400MHz) S31
^{13}C NMR spectrum of **7** (CD_3OD, 100MHz) S31
HMBC spectrum of **7** S32
HRESIMS spectrum of **7** S32
IR spectrum of **7** S33
NMR, HRESIMS, and IR spectra of 8 .... S34
^{1}H NMR spectrum of **8** (CD_3OD, 400MHz) S34
^{13}C NMR spectrum of **8** (CD_3OD, 100MHz) S34
HMBC spectrum of **8** S35
HRESIMS spectrum of **8** S35
IR spectrum of **8** S36
NMR, HRESIMS, and IR spectra of 9 .... S37
^{1}H NMR spectrum of **9** (CD_3OD, 400MHz) S37
^{13}C NMR spectrum of **9** (CD_3OD, 100MHz) S37
HRESIMS spectrum of **9** S38
IR spectrum of **9** S38

Table S1. Important thermodynamic parameters (a.u.) and Boltzmann distributions of the predominant conformers (>1%) of **1** [a]

Conformer [b]	E	E′=E+ZPE	H	G	% [c]
**1b**	-1688.574830	-1688.610499	-1688.573886	-1688.675895	6.73
**1c**	-1688.576399	-1688.612247	-1688.575455	-1688.678220	79.15
**1d**	-1688.574271	-1688.610054	-1688.573327	-1688.676032	7.79
**1g**	-1688.573148	-1688.608834	-1688.572204	-1688.674681	1.86
**1k**	-1688.573265	-1688.609191	-1688.572321	-1688.675053	2.76

[a] E, E′, H, G: total energy, total energy with zero point energy (ZPE), enthalpy and Gibbs free energy in the gas phase at B3LYP/6-31G(d) level.

[b] The conformers with populations less than 1% calculated from their relative free energies were not shown.

[c] %: Boltzmann distributions, using the relative Gibbs free energies as weighting factors.

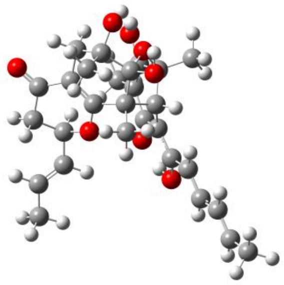

1b

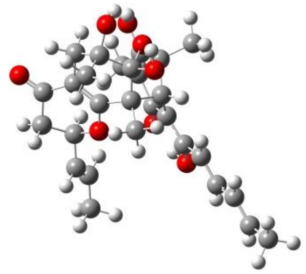

1c

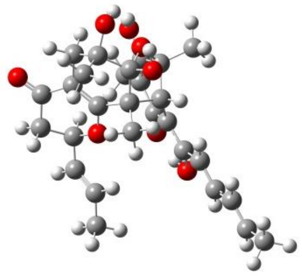

1d

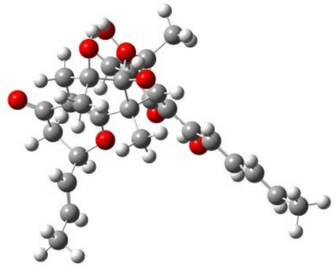

1g

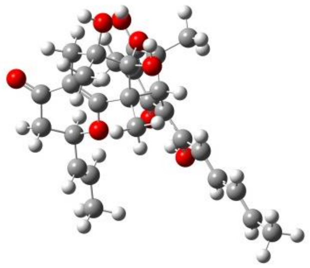

1k

Figure S1. Optimized geometries of predominant conformers for **1** at the B3LYP/6-31G(d) level in the gas phase

Table S2. Important thermodynamic parameters (a.u.) and Boltzmann distributions of the predominant conformers (>1%) of **5** [a]

Conformer [b]	E	E′=E+ZPE	H	G	% [c]
**5a**	-1148.907744	-1148.930324	-1148.906800	-1148.982093	99.11012223

[a] E, E′, H, G: total energy, total energy with zero point energy (ZPE), enthalpy and Gibbs free energy in the gas phase at B3LYP/6-31G(d) level.

[b] The conformers with populations less than 1% calculated from their relative free energies were not shown.

[c] %: Boltzmann distributions, using the relative Gibbs free energies as weighting factors.

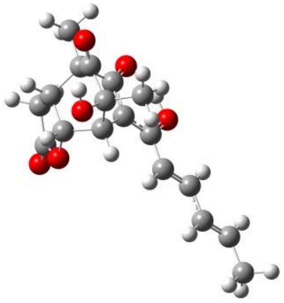

5a

Figure S2. Optimized geometries of predominant conformer for **5** at the B3LYP/6-31G(d) level in the gas phase

Table S3. Important thermodynamic parameters (a.u.) and Boltzmann distributions of the predominant conformers (>1%) of (*S*)-**6a** and (*S*)-**6b** [a]

Input structure	Conformer [b]	E	E′=E+ZPE	H	G	% [c]
(*S*)-**6a**	**6a1**	-995.144981	-995.165202	-995.144037	-995.214443	90.72
	**6a3**	-995.141688	-995.162077	-995.140744	-995.212245	8.83
(*S*)-**6b**	**6b1**	-995.140753	-995.160918	-995.139809	-995.209673	91.31
	**6b3**	-995.137359	-995.157718	-995.136415	-995.207334	7.66

[a] E, E′, H, G: total energy, total energy with zero point energy (ZPE), enthalpy and Gibbs free energy in the gas phase at B3LYP/6-31G(d,p) level.

[b] The conformers with populations less than 1% calculated from their relative free energies were not shown.

[c] %: Boltzmann distributions, using the relative Gibbs free energies as weighting factors.

(*S*)-**6a**:

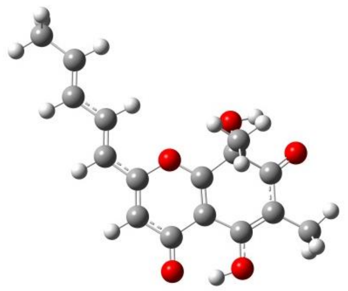

6a1

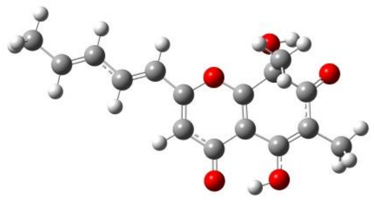

6a3

(*S*)-6b:

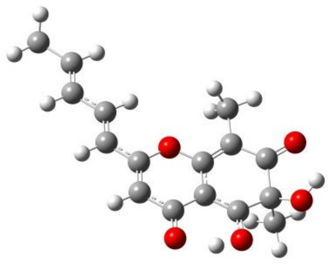

6b1

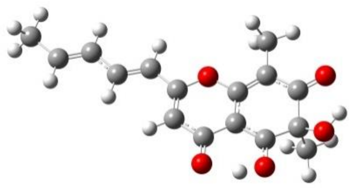

6b3

Figure S3. Optimized geometries of predominant conformers for (*S*)-**6a** and (*S*)-**6b** at the B3LYP/6-31G(d, p) level in the gas phase

Table S4. ^{13}C NMR calculations of **6a** and **6b**

No.	*δ*exp	*δ*cal (**6a**)	Δ*δ* [a]	*δ*cal (**6b**)	Δ*δ*
1	109.7	110.6	0.9	101.5	-8.2
2	169.2	170.2	1.0	186.1	16.9
3	73.7	77.7	4.0	80.8	7.1
4	196.9	192.4	-4.5	194.4	-2.5
5	106.1	104.6	-1.5	102.3	-3.8
6	165.5	166.1	0.6	162.1	-3.4
7	180.5	174.6	-5.9	177.5	-3.0
8	111.7	111.4	-0.3	105.4	-6.3
9	163.9	160.9	-3.0	161.6	-2.3
10	118.6	116.6	-2.0	118.7	0.1
11	140.1	139.4	-0.7	139.8	-0.3
12	130.1	127.6	-2.5	128.2	-1.9
13	140.6	144.6	4.0	144.7	4.1
14	18.9	22.4	3.5	23.5	4.6
15	30.0	33.3	3.3	34.5	4.5
16	6.8	10.7	3.9	11.5	4.7
Average absolute deviation			2.60		4.61
Maximum absolute deviation			5.9		16.9

[a] Δ*δ* =*δ*cal-*δ*exp

NMR, HRESIMS, IR, and CD spectra of 1

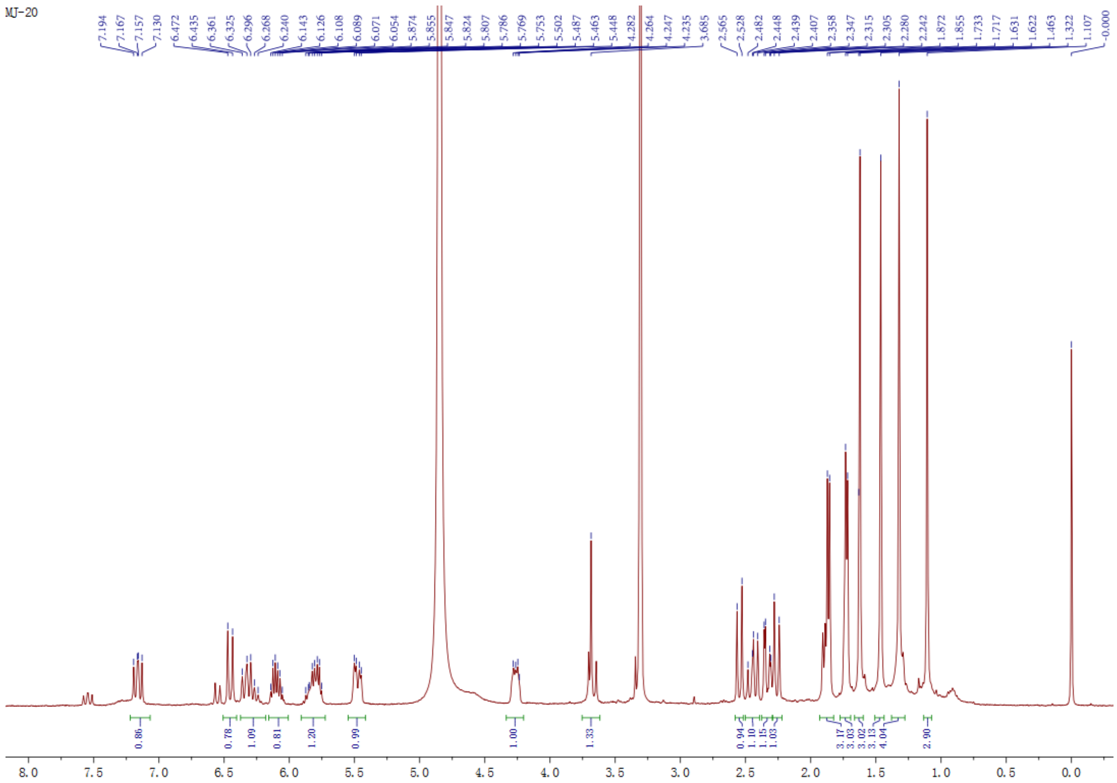

^{1}H NMR spectrum of **1** (CD_3OD, 400MHz)

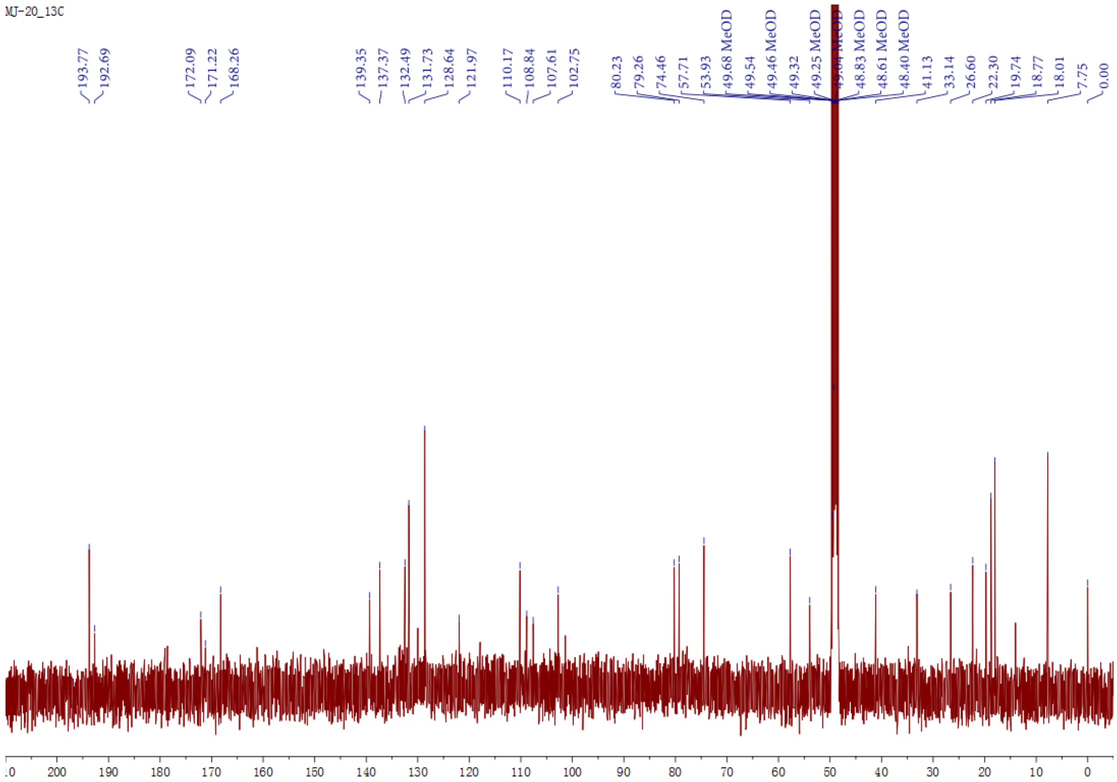

^{13}C NMR spectrum of **1** (CD_3OD, 100MHz)

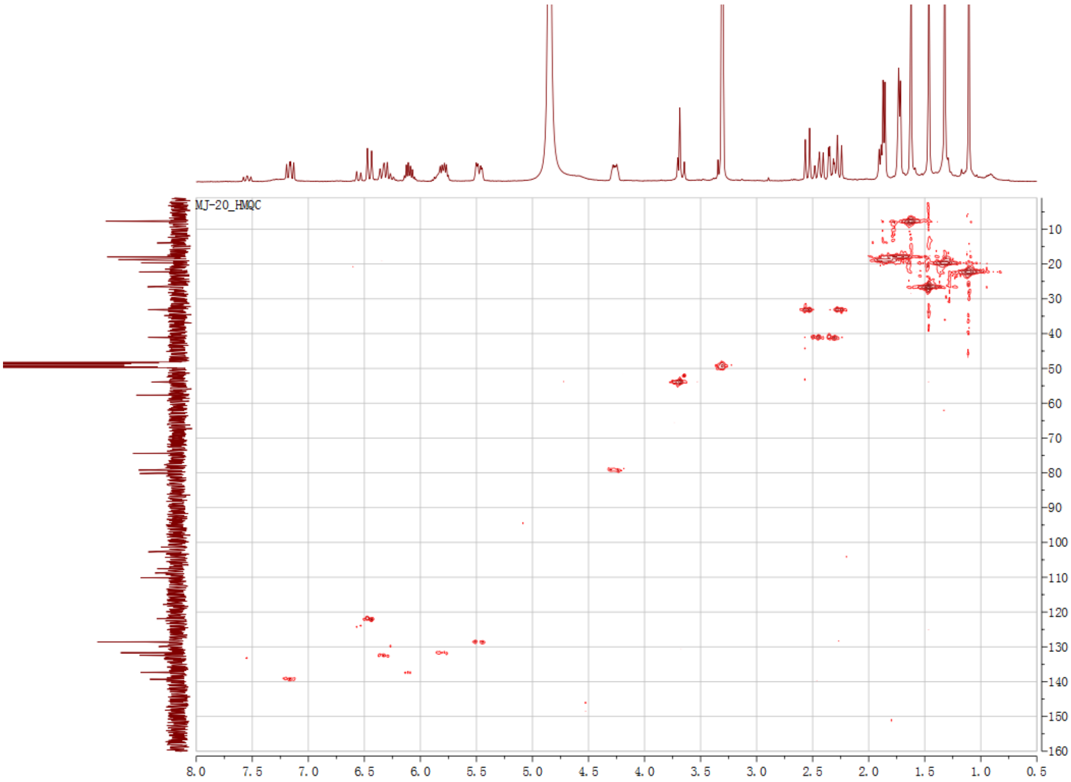

HMQC spectrum of **1**

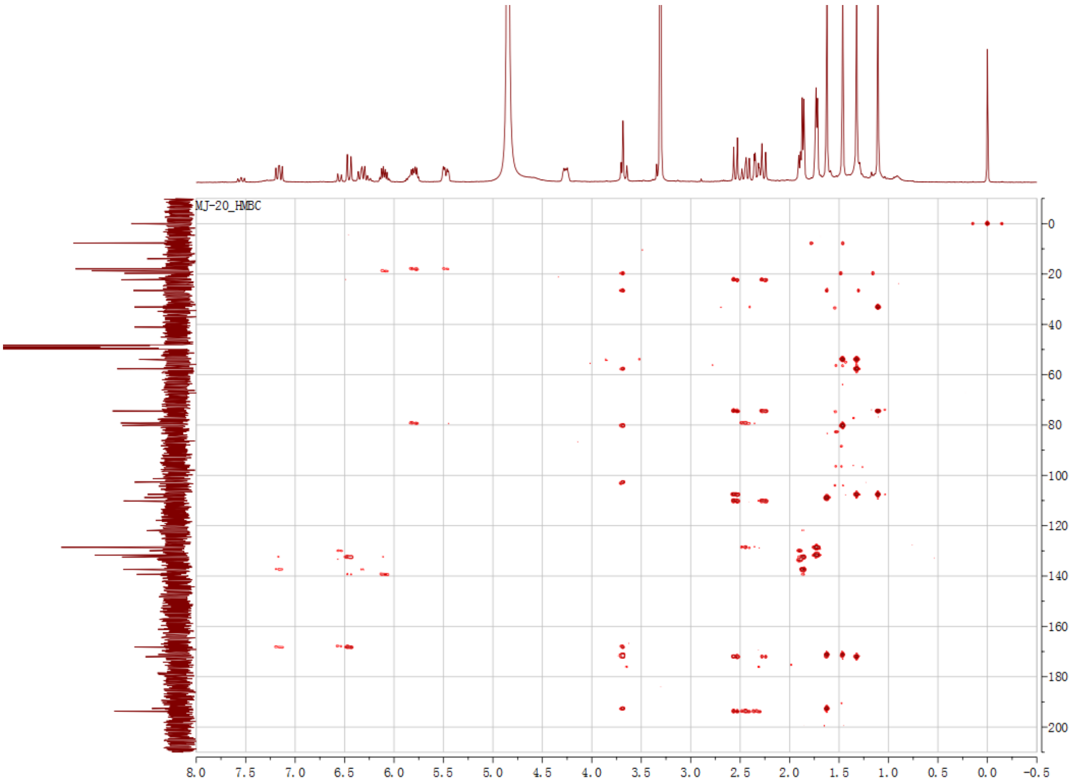

HMBC spectrum of **1**

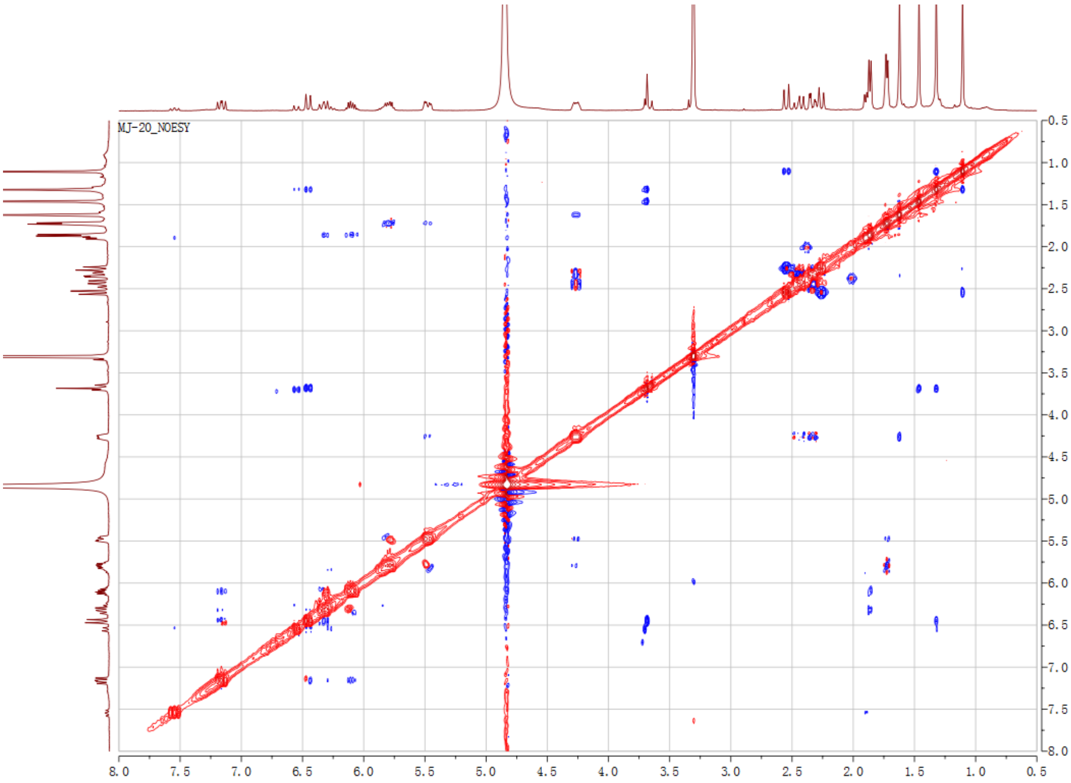

NOESY spectrum of **1**

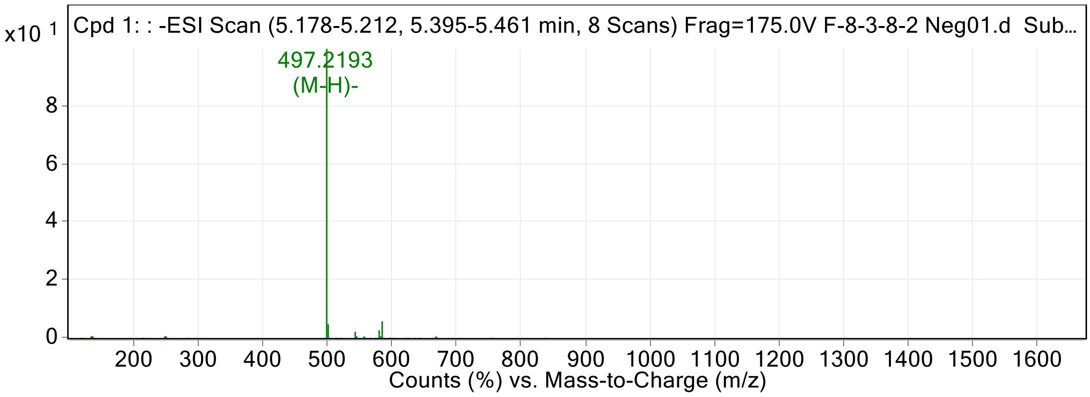

m/z	*Calc m/z*	Diff(ppm)	z	Abund	Formula	Ion
497.2193	497.2181	2.36	-1	199284.1	$C_{28}H_{33}O_8$	(M-H)-

HRESIMS spectrum of **1**

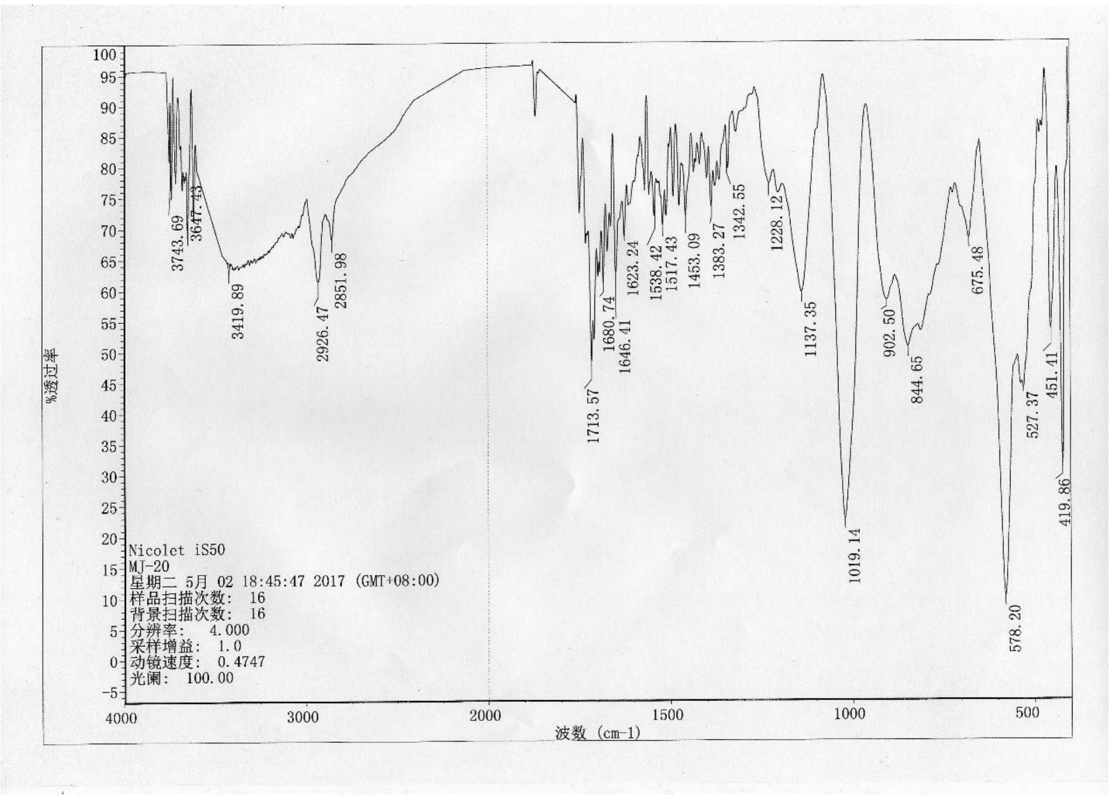

IR spectrum of **1**

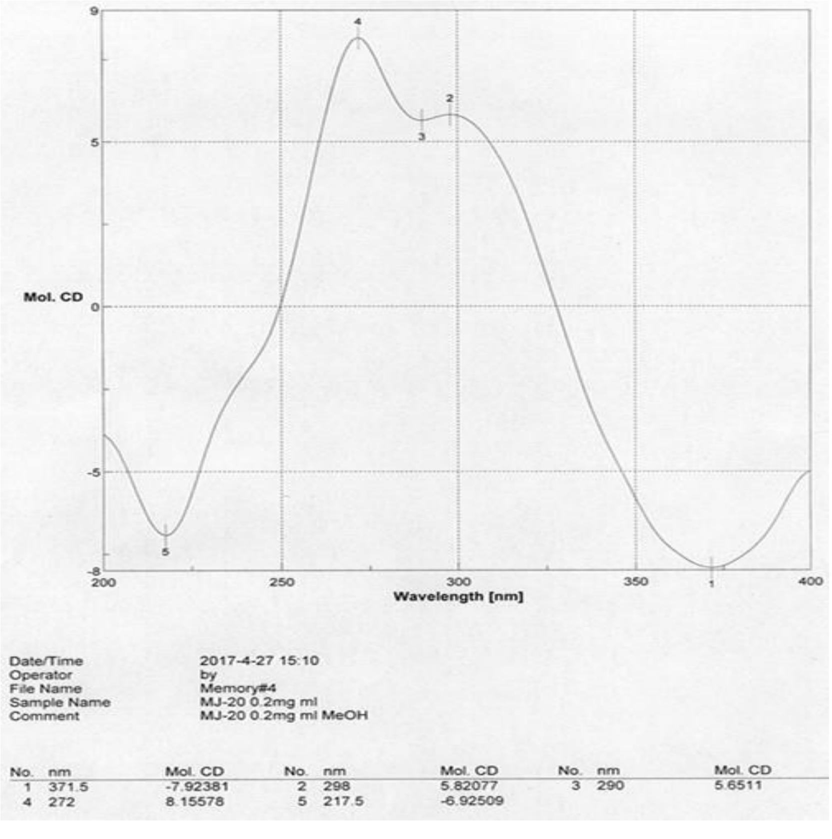

CD spectrum of **1**

NMR, HRESIMS, IR, and CD spectra of 2

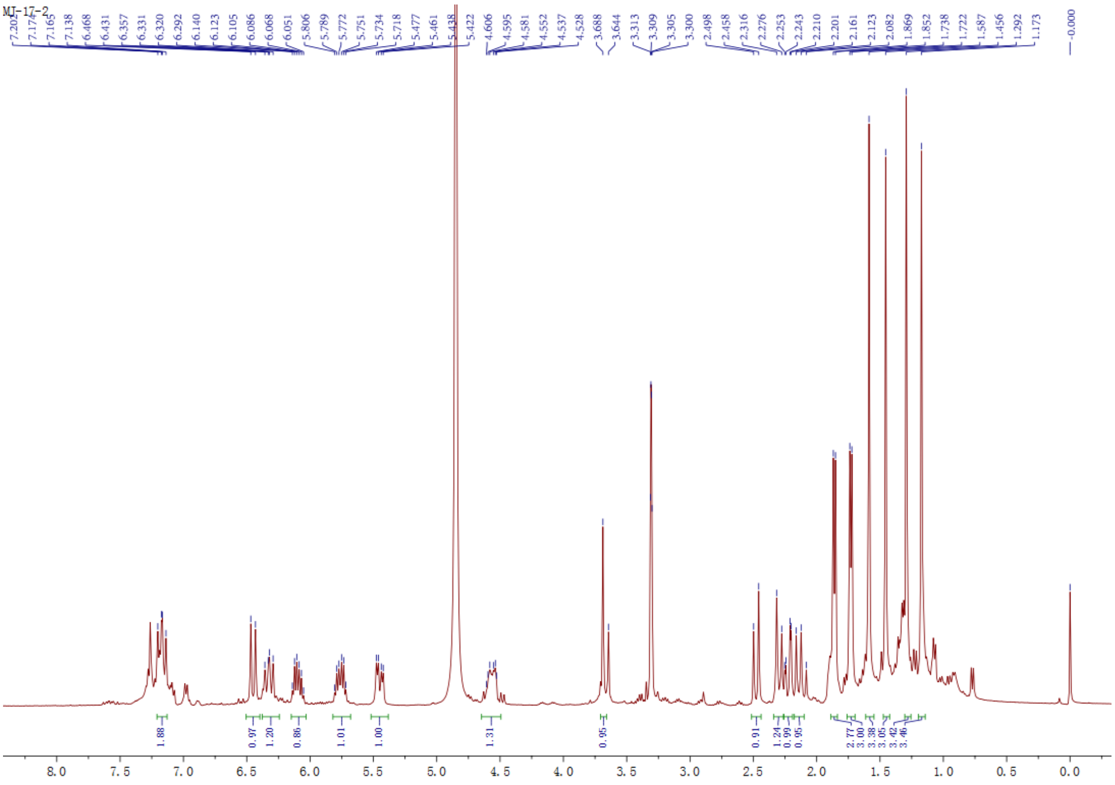

^{1}H NMR spectrum of **2** (CD_3OD, 400MHz)

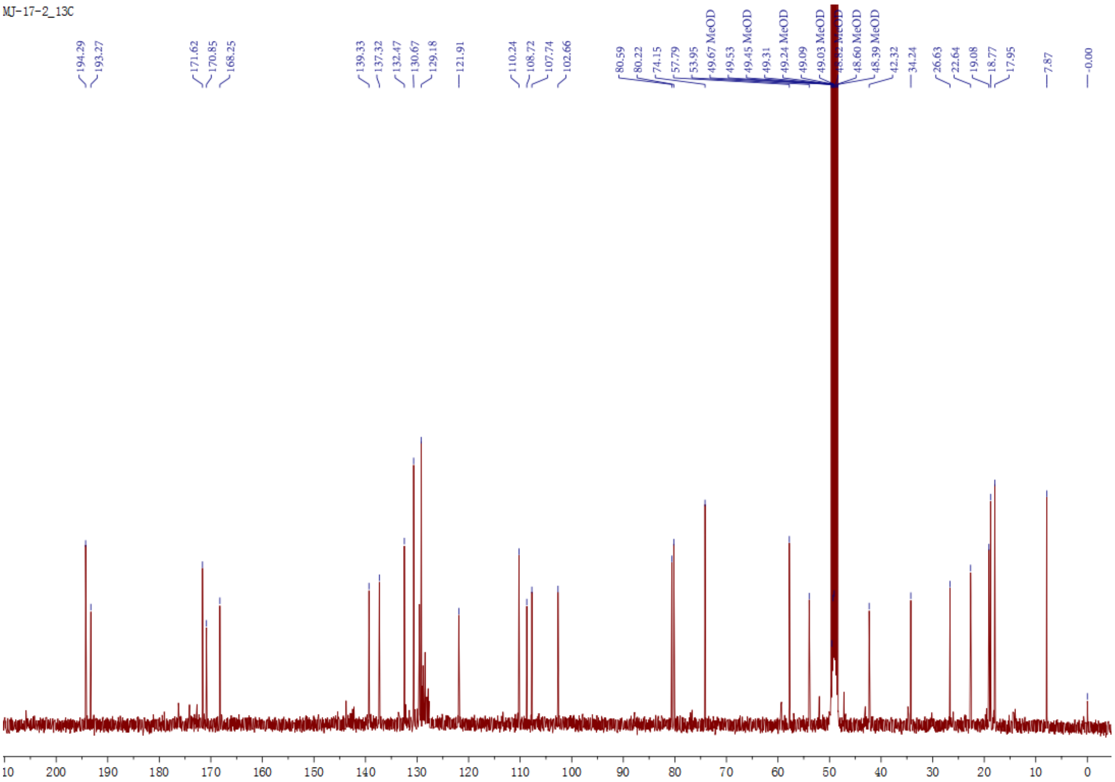

^{13}C NMR spectrum of **2** (CD_3OD, 100MHz)

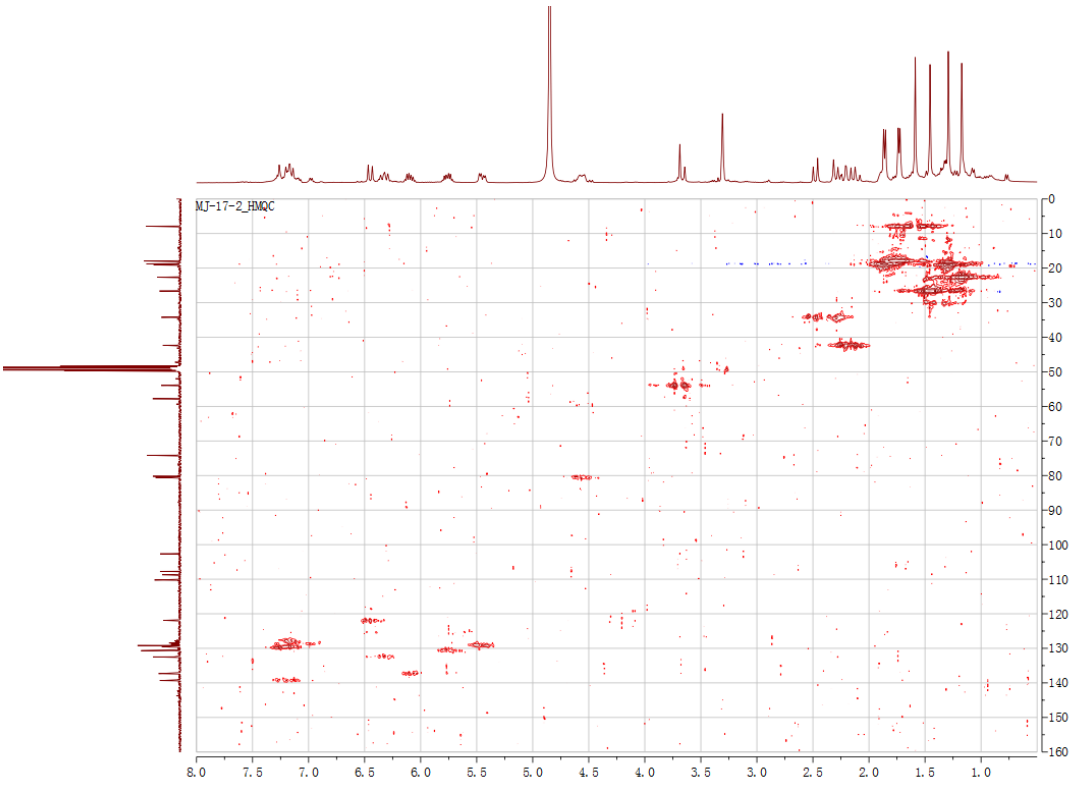

HMQC spectrum of **2**

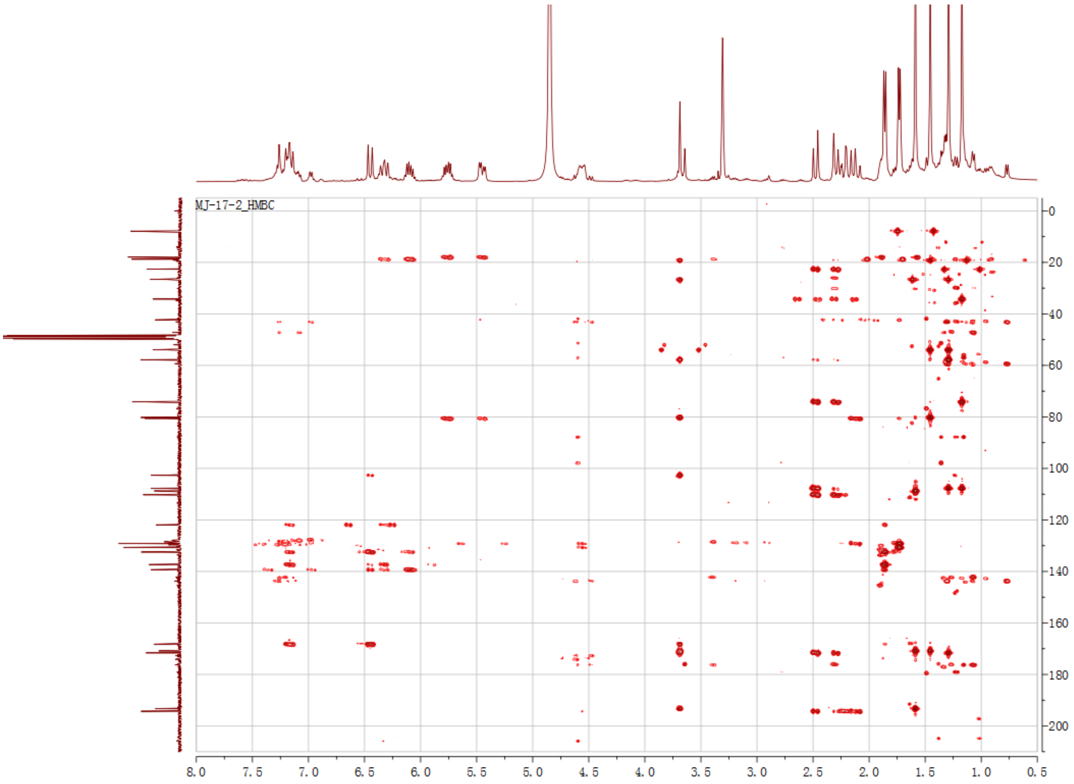

HMBC spectrum of **2**

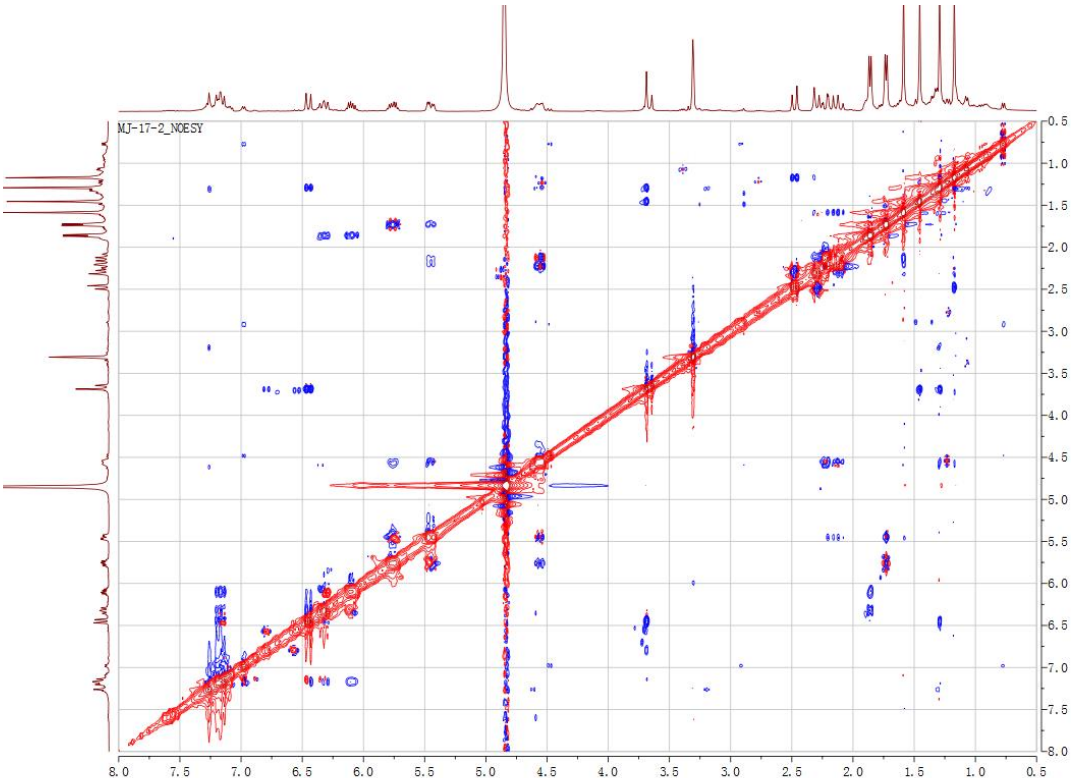

NOESY spectrum of **2**

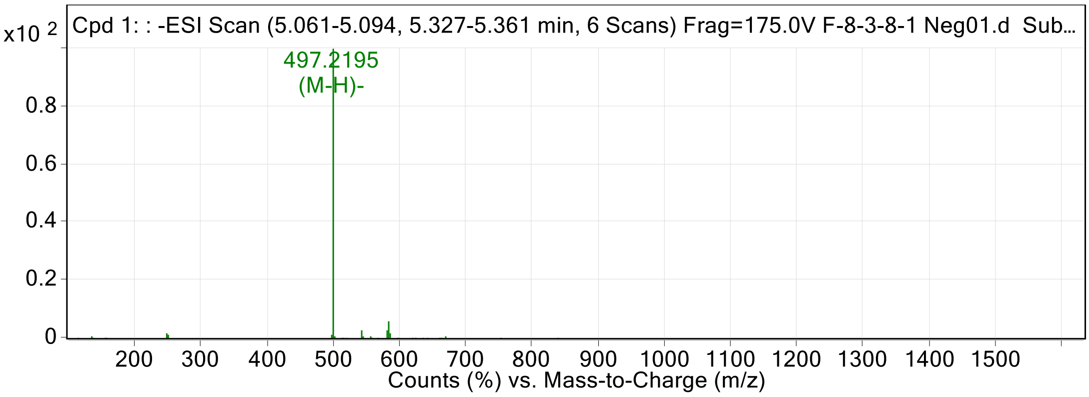

m/z	*Calc m/z*	Diff(ppm)	z	Abund	Formula	Ion
497.2195	497.2181	2.76	-1	226127.9	$C_{28}H_{33}O_8$	(M-H)-

HRESIMS spectrum of **2**

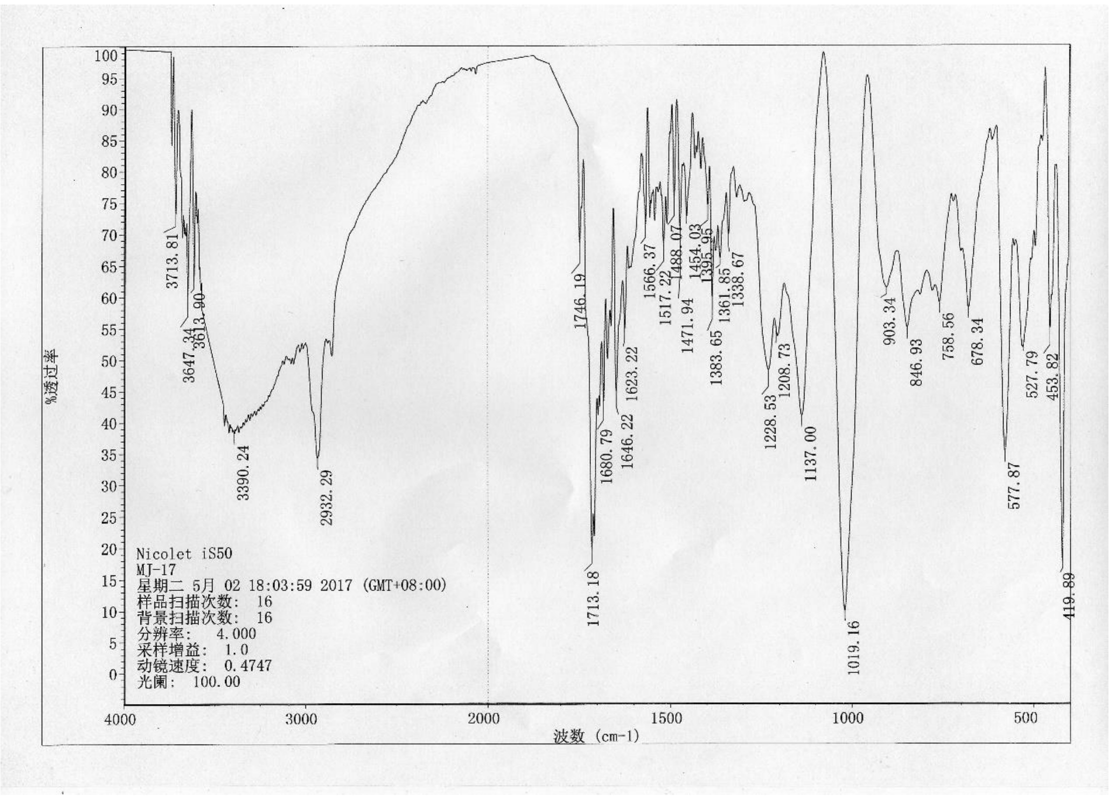

IR spectrum of **2**

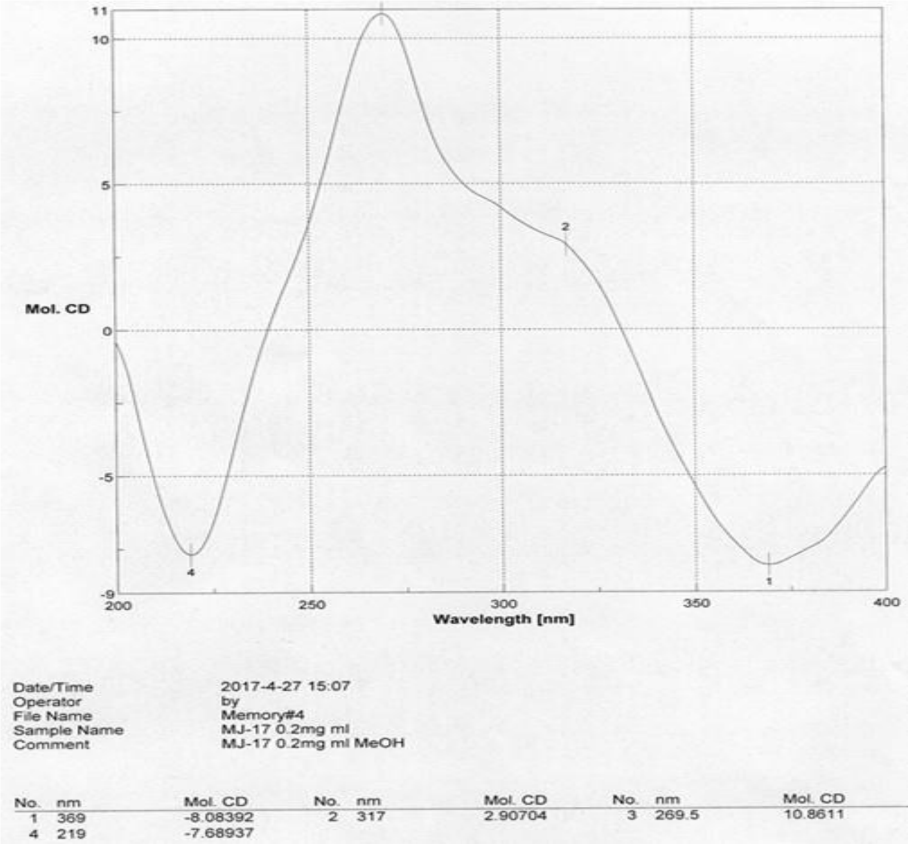

CD spectrum of **2**

NMR, HRESIMS, IR, and CD spectra of 3

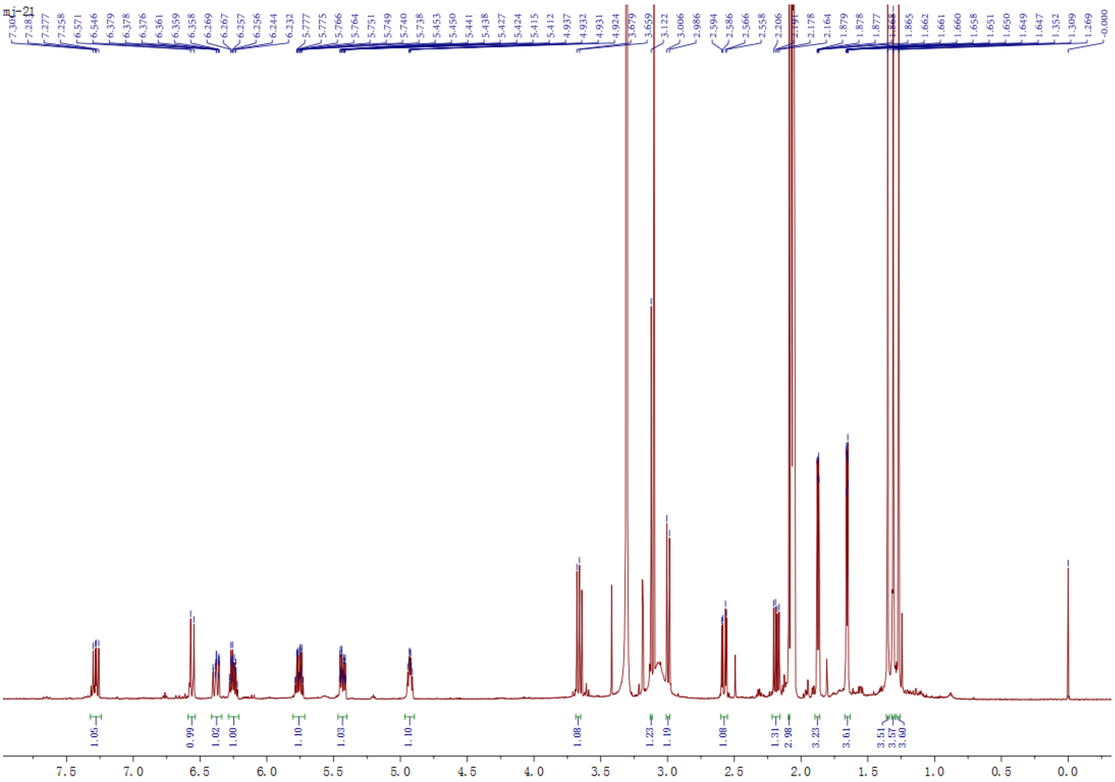

^{1}H NMR spectrum of **3** (CD_3COCD_3, 600MHz)

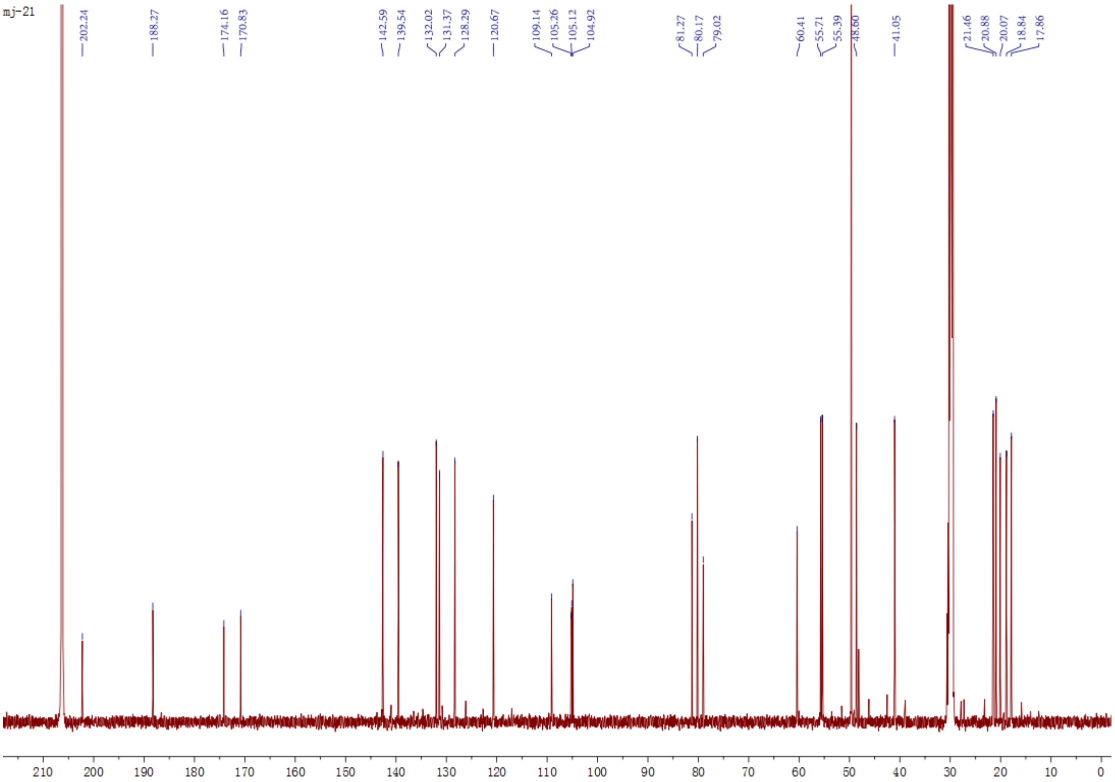

^{13}C NMR spectrum of **3** (CD_3COCD_3, 150MHz)

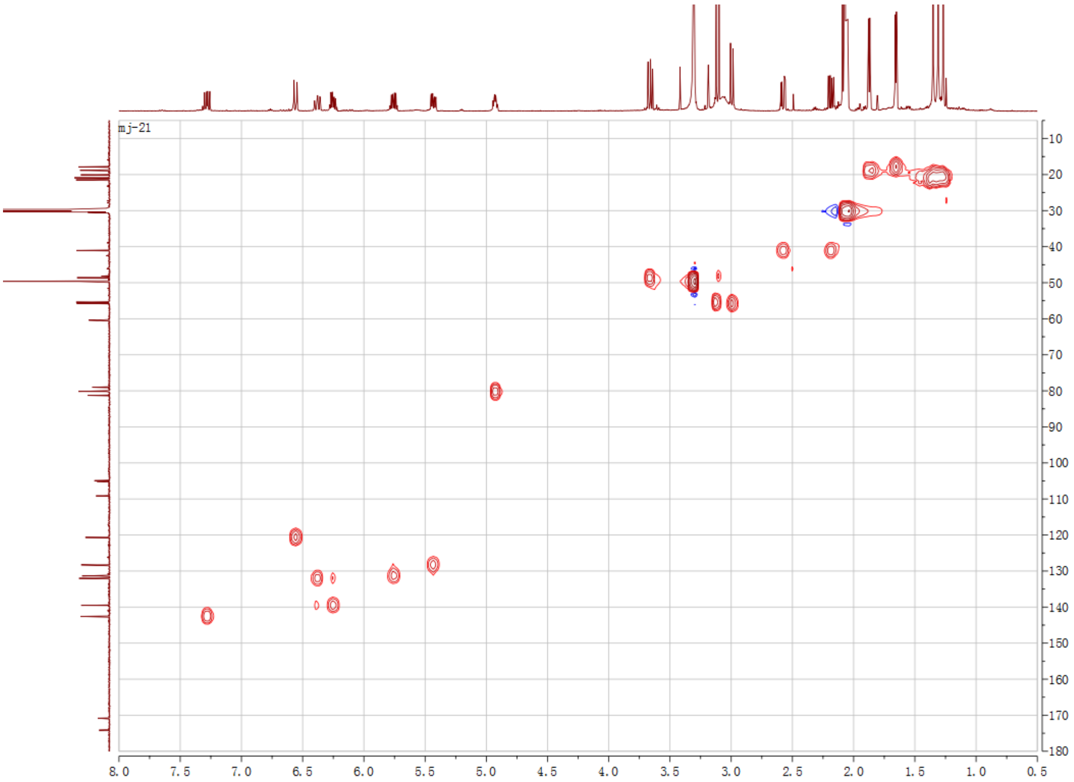

HSQC spectrum of **3**

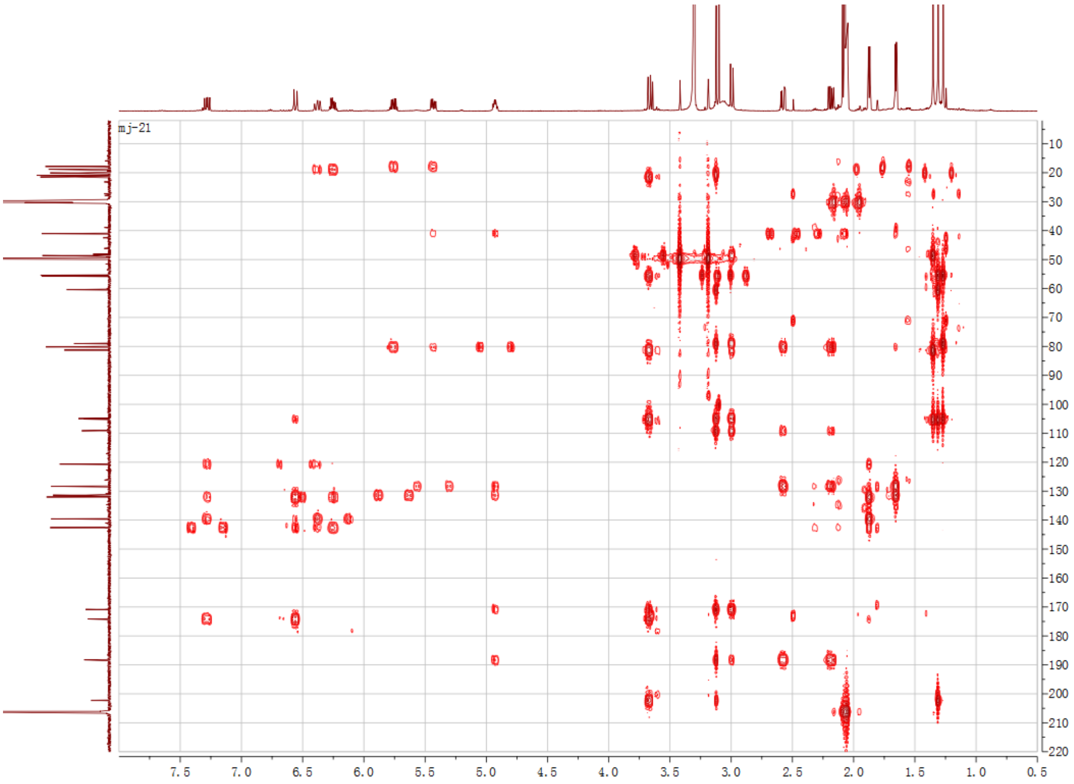

HMBC spectrum of **3**

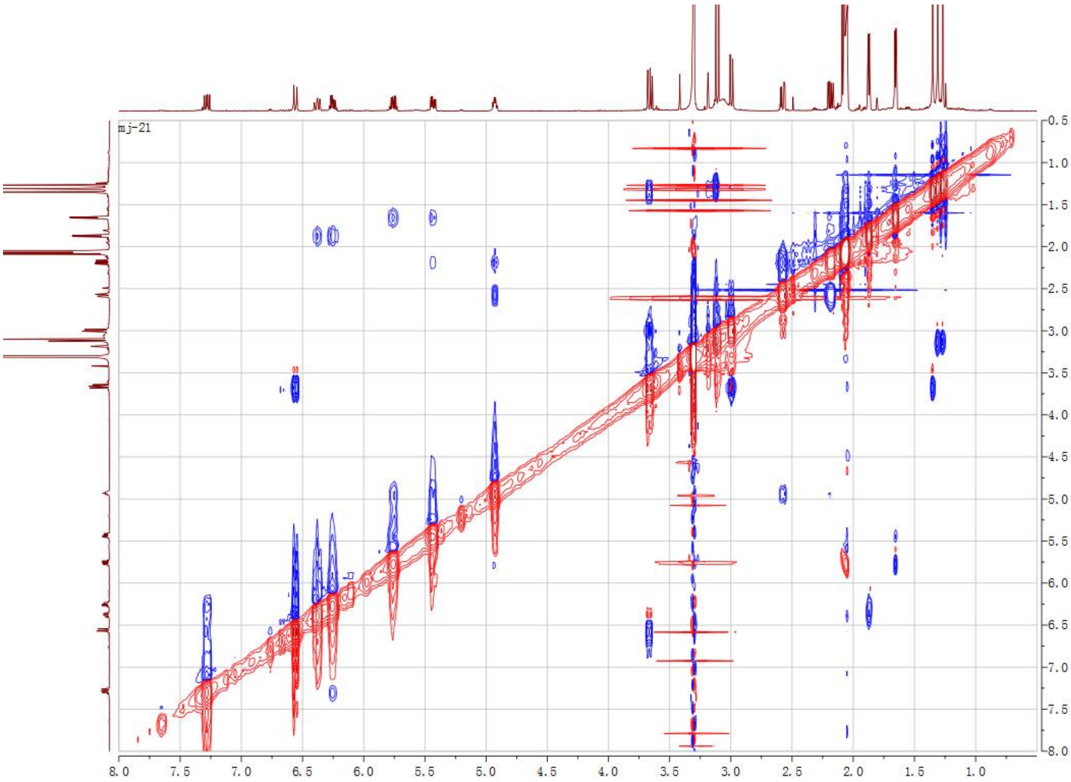

NOESY spectrum of **3**

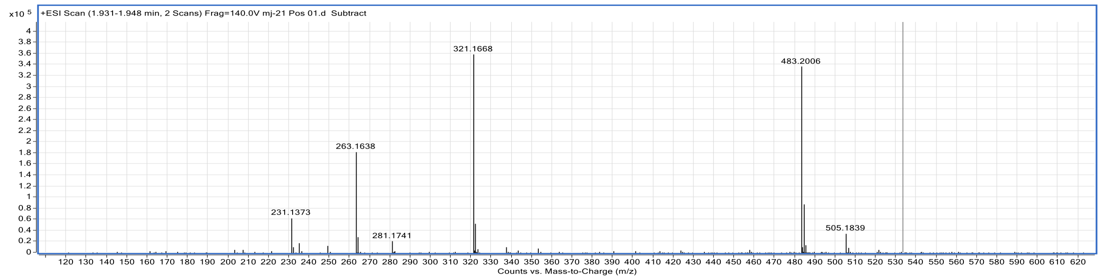

m/z	*Calc m/z*	Diff(ppm)	z	Abund	Formula	Ion
483.2006	483.2013	1.5	1	336553.2	$C_{27}H_{31}O_8$	(M+H)+

HRESIMS spectrum of **3**

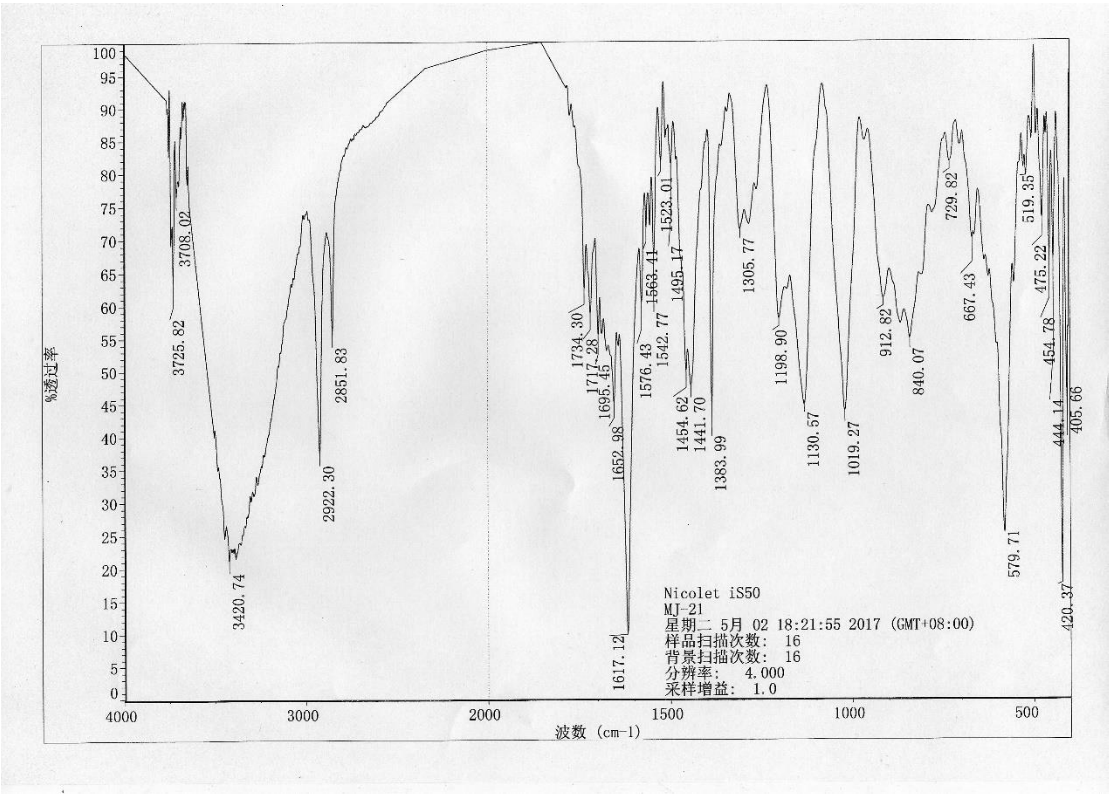

IR spectrum of **3**

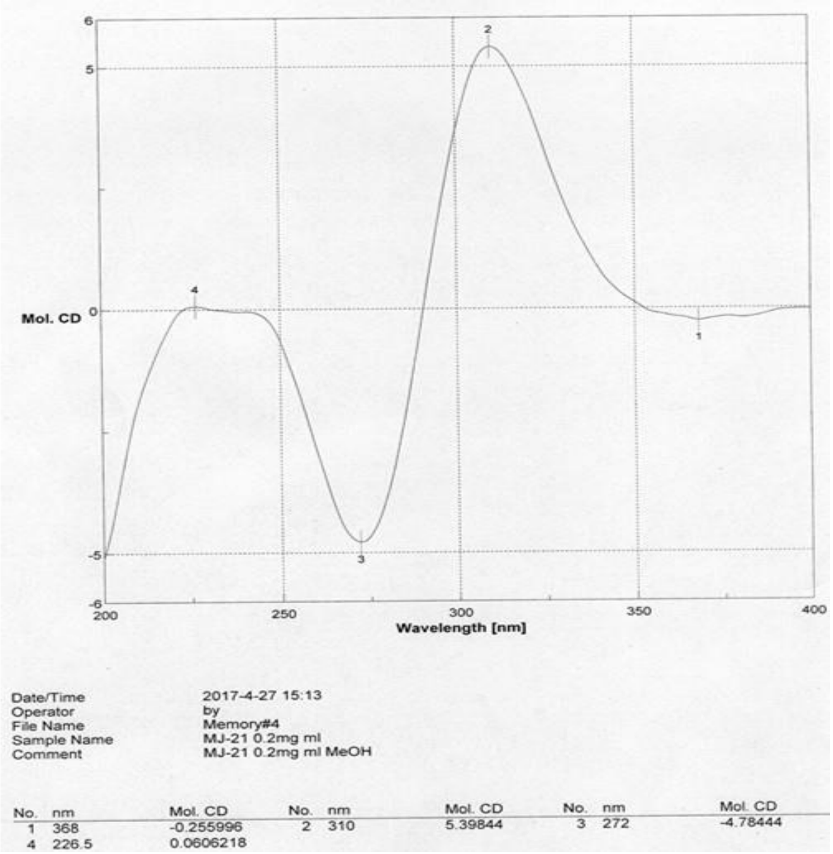

CD spectrum of **3**

NMR, HRESIMS, IR, and CD spectra of 4

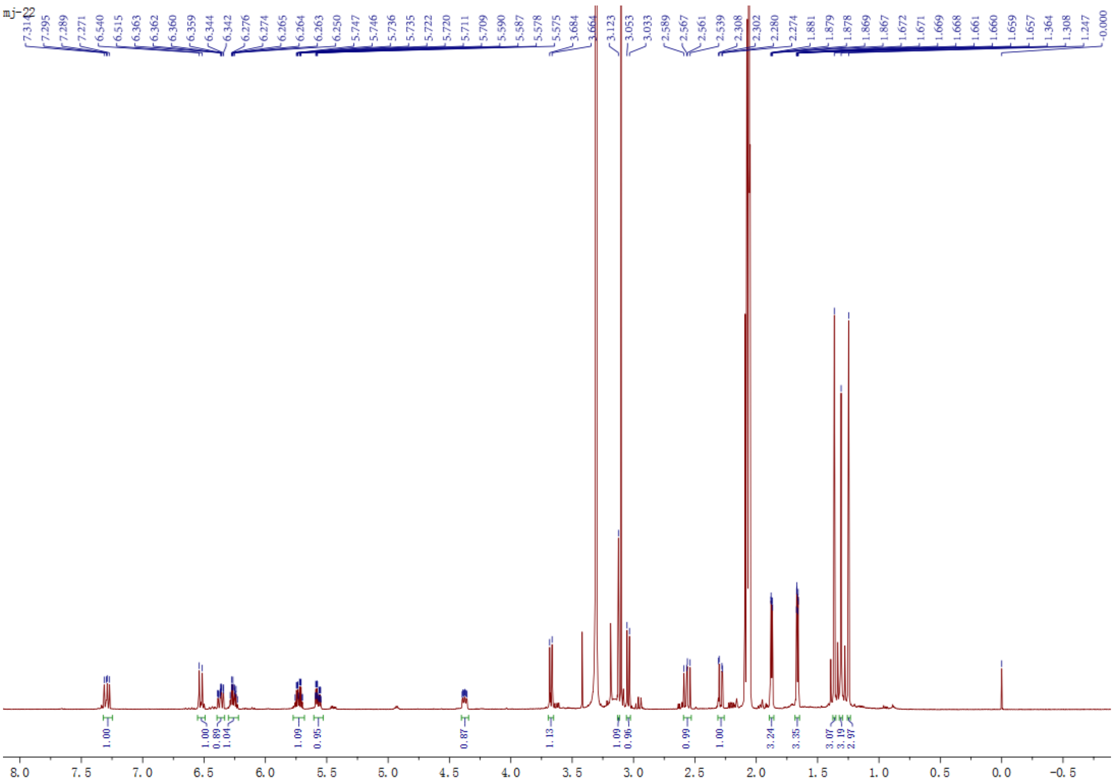

^{1}H NMR spectrum of **4** (CD_3COCD_3, 600MHz)

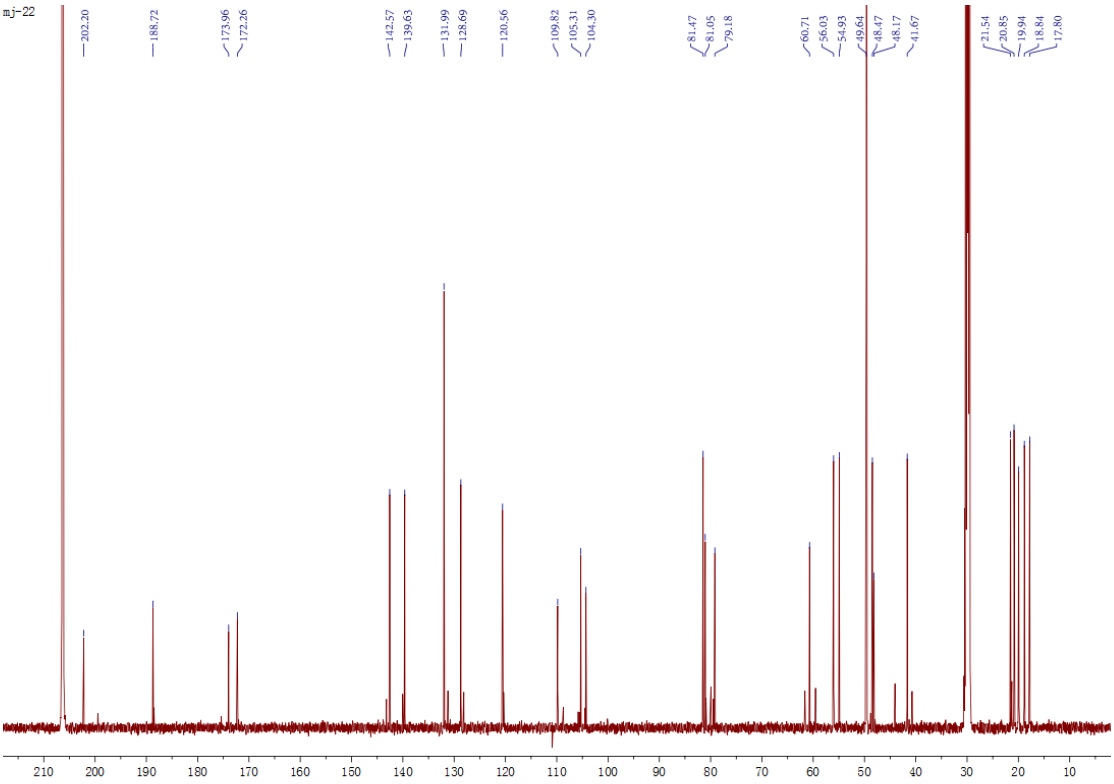

^{13}C NMR spectrum of **4** (CD_3COCD_3, 150MHz)

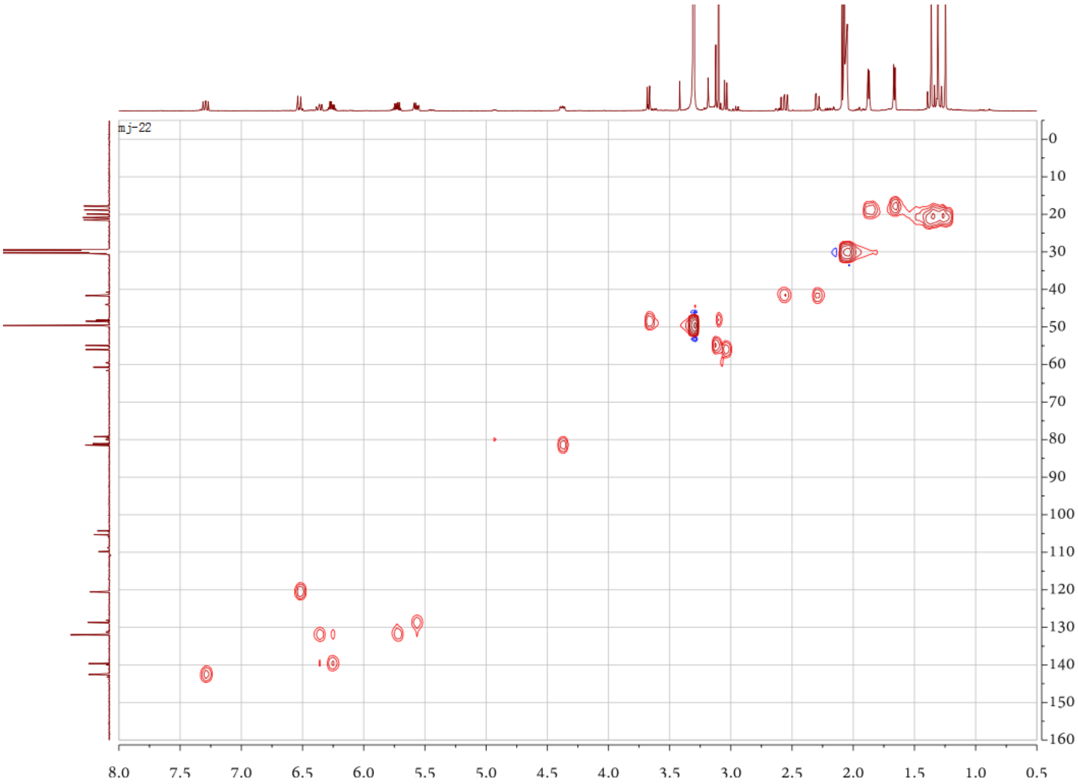

HSQC spectrum of **4**

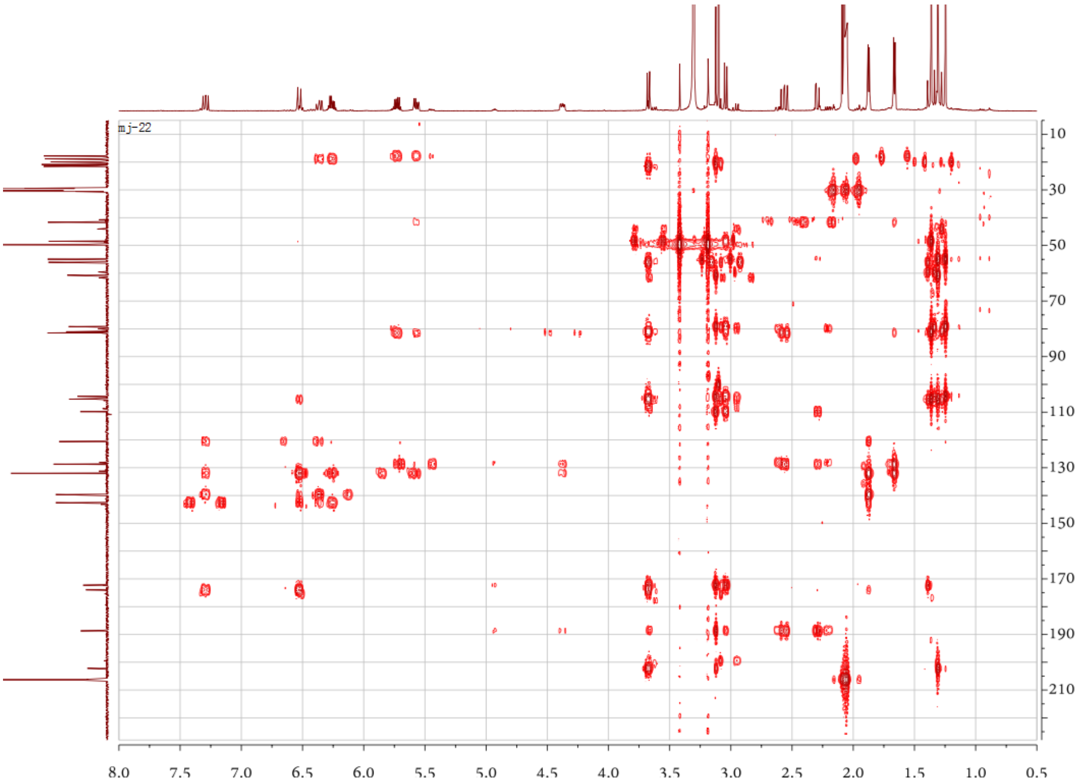

HMBC spectrum of **4**

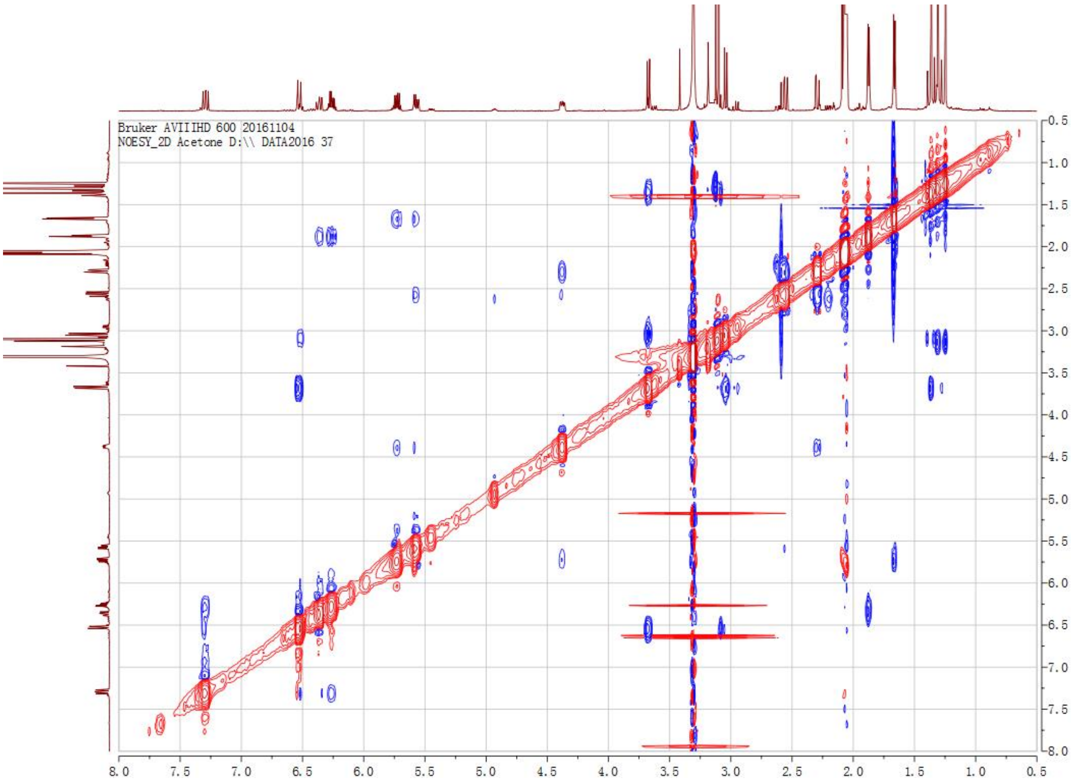

NOESY spectrum of **4**

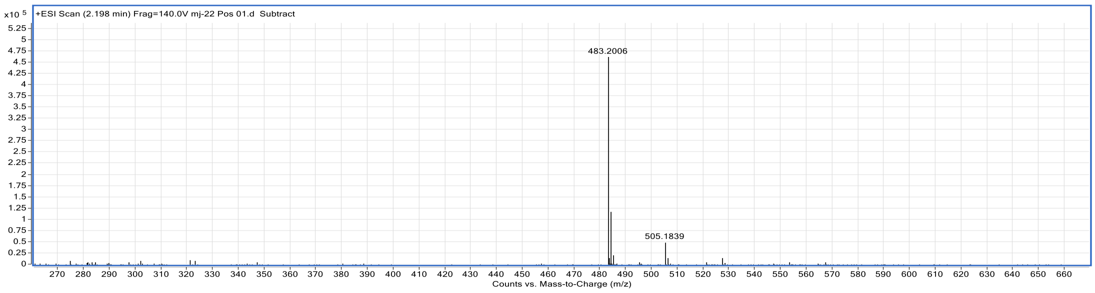

m/z	***Calc m/z***	**Diff(ppm)**	**z**	**Abund**	**Formula**	**Ion**
483.2006	483.2013	1.45	1	462870.8	$C_{27}H_{31}O_8$	(M+H)+

HRESIMS spectrum of **4**

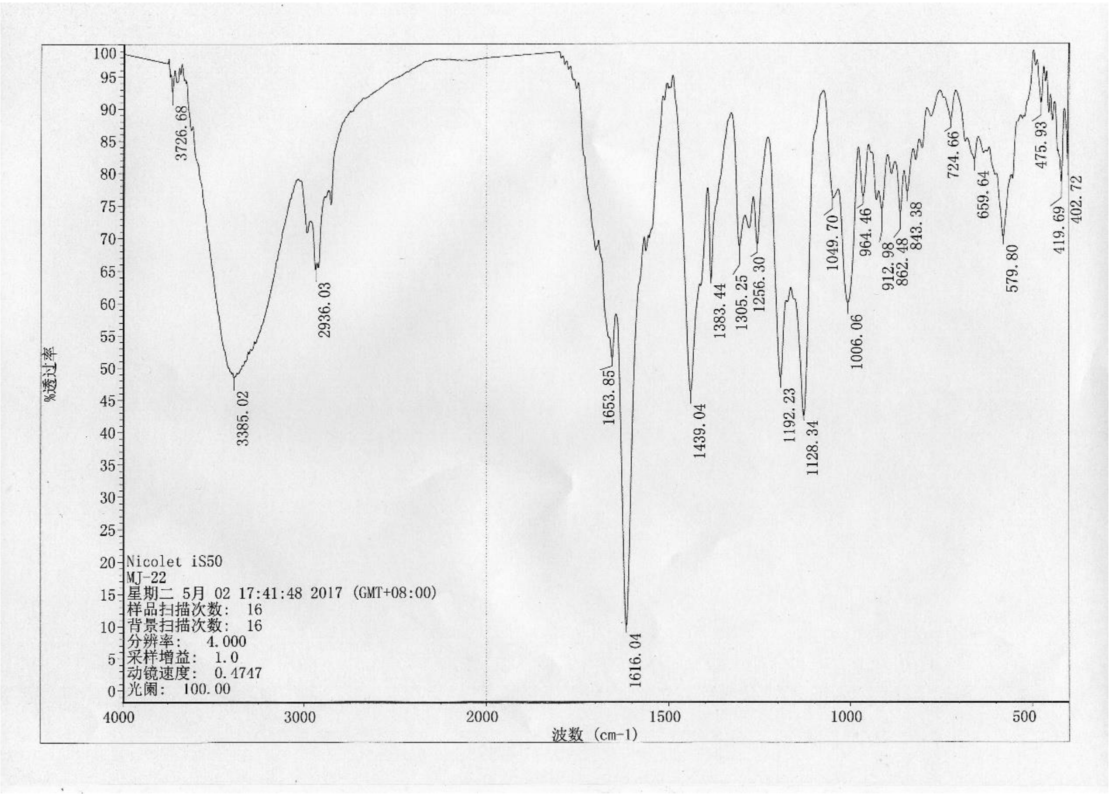

IR spectrum of **4**

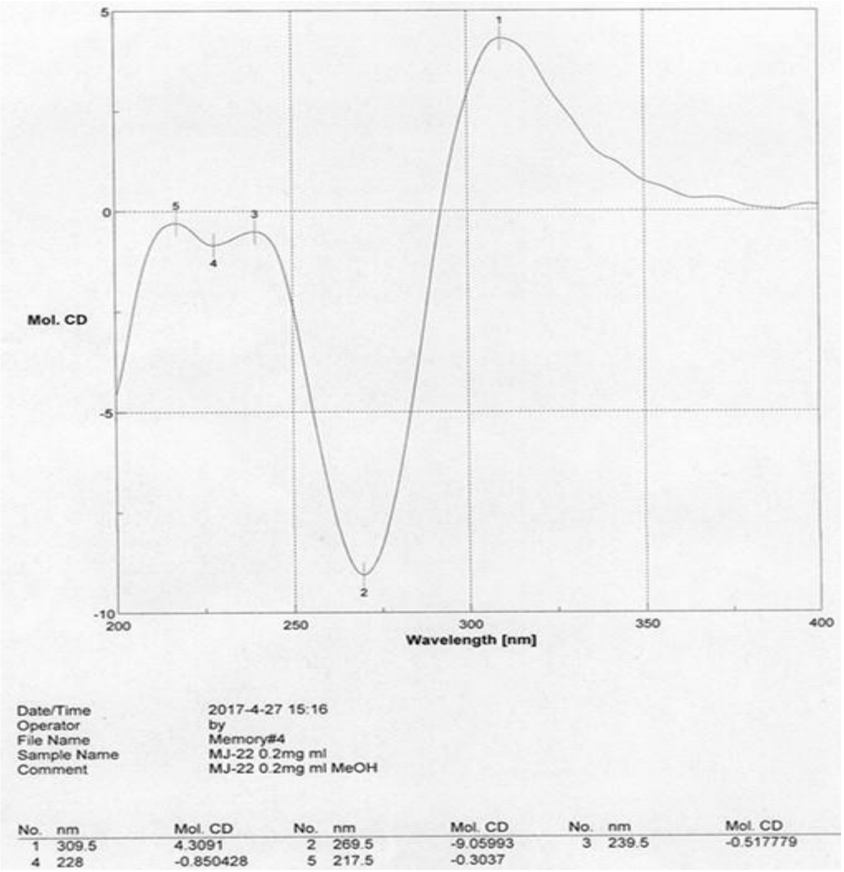

CD spectrum of **4**

NMR, HRESIMS, IR, and CD spectra of 5

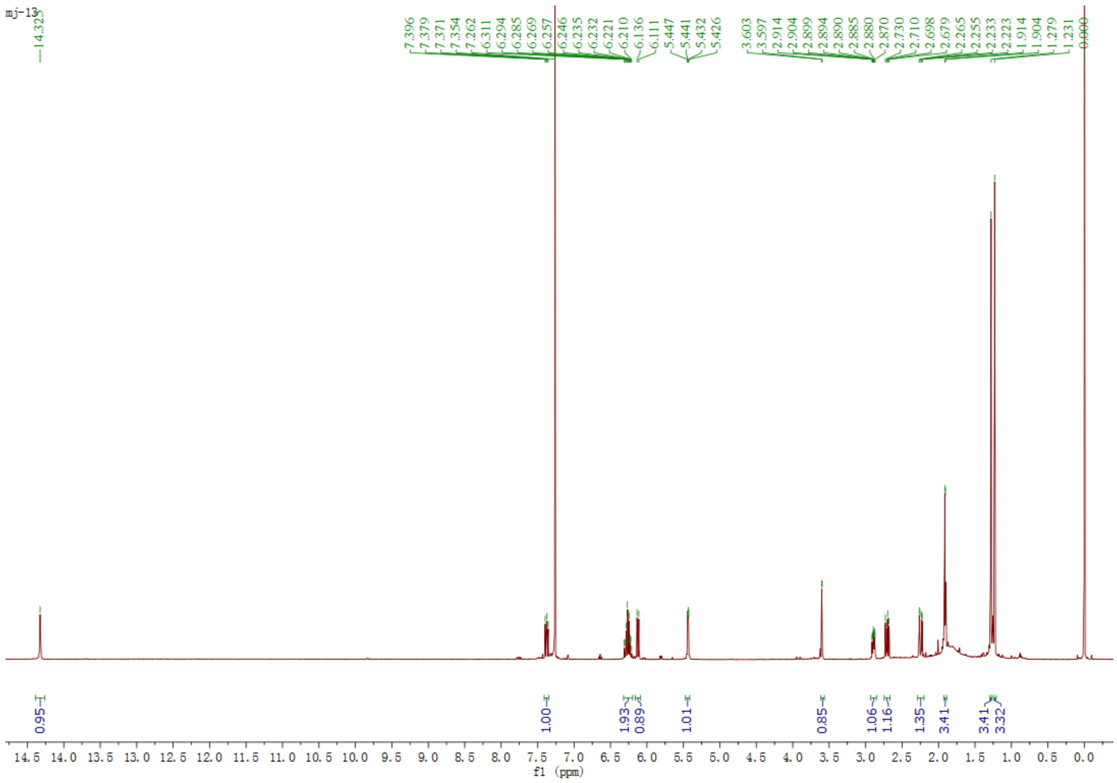

^{1}H NMR spectrum of **5** ($CDCl_3$, 600MHz)

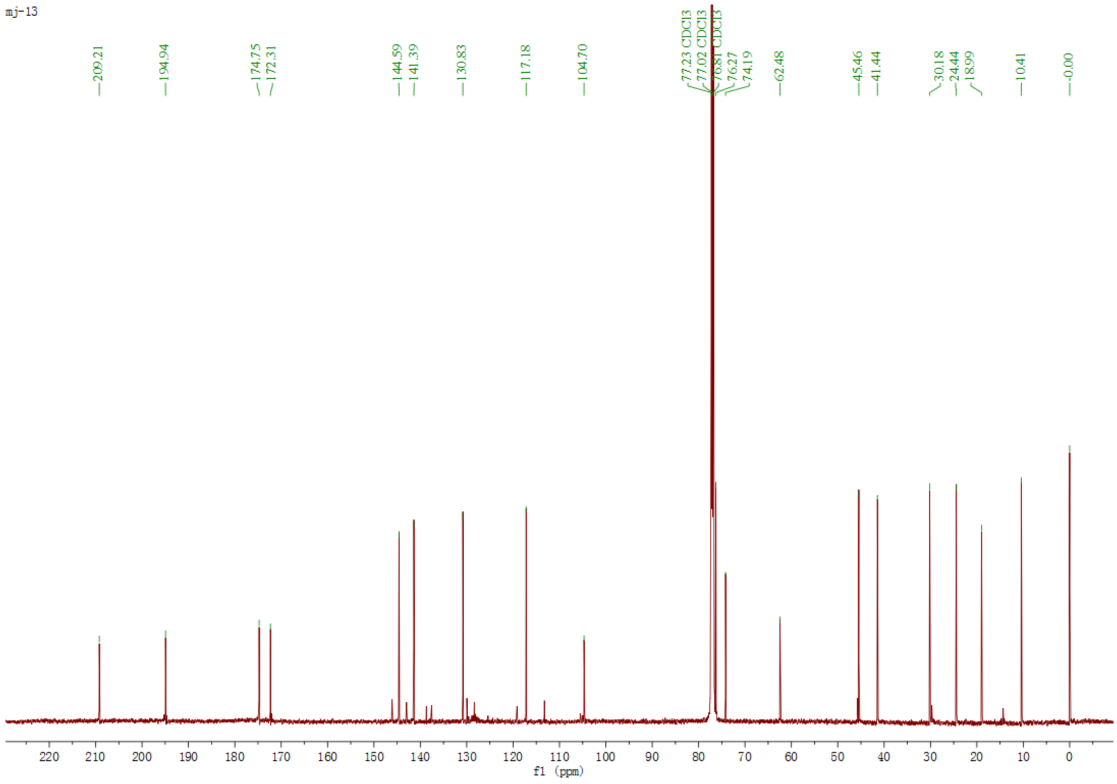

^{13}C NMR spectrum of **5** ($CDCl_3$, 150MHz)

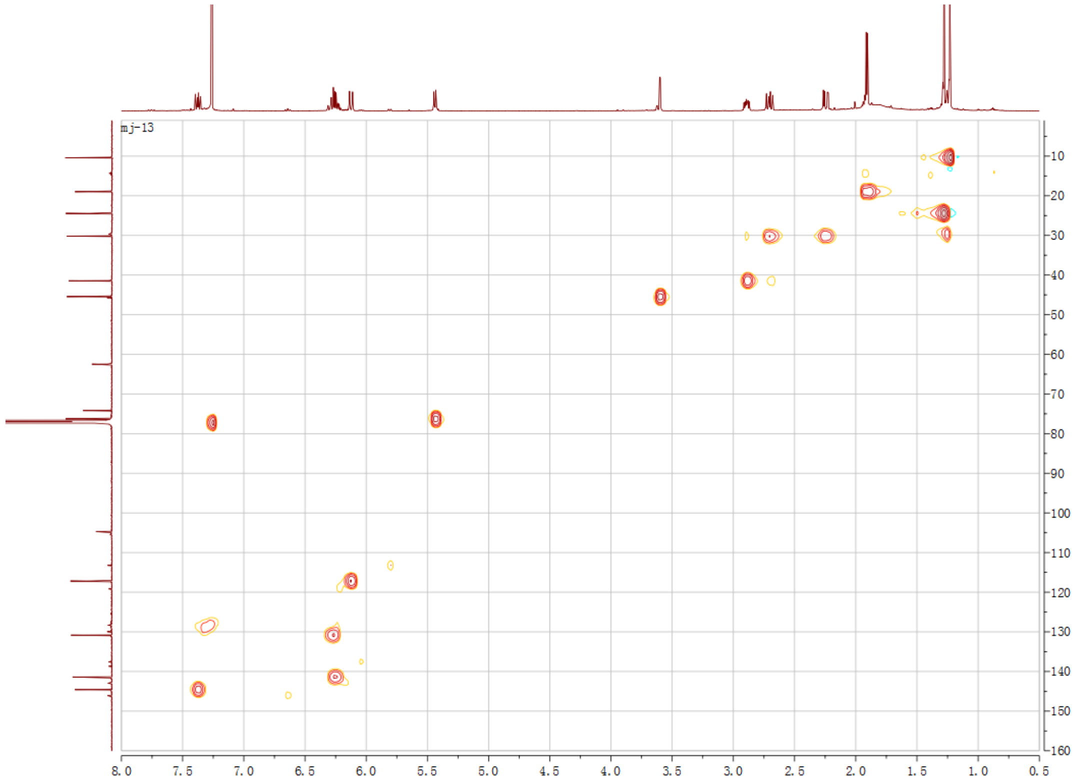

HSQC spectrum of **5**

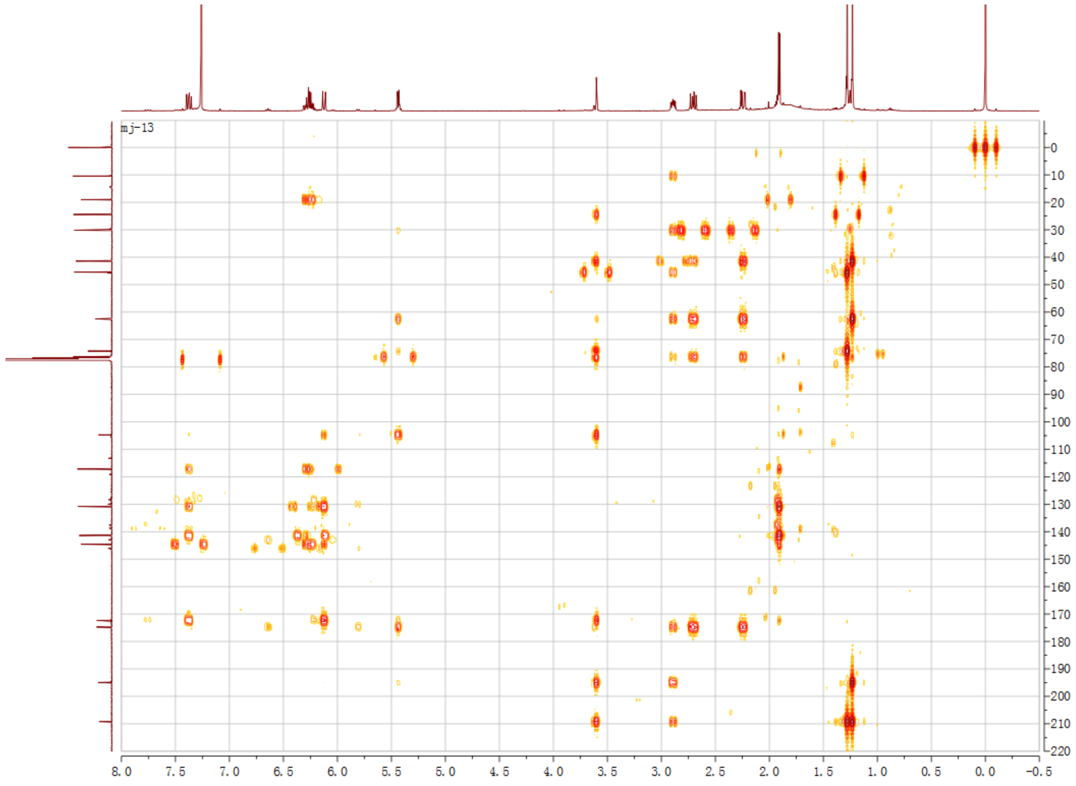

HMBC spectrum of **5**

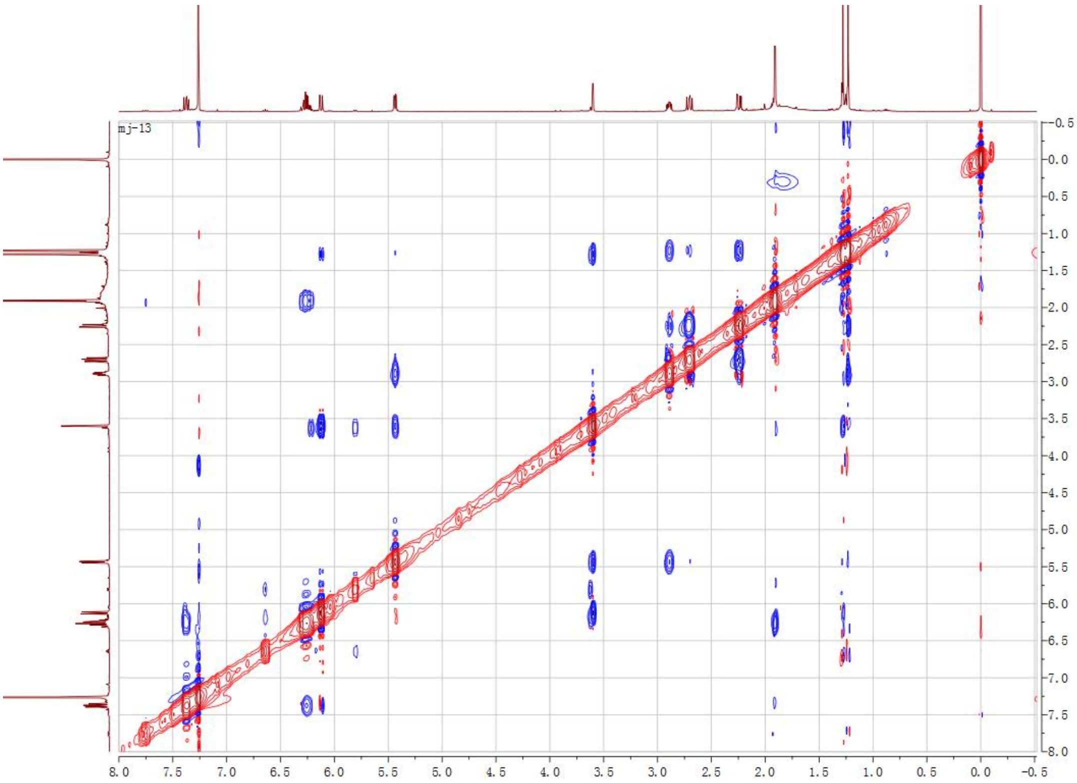

NOESY spectrum of **5**

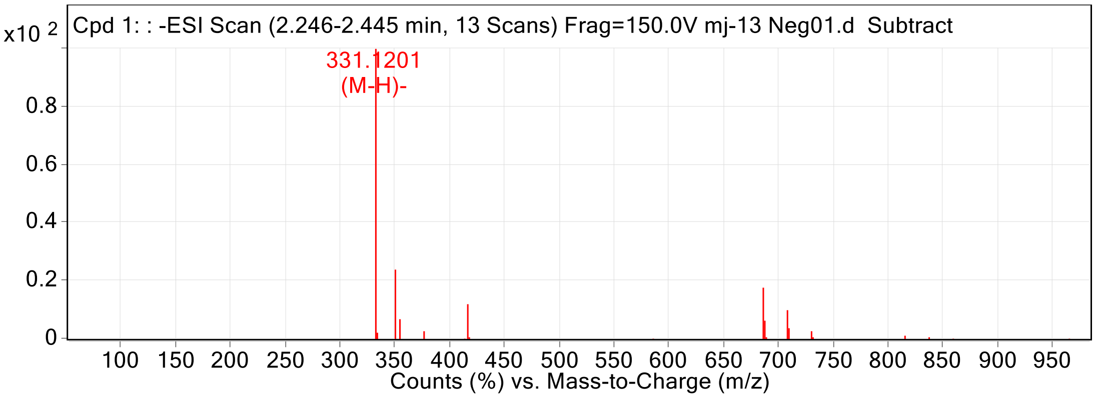

m/z	*Calc m/z*	Diff(ppm)	z	Abund	Formula	Ion
331.1201	331.1187	4.17	-1	9341.4	C18H19O6	(M-H)-

HRESIMS spectrum of **5**

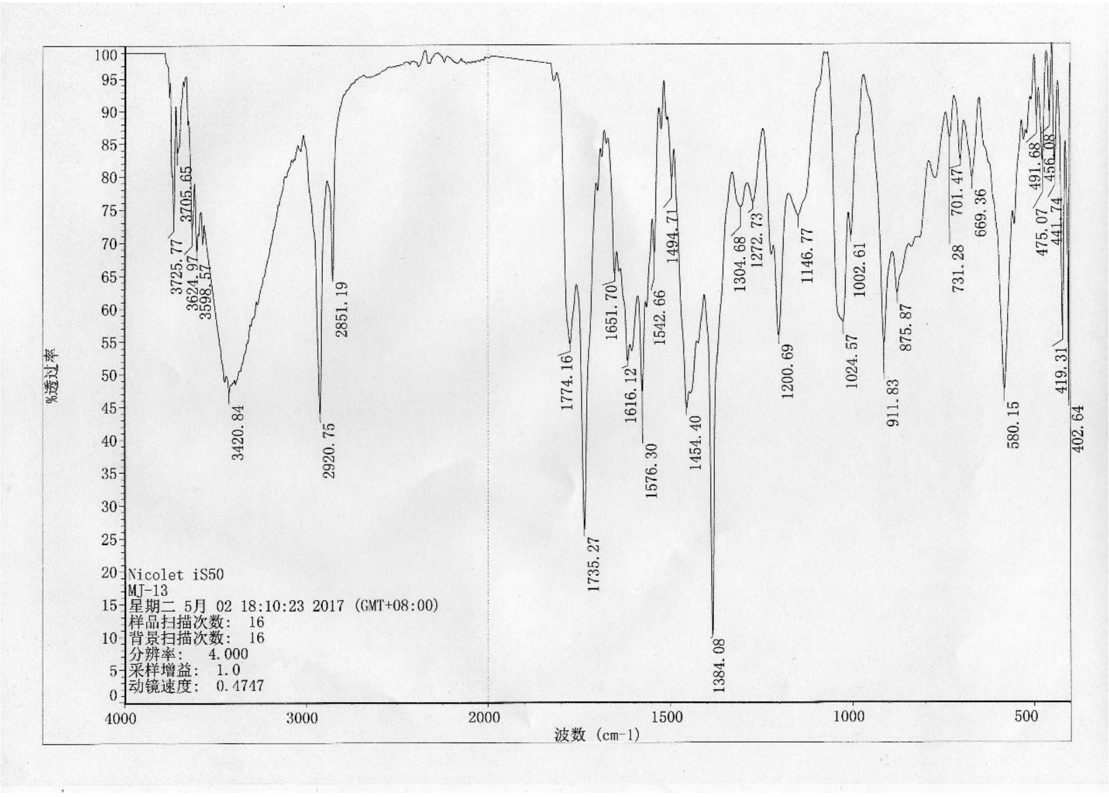

IR spectrum of **5**

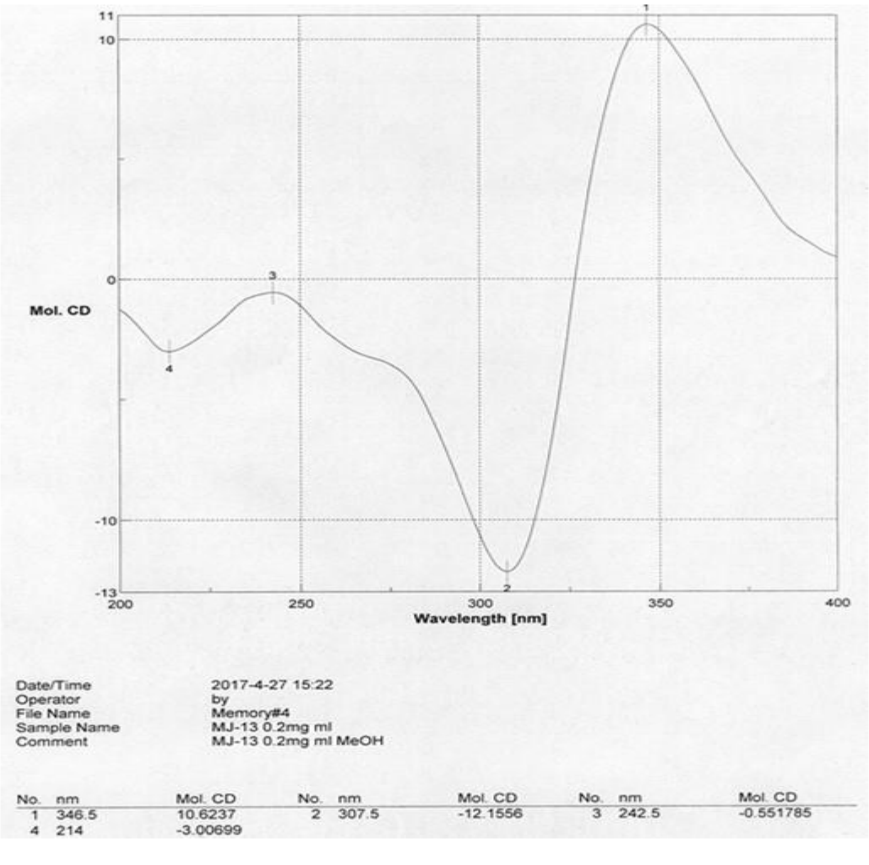

CD spectrum of **5**

NMR, HRESIMS, and IR spectra of 6

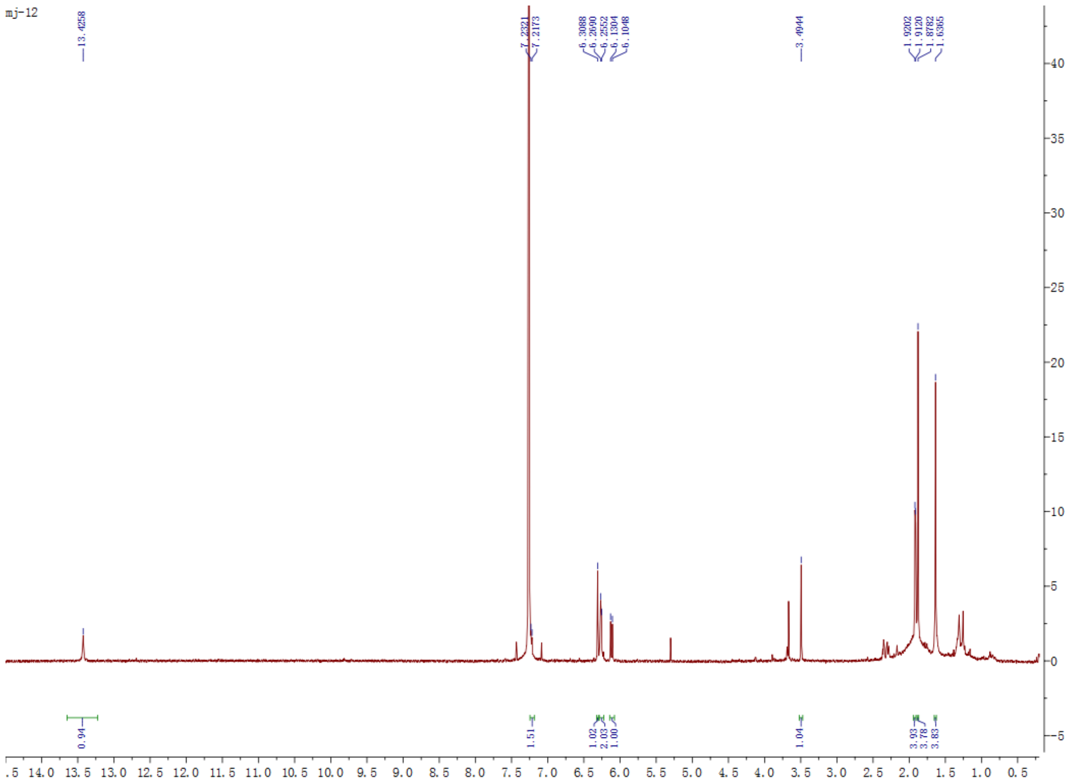

^{1}H NMR spectrum of **6** ($CDCl_3$, 600MHz)

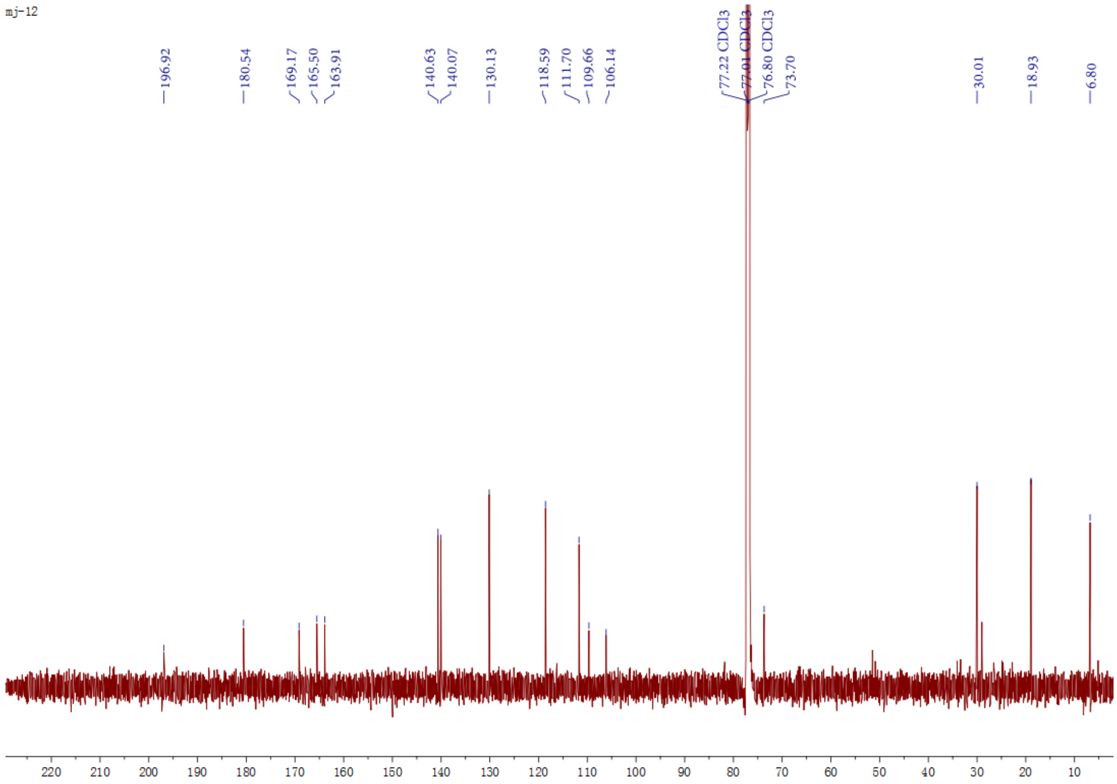

^{13}C NMR spectrum of **6** ($CDCl_3$, 150MHz)

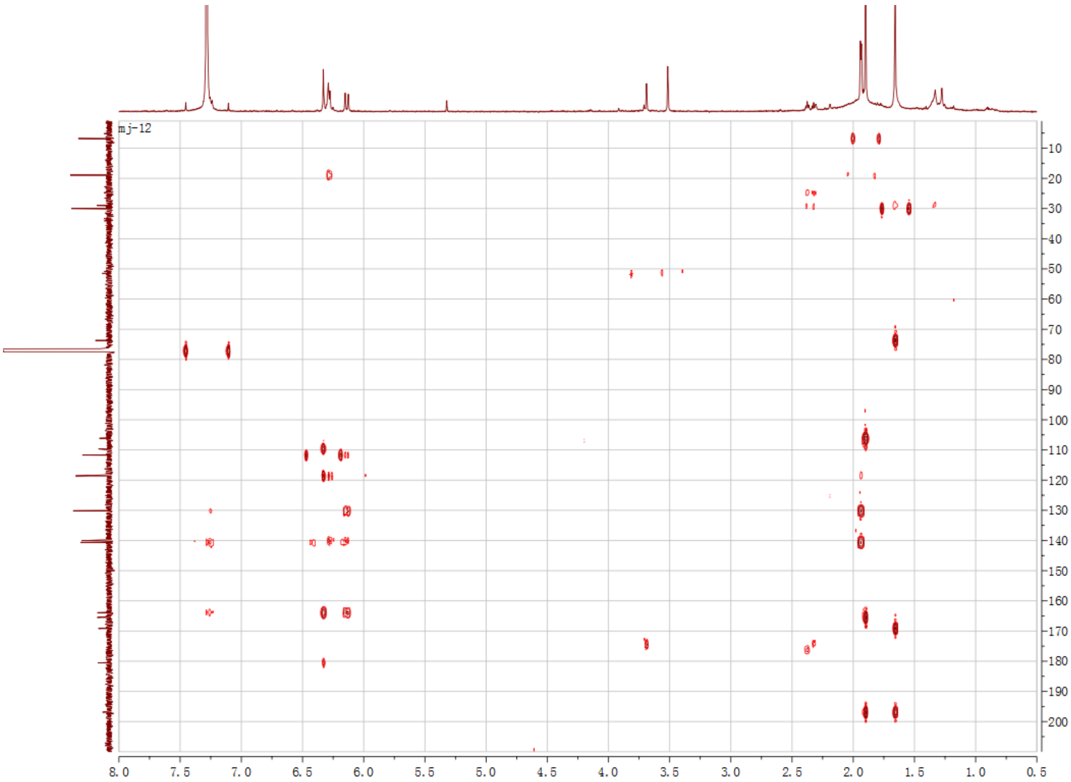

HMBC spectrum of **6**

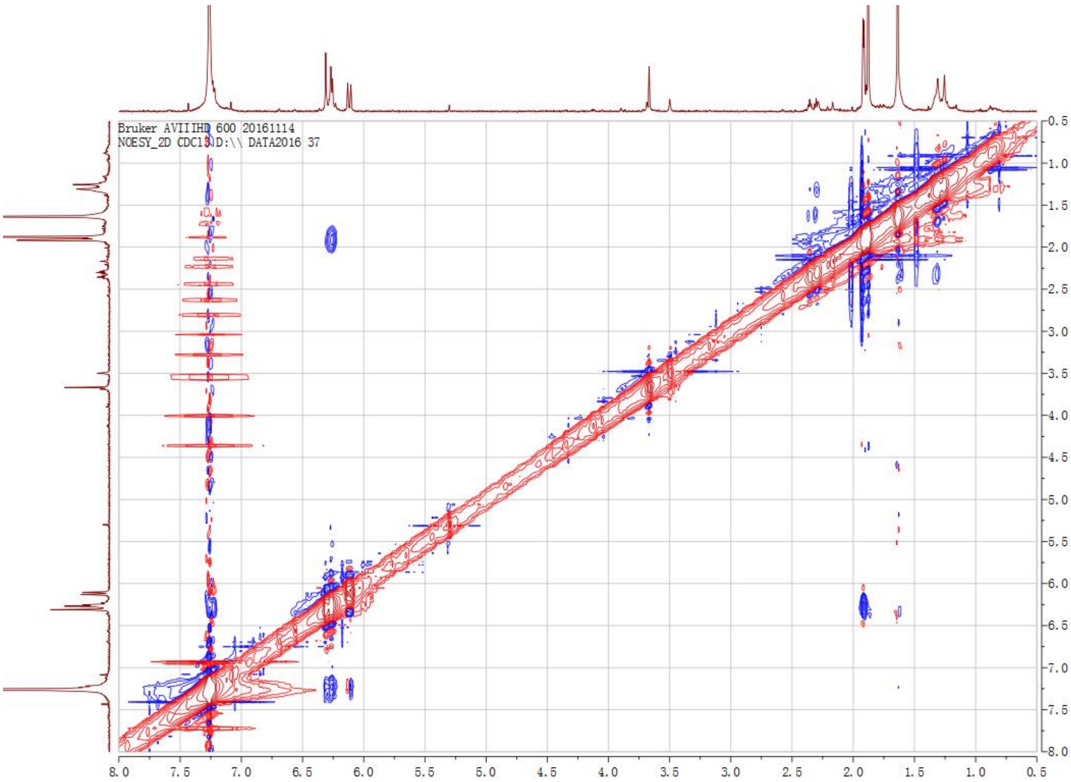

NOESY spectrum of **6**

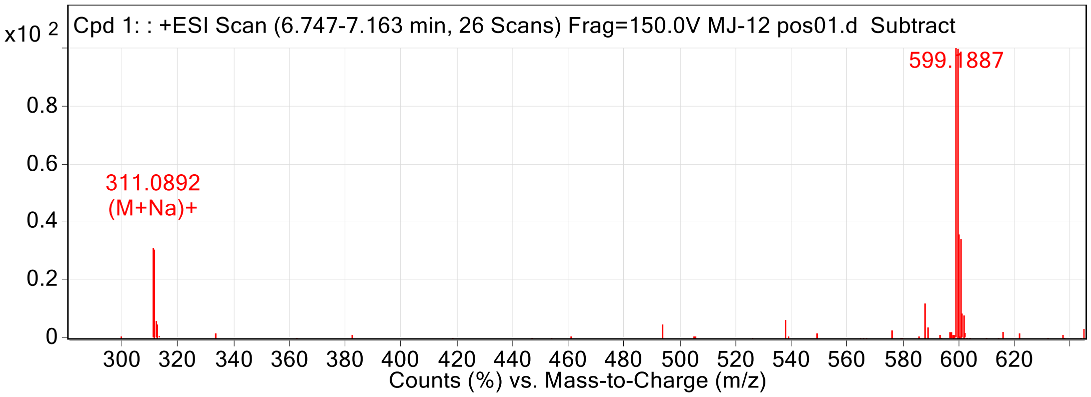

m/z	*Calc m/z*	Diff(ppm)	z	Abund	Formula	Ion
311.0892	311.0890	0.71	1	2249.3	C16H16NaO5	(M+Na)+
599.1887	599.1888	-0.15		7273.7	C32H32NaO10	(2M+Na)+

HRESIMS spectrum of **6**

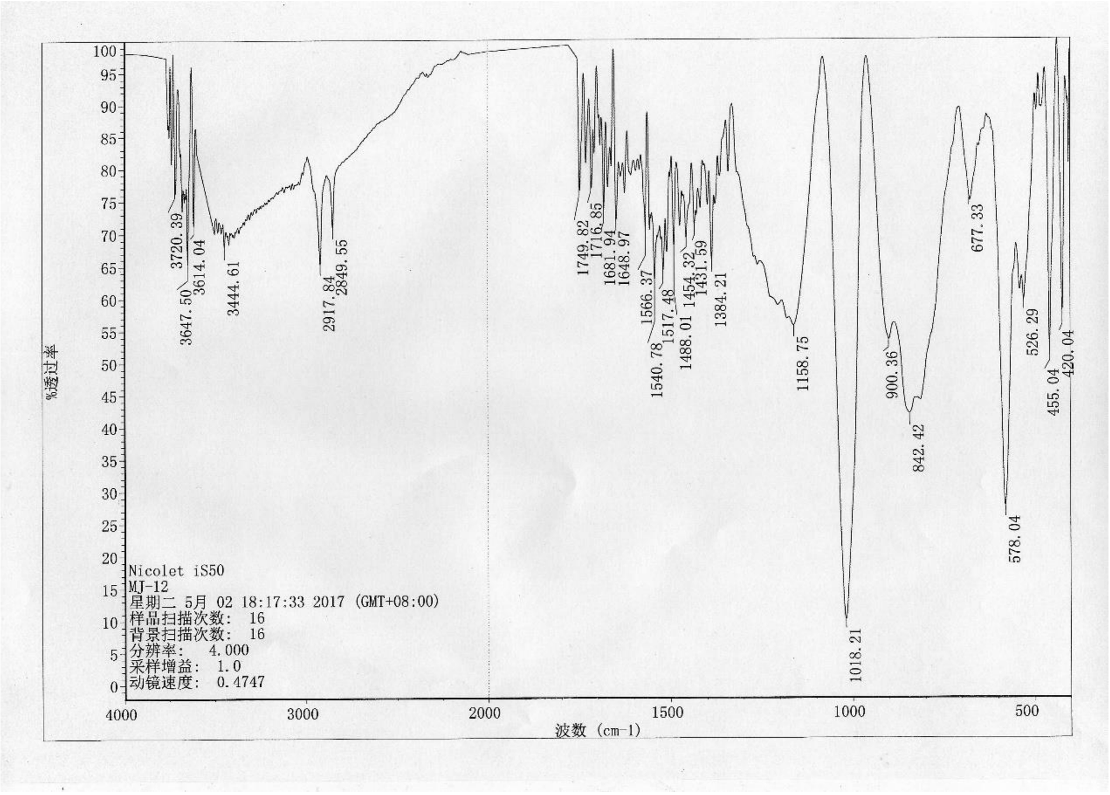

IR spectrum of **6**

NMR, HRESIMS, and IR spectra of 7

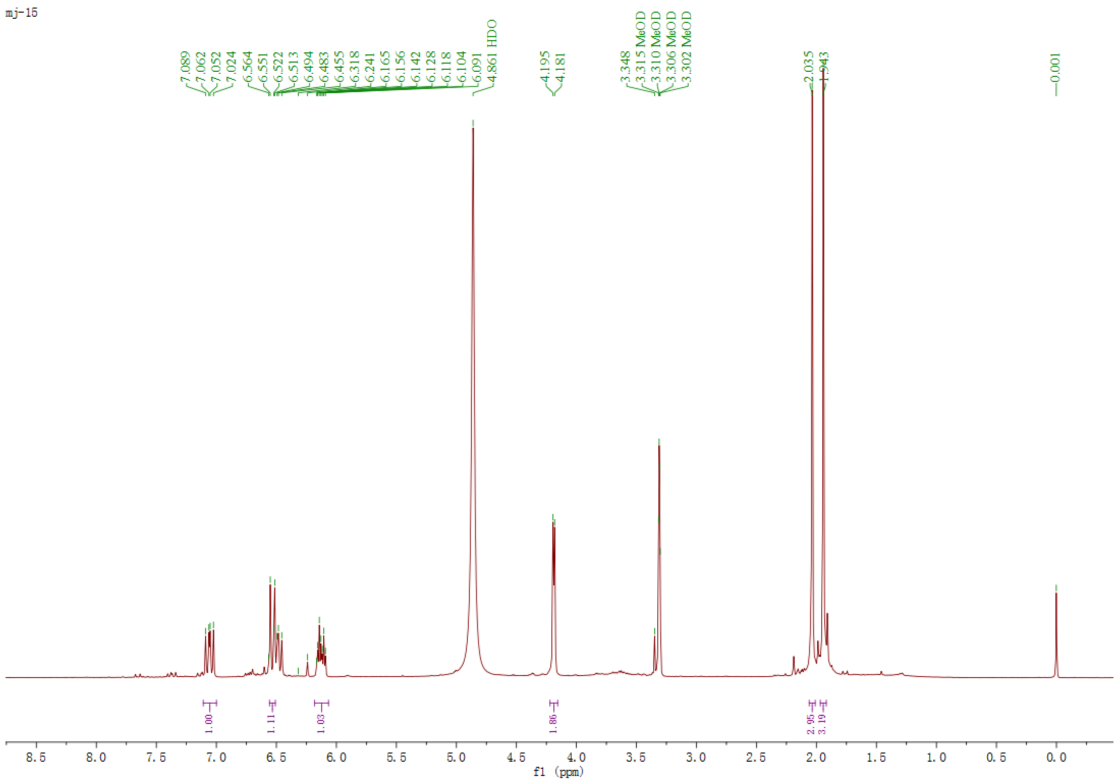

^{1}H NMR spectrum of **7** (CD_3OD, 400MHz)

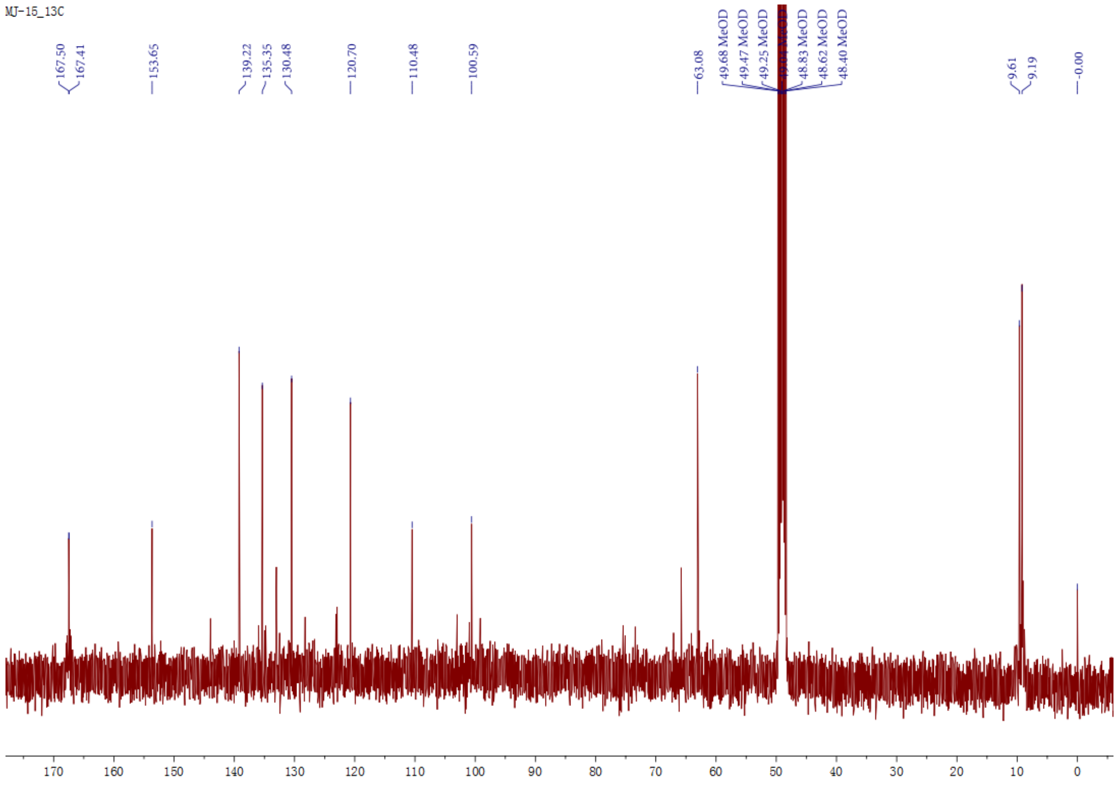

^{13}C NMR spectrum of **7** (CD_3OD, 100MHz)

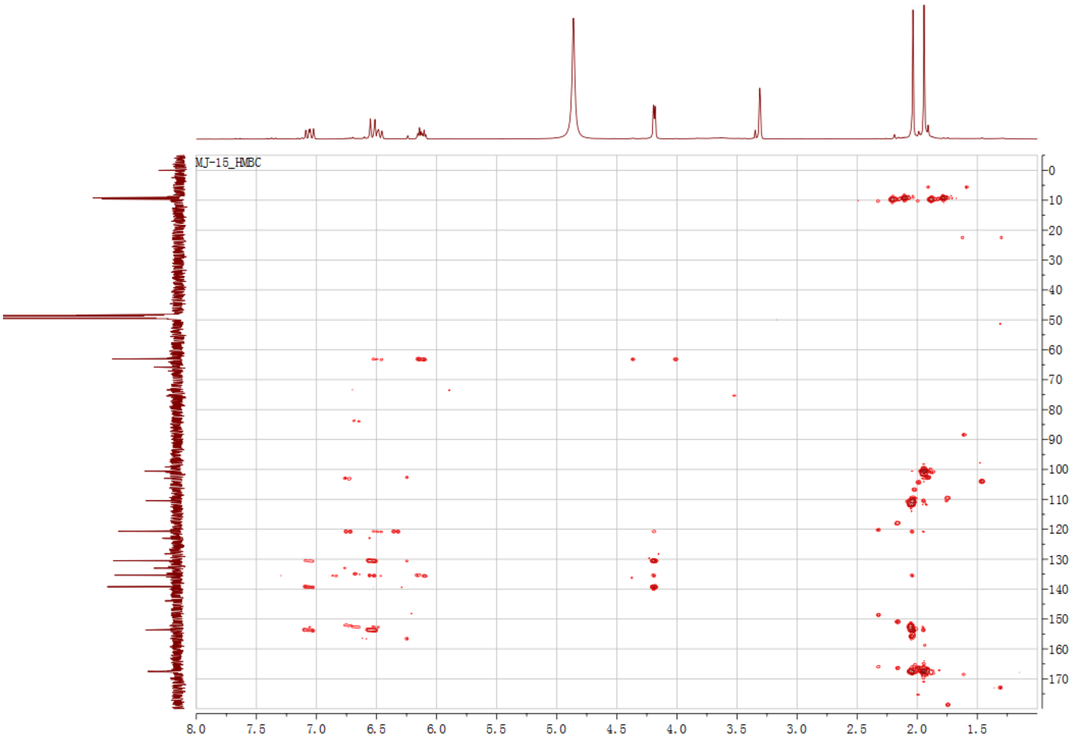

HMBC spectrum of **7**

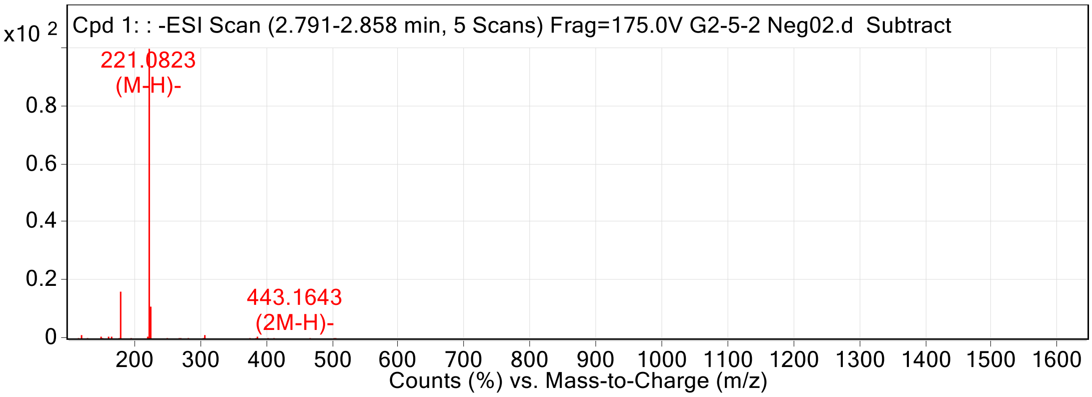

m/z	*Calc m/z*	Diff(ppm)	z	Abund	Formula	Ion
221.0823	221.0819	1.63	-1	234439.7	C12H13O4	(M-H)-

HRESIMS spectrum of **7**

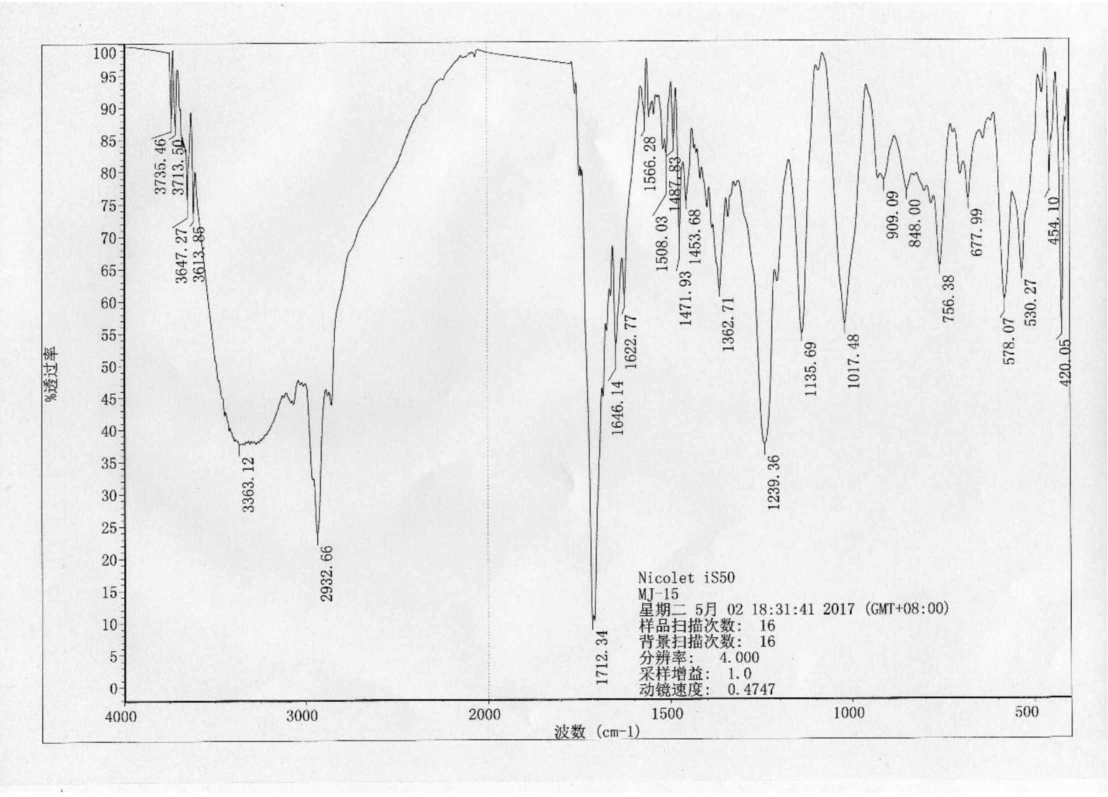

IR spectrum of **7**

NMR, HRESIMS, and IR spectra of 8

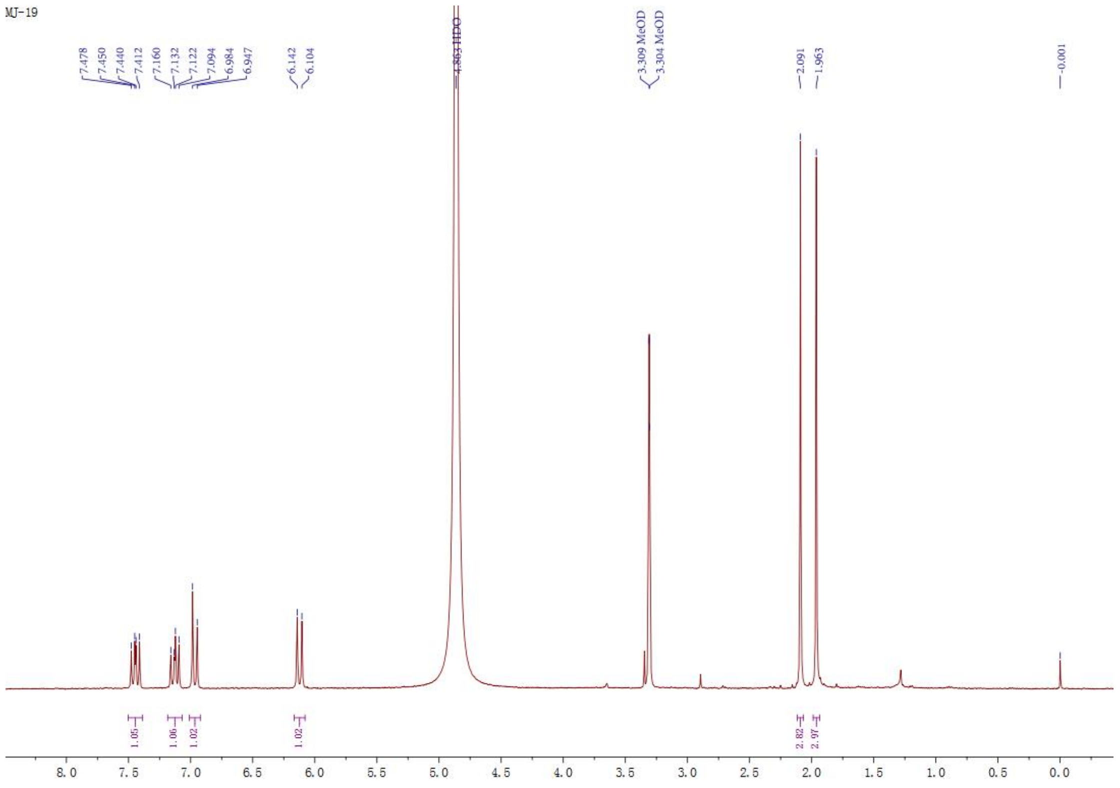

^{1}H NMR spectrum of **8** (CD_3OD, 400MHz)

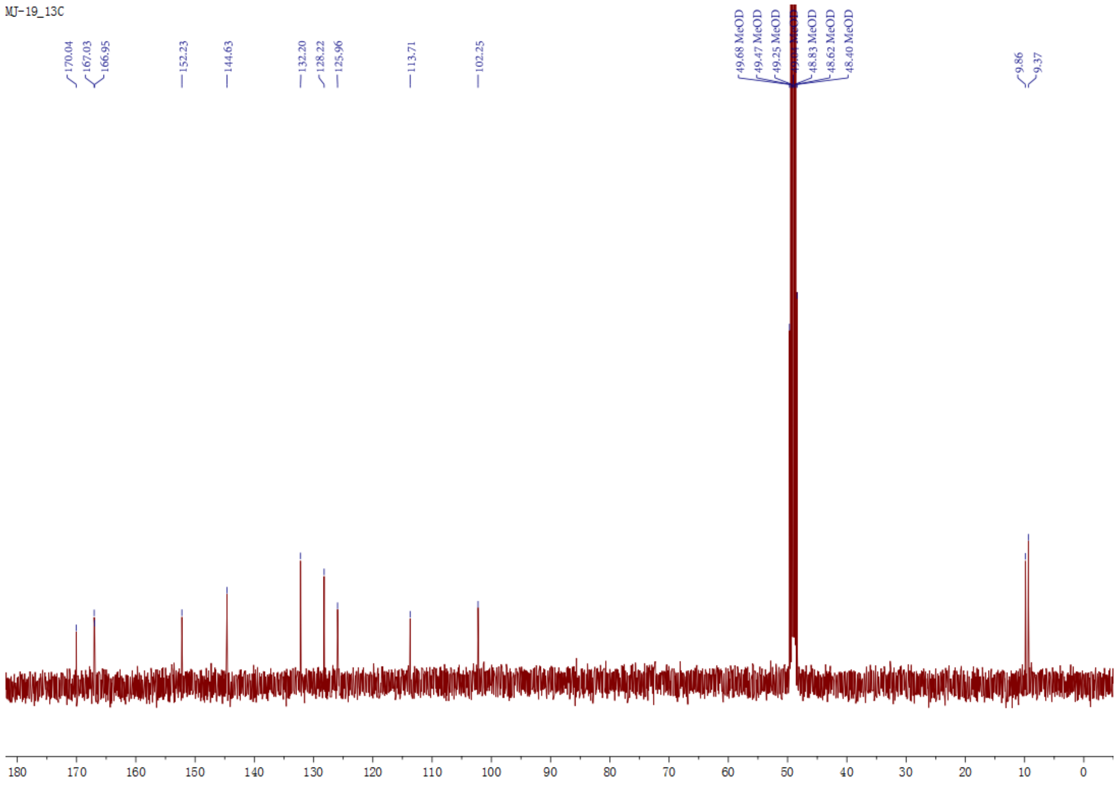

^{13}C NMR spectrum of **8** (CD_3OD, 100MHz)

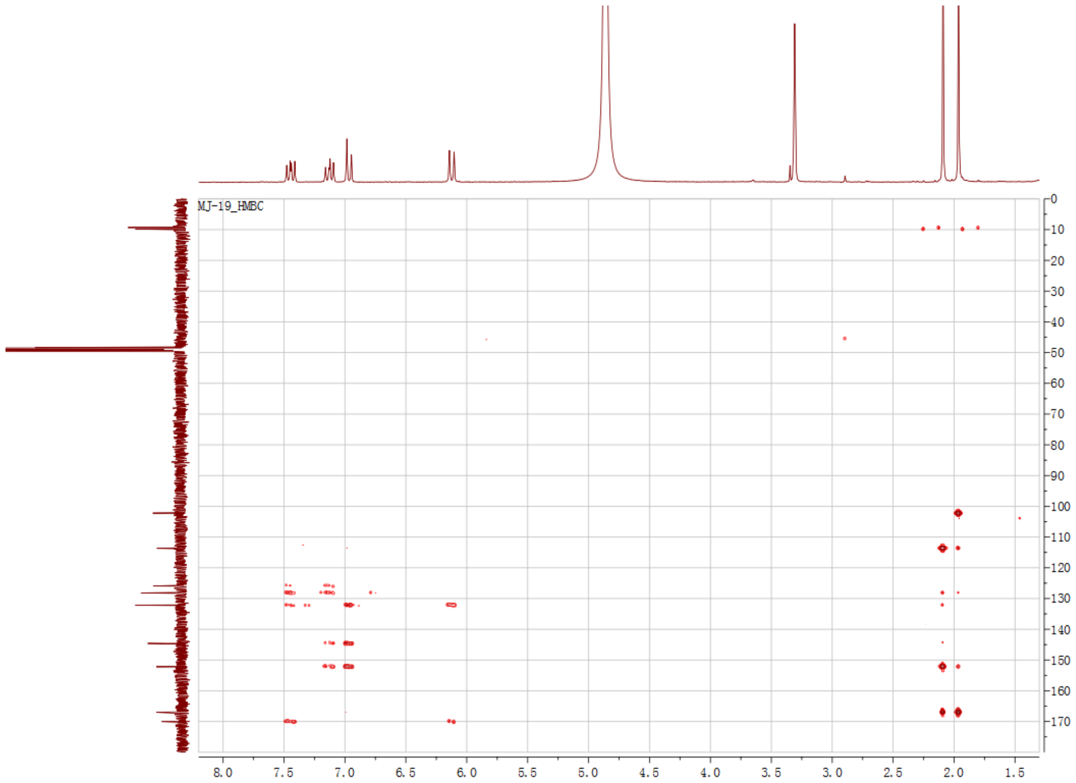

HMBC spectrum of **8**

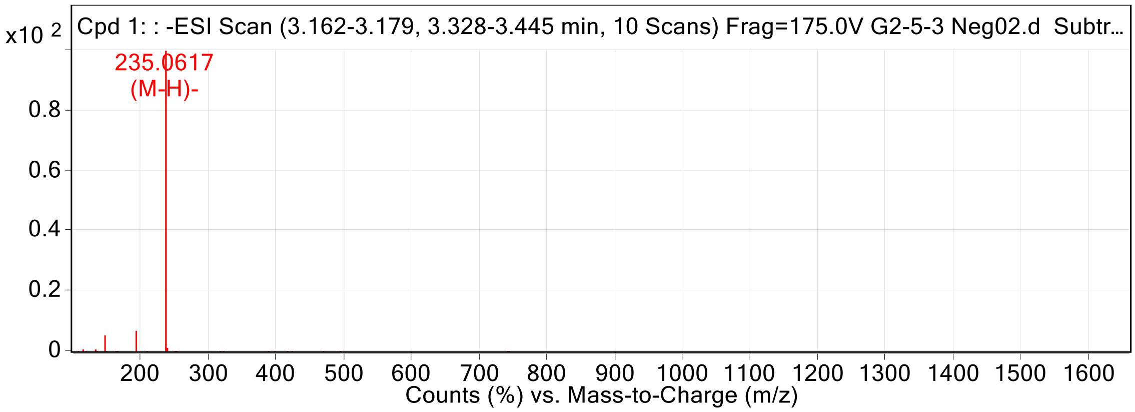

m/z	*Calc m/z*	Diff(ppm)	z	Abund	Formula	Ion
235.0617	235.0612	2	-1	156203	C12H11O5	(M-H)-

HRESIMS spectrum of **8**

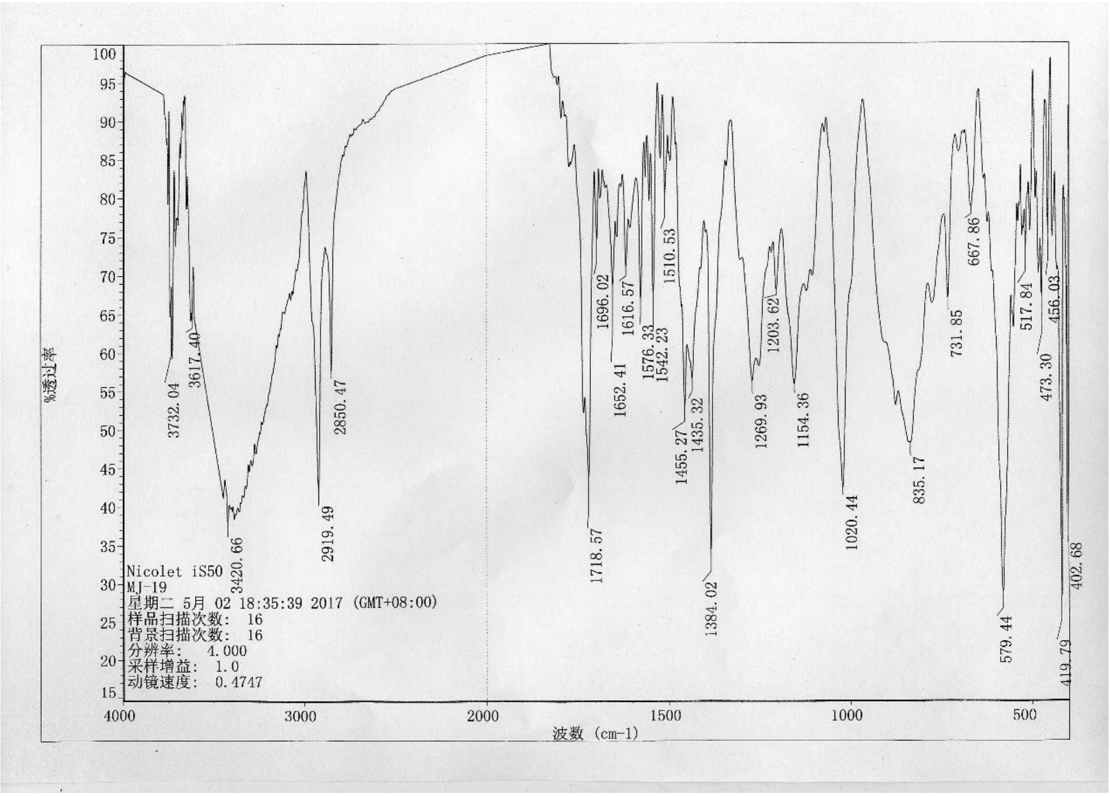

IR spectrum of **8**

NMR, HRESIMS, and IR spectra of 9

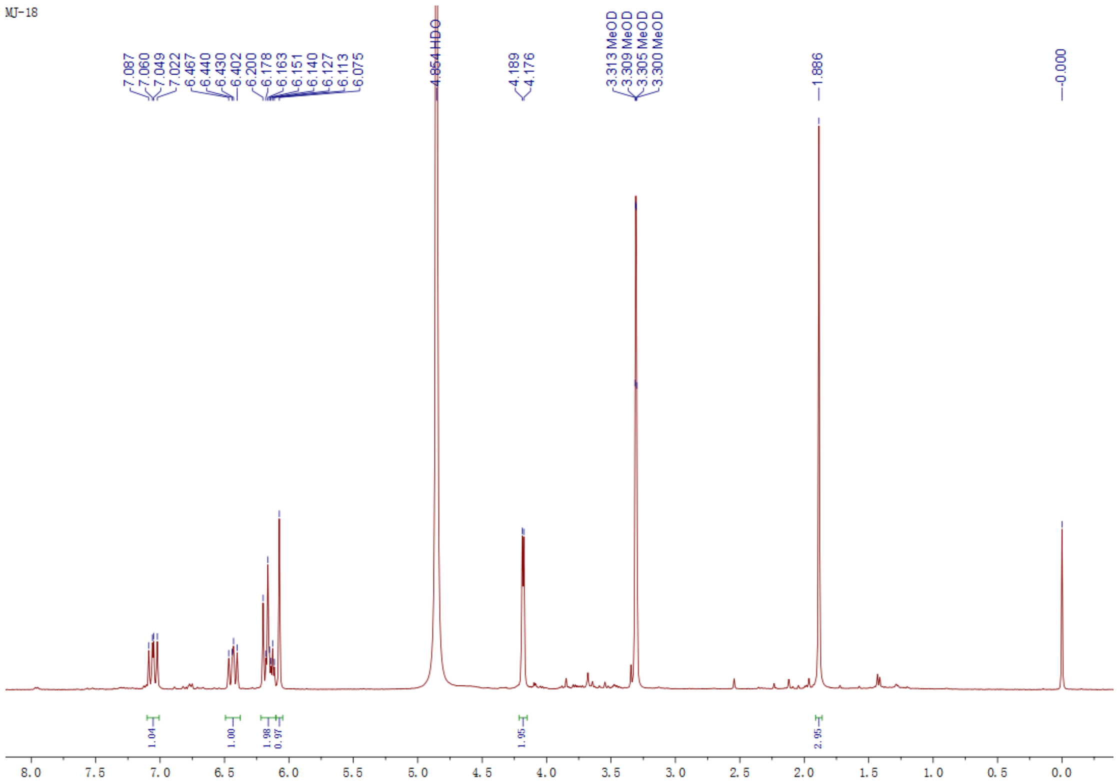

^{1}H NMR spectrum of **9** (CD_3OD, 400MHz)

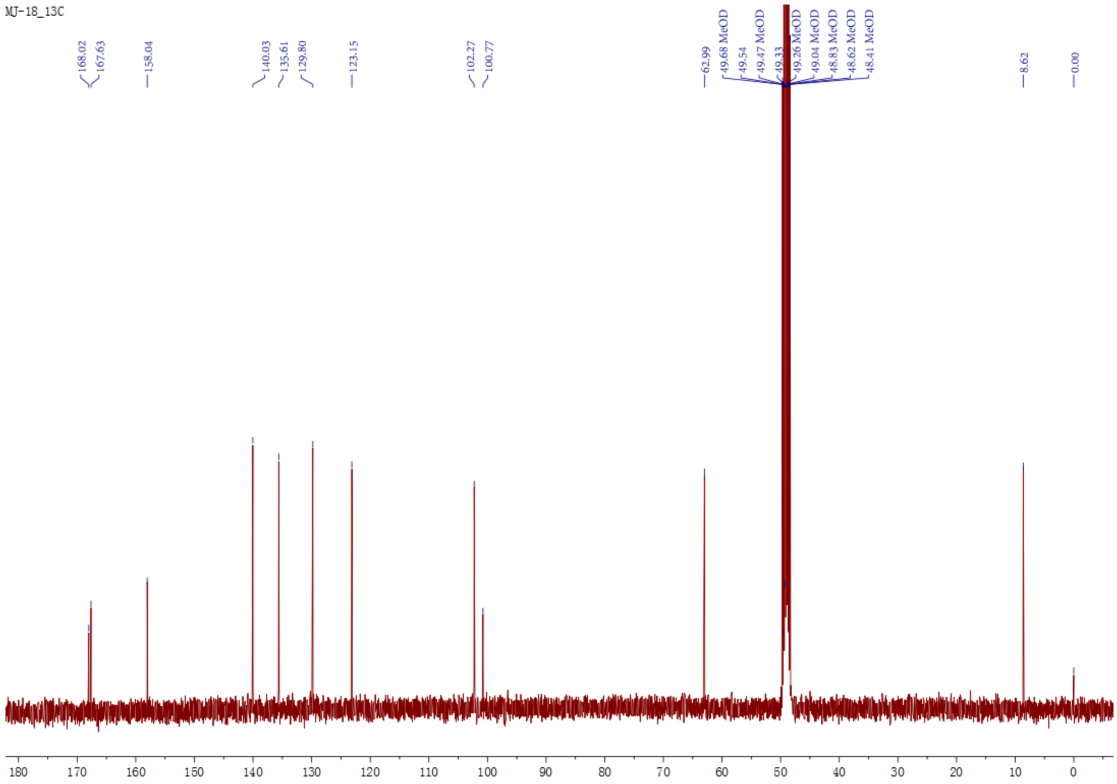

^{13}C NMR spectrum of **9** (CD_3OD, 100MHz)

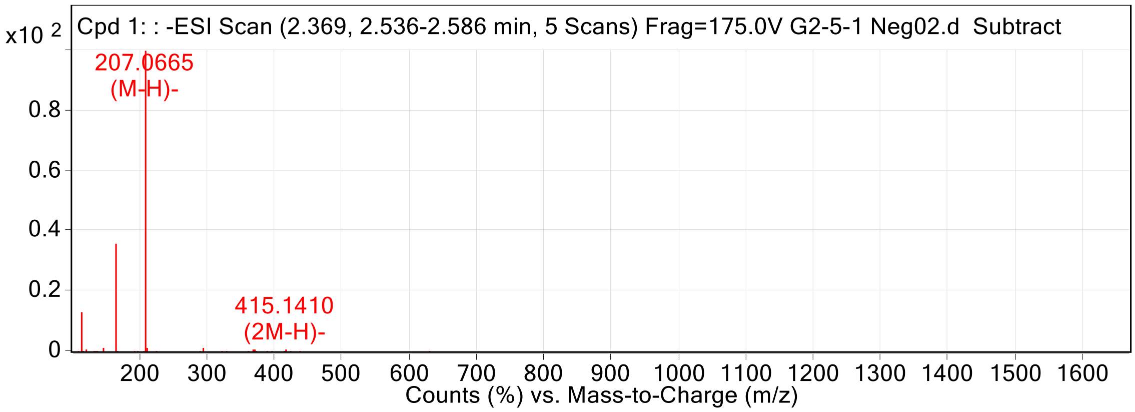

m/z	*Calc m/z*	Diff(ppm)	z	Abund	Formula	Ion
207.0665	207.0663	1.24	-1	188843.5	C11H11O4	(M-H)-

HRESIMS spectrum of **9**

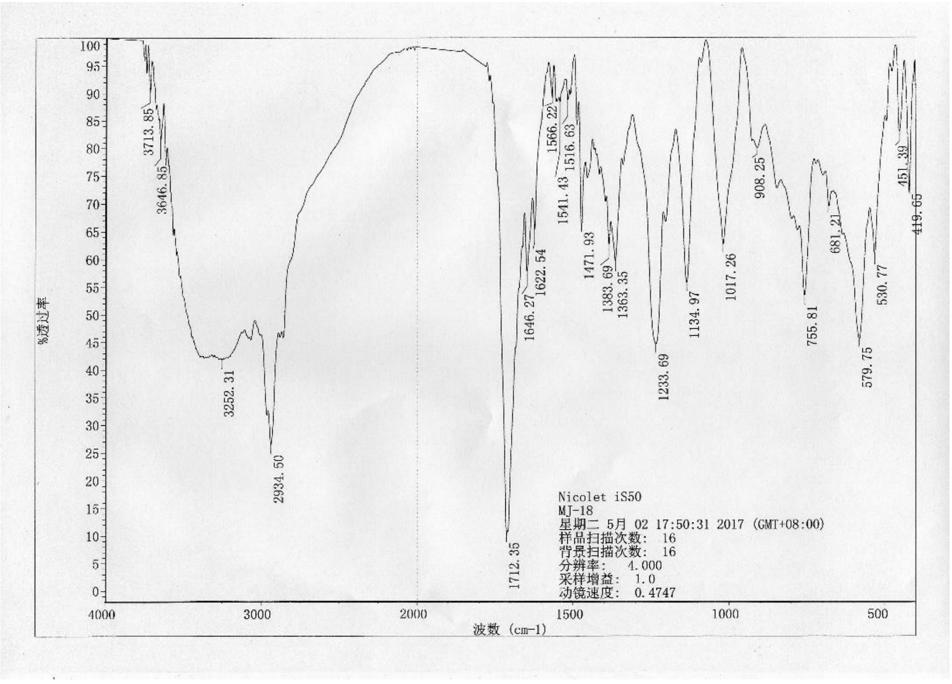

IR spectrum of **9**
